# Adaptive Polymers: The pH-Responsive Revolution in Biomedical Materials

**DOI:** 10.1021/acsomega.6c01425

**Published:** 2026-05-20

**Authors:** Shubham Musale, Suvarna Jarande, Aniket Nikam, Ranjitsinh Pawar, Niyati N. Kodange, Harshad Kapare, Maria Nison, Vasudev R. Pai, Ajinkya Atul Aher, Deepanjan Datta, Prabhanjan Giram

**Affiliations:** † Formulation Development Department, Jodas Expoim Pvt.Ltd., Hyderabad, Telangana 500072, India; ‡ Department of Pharmaceutics, 79253 Dr. D.Y. Patil Institute of Pharmaceutical Sciences and Research, Pune, Maharashtra 411 018, India; § Department of Pharmaceutical Chemistry, All India Shri Shivaji Memorial Society’s College of Pharmacy, Pune, Maharashtra 411 001, India; ∥ Department of Pharmaceutical Chemistry, Dr. D.Y. Patil Institute of Pharmaceutical Sciences and Research, Pune, Maharashtra 411 018, India; ⊥ Department of Pharmaceutics, Poona College of Pharmacy, Bharati Vidyapeeth (Deemed to be University), Erandwane, Pune 411 038, Maharashtra, India; # Department of Pharmaceutics, 548846 National Institute of Pharmaceutical Education and Research (NIPER) S.A.S. Nagar, Sector 67, S.A.S. Nagar (Mohali) 160 062, Punjab, India; ∇ Department of Pharmacognosy, Manipal College of Pharmaceutical Sciences, 76793 Manipal Academy of Higher Education, Manipal 576104, India; ○ Arnold and Marie Schwartz College of Pharmacy and Health Sciences, Long Island University, Brooklyn, New York 11201-5423, United States; ◆ Department of Pharmaceutics, Manipal College of Pharmaceutical Sciences, Manipal Academy of Higher Education, Manipal 576104, India; ¶ Department of Pharmaceutical Sciences, University at Buffalo, The State University of New York, Buffalo, New York 14214, United States

## Abstract

pH-responsive polymers represent a transformative class of smart materials that adapt their physicochemical properties in response to environmental pH changes. This dynamic behavior enables precise control over drug release, tissue interaction, and diagnostic performance, making them highly relevant for advanced healthcare applications. These polymers exploit ionizable functional groups that undergo protonation or deprotonation, triggering conformational changes, solubility shifts, or swelling behavior. Such responsiveness is particularly advantageous in physiological contexts where pH gradients exist, such as the gastrointestinal tract, tumor microenvironments, and intracellular compartments. Recent advances have expanded their utility from simple drug carriers to multifunctional platforms integrating targeting, imaging, and therapeutic functions. Innovations in polymer design, such as block copolymers, hydrogels, and nanostructured assemblies, have improved biocompatibility, tunability, and responsiveness, enabling controlled delivery of small molecules, proteins, and nucleic acids. Furthermore, hybrid systems combining pH-sensitive polymers with inorganic or biological components have opened new avenues for personalized medicine and regenerative therapies. Despite significant progress, challenges remain in achieving predictable *in vivo* performance, scalable synthesis, and regulatory compliance. This review critically examines the molecular mechanisms, material architectures, and biomedical applications of pH-responsive polymers, highlighting emerging trends and future directions. By bridging chemistry and clinical needs, these adaptive materials are poised to revolutionize therapeutic strategies and diagnostic technologies in modern healthcare.

## Graphical abstract

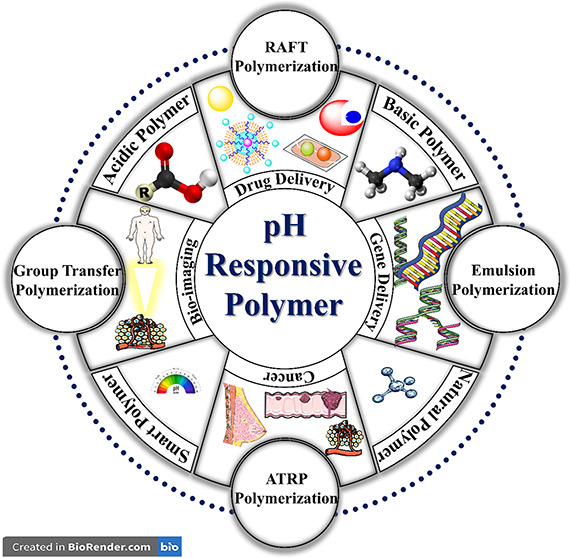

## 1 Introduction

Stimuli-responsive polymers have emerged as a cornerstone of advanced biomedical materials owing to their ability to undergo predictable physicochemical changes in response to environmental cues such as temperature, enzymes, redox conditions, ionic strength, and pH. Among these, pH-responsive polymers have gained prominence because pH gradients are ubiquitous in biological systems, ranging from the acidic tumor microenvironment to variable pH values across the gastrointestinal tract and intracellular organelles.^1,2^ The rational design of pH-sensitive polymeric systems has therefore enabled significant progress in targeted drug delivery, diagnostics, tissue engineering, and biosensing.

pH-responsive polymers are typically defined as macromolecules that undergo reversible conformational, solubility, or swelling changes when exposed to variations in environmental pH. These changes arise due to the ionization or protonation of functional groups such as carboxylic acids, amines, imidazoles, or sulfonamides incorporated within the polymer backbone or side chains.^3^ The pH responsiveness can be finely tuned by manipulating polymer composition, molecular weight, architecture, and p*K*_a_ of ionizable moieties, making them highly versatile platforms for biomedical applications. Common architectures include linear polymers, branched and star-shaped polymers, block and graft copolymers, and cross-linked polymer networks such as hydrogels.^4–7^ Linear pH-sensitive polymers, for instance, poly­(acrylic acid) (PAA) or poly­(methacrylic acid) (PMAA), demonstrate predictable ionization-driven conformational changes and have been extensively investigated for oral and mucosal drug delivery systems.^8,9^ Block copolymer architectures, such as poly­(ethylene glycol)-*block*-poly­(histidine) or poly­(caprolactone)-*block*-poly­(β-amino ester), enable the formation of self-assembled micelles or vesicles, which are particularly attractive for site-specific drug delivery in acidic tumor or endosomal environments.^10,11^ More complex architectures, including star polymers,^12^ dendrimers,^13,14^ and polymer brushes,^15^ offer enhanced responsiveness due to high functional group density and tunable p*K*_a_ values. Dendritic pH-responsive systems have been shown to exhibit sharp on–off transitions and improved cellular uptake.^16–18^ Furthermore, interpenetrating polymer networks (IPNs) and core–shell hydrogel structures allow independent control of mechanical strength and pH sensitivity, expanding their application in controlled release and tissue engineering.^19^

pH-responsive polymers are broadly classified into anionic, cationic, and zwitterionic systems based on the nature of ionizable groups. Anionic polymers, such as poly­(acrylic acid), poly­(methacrylic acid), and alginate, contain weak acidic groups that ionize at higher pH, leading to chain expansion due to electrostatic repulsion.^20,21^ In contrast, cationic polymers, including chitosan, poly­(ethylene imine), and poly­(l-histidine), undergo protonation under acidic conditions, resulting in increased solubility or swelling.^8,22^ Zwitterionic pH-responsive polymers combine both functional groups, offering sharper transition profiles and reduced nonspecific protein adsorption, particularly advantageous for *in vivo* applications.^23,24^ The mechanism underlying pH responsiveness primarily involves ionization-induced physicochemical transformations. Upon exposure to a triggering pH, polymer chains undergo protonation or deprotonation, causing alterations in hydrophilicity, chain conformation, intermolecular interactions, and network swelling. In hydrogel systems, this leads to dramatic volume changes, while in nanoparticles or micelles, it can trigger destabilization and payload release.^25^ Additionally, pH-cleavable linkages such as hydrazone, acetal, or imine bonds are frequently incorporated to enable chemical degradation or cargo release under acidic microenvironments, such as those found in tumors or lysosomes.^26^

The principal advantage of pH-responsive polymers lies in their ability to achieve site-specific and controlled therapeutic delivery, thereby minimizing off-target toxicity and improving therapeutic efficacy. These polymers allow for passive targeting through physiological pH variations without requiring external triggers, enhancing patient compliance.^3^ Furthermore, their modular chemistry enables compatibility with a wide range of drugs, including small molecules, proteins, nucleic acids, and imaging agents.^27^ Covalent integration of synthetic polymers with biomolecules, including poly­(amino acids), peptides, or proteins, enables the formation of biohybrid materials that synergistically combine the tunability of synthetic polymers with the biological functionality of natural systems. To this end, biodegradability, biocompatibility and the ability to self-assemble into well-defined three-dimensional nanostructures of naturally derived and synthetic pH-responsive polymers further strengthen their translational potential.^28^ Despite their promising attributes, pH-responsive polymers face several limitations. One major challenge is the relatively narrow pH difference between healthy and pathological tissues, particularly in cancer, which may limit responsiveness and selectivity.^29^ Additionally, premature ionization, instability in physiological fluids, batch-to-batch variability, and scale-up difficulties remain significant barriers to clinical translation. Certain cationic polymers also exhibit cytotoxicity due to membrane disruption, necessitating careful polymer design and surface modification.^30^

pH-responsive polymers have demonstrated extensive utility across diverse biomedical domains. In drug delivery, they are widely employed for oral, parenteral, and intracellular delivery systems that respond selectively to gastrointestinal or tumor pH.^31^ In gene delivery, pH-sensitive cationic polymers facilitate endosomal escape through the “proton sponge” effect, improving transfection efficiency.^32^ Additionally, pH-responsive hydrogels are increasingly used in tissue engineering, wound healing, and biosensors, where dynamic responsiveness to physiological conditions is critical for functionality and feedback.^33^ Overall, pH-responsive polymers represent a dynamic and rapidly evolving class of smart biomaterials. Advances in polymer chemistry, nanotechnology, and biological understanding are expected to overcome existing limitations and expand their clinical impact. Continued interdisciplinary research will be essential to translate these materials from experimental systems to reliable and scalable biomedical solutions.

## 2 Mechanism of pH-Responsiveness

The pH-responsive behavior of polymers arises primarily from the presence of ionizable functional groups, such as carboxylic acids, sulfonic acids, amines, or imidazole moieties, along the polymer backbone or side chains.^34^ These groups undergo reversible protonation–deprotonation in response to environmental pH changes, leading to alterations in polymer charge density, solubility, chain conformation, and hydrophilic–hydrophobic balance.^35^ In acidic or basic environments, this ionization induces electrostatic repulsion or attraction, resulting in polymer swelling, deswelling, or dissociation of supramolecular assemblies such as micelles and nanoparticles. Weak polyelectrolytes, including PAA and poly­(dimethylaminoethyl methacrylate) (PDMAEMA), exhibit especially pronounced pH sensitivity due to their tunable p*K*_a_ values near physiological pH.^36,37^ In block copolymer systems, pH-responsiveness often triggers core destabilization or shell shedding, enabling controlled drug release in acidic intracellular compartments such as endosomes and lysosomes.^38^ Additionally, pH-labile linkages, such as hydrazone, acetal, or imine bonds, can be incorporated into polymer backbones or cross-links to enable chemical degradation under specific pH conditions, further enhancing controlled release precision.^39,40^ Figure 1 and Table 1 depict the comparative and mechanistic summary of representative pH-responsive polymers employed in drug delivery systems, categorized into basic (synthetic) polymers, natural polymers, and block copolymers, highlighting their pH-triggered behavior and relevance to controlled and targeted therapeutic delivery.

**Figure 1:**
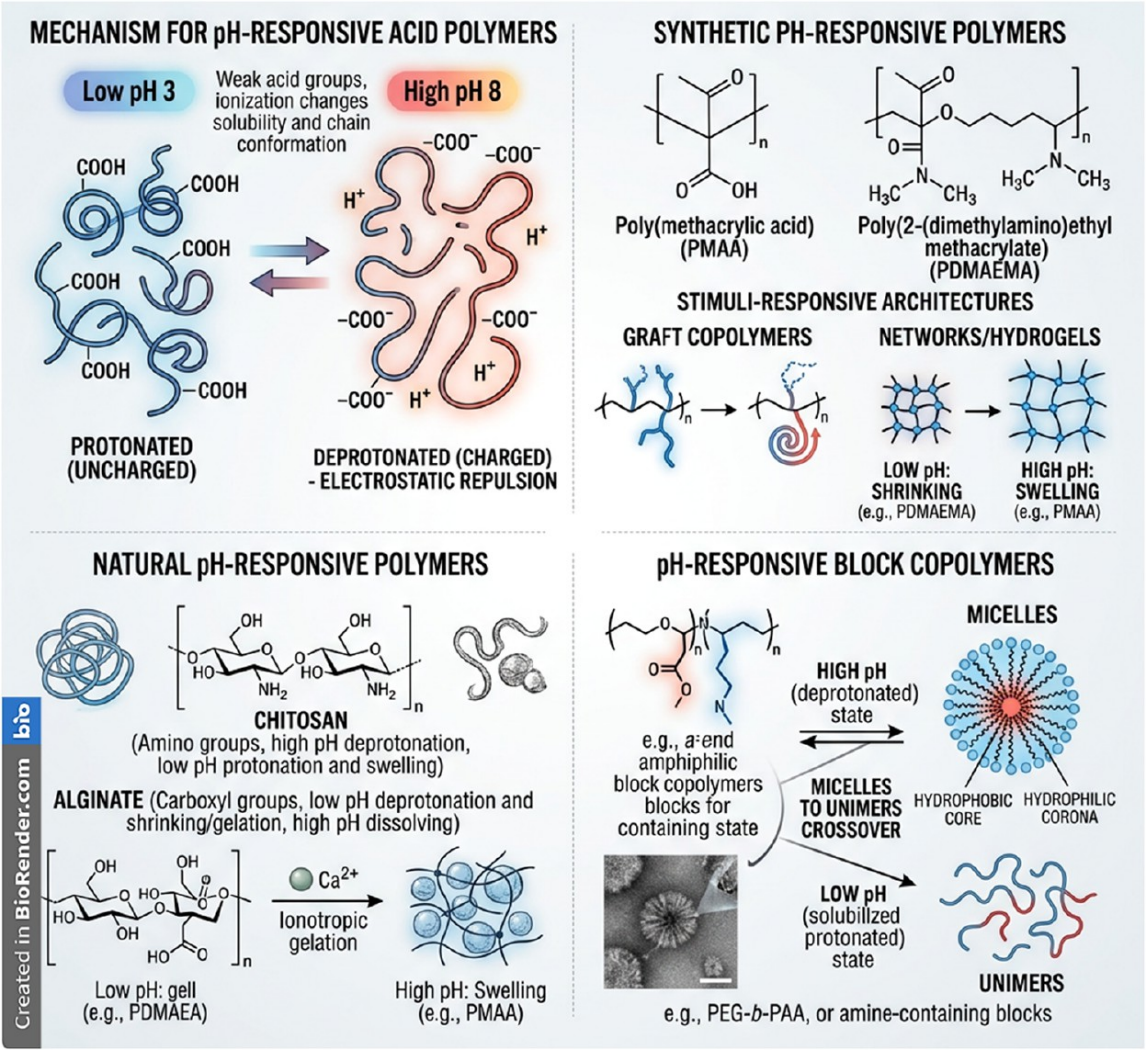
pH-responsive polymers: structures and response mechanisms. Schematic illustration of the ionization-driven conformational changes of pH-responsive polymers, highlighting protonation at low pH and deprotonation at high pH that modulate chain conformation, electrostatic interactions, and swelling behavior. Representative synthetic (e.g., PMAA, PDMAEMA) and natural polymers (e.g., chitosan, alginate) are shown, along with common stimuli-responsive architectures including graft copolymers, networks/hydrogels, and block copolymer micelles. These systems undergo reversible pH-dependent transitions between collapsed/shrunken and swollen/dissociated states, enabling controlled assembly, sol–gel transitions, and drug release. This image has been created as a Creative Common using the BioRender software (https://www.biorender.com/).

**Table 1:** Comparative Summary of Representative pH-Responsive Polymers Used in Drug Delivery Systems, Classified as Acidic, Basic (Synthetic), Natural, and Block Copolymers^a^

Polymers and Trigger pH	Advantages	Challenges	Biodegradability	Toxicity	Therapeutic Release Pattern	Typical Release Kinetics	ref
pH-responsive acidic polymers							
PAA Ionization and swelling at pH ≥ 5.5–6.5; responsive in intestinal and tumor pH	Sharp pH-dependent swelling, strong hydrogen-bond-mediated drug loading, mucoadhesive	Excessive swelling, weak mechanical integrity if uncross-linked	Low–moderate (higher in cross-linked hydrogels)	Low	Minimal release at acidic pH; rapid swelling-mediated release at neutral/tumor pH	Swelling-controlled/Higuchi	^8,84,85^
PMAA Deprotonation at pH ≥ 6.0–7.0	Strong reversible conformational transition, tunable pH sensitivity	Brittle alone; usually requires copolymerization	Low	Low	Diffusion-controlled release following chain expansion	Diffusion-controlled/Korsmeyer–Peppas	^8,86,87^
Eudragit L & S (methacrylic acid copolymers) pH ≥ 6.0 (L); pH ≥ 7.0 (S)	FDA-approved, reproducible enteric and colon targeting, excellent gastric protection	Nonbiodegradable; limited flexibility in trigger pH	Nonbiodegradable	Very low	Near-zero gastric release; sharp intestinal/colonic burst release	First-order dissolution	^88–90^
CMC Progressive swelling at pH ≥ 6.0–7.4	Biocompatible, easy chemical modification, and high-water uptake	Gradual dissolution; weak pH sharpness	High	Very low	Sustained swelling-mediated release in intestinal pH	Higuchi/erosion-controlled	^91,92^
Poly(itaconic acid) Ionization at pH ≥ 5.5–6.8	Strong ionic responsiveness, dual carboxyl groups enhance swelling	Limited commercial availability	Moderate	Low	pH-triggered expansion with controlled diffusion	Swelling + diffusion	^93–95^
Alginate (acidic derivatives/alginic acid systems) Enhanced swelling at pH ≥ 6.8–7.4	Colon targeting, mild gelation, excellent biocompatibility	Requires coating/cross-linking for gastric stability	High	Very low	Suppressed gastric release with controlled intestinal/colon release	Swelling-controlled	^96–98^
pH-responsive basic (synthetic) polymers							
PEI pH 4.5–6.5 (endosome/lysosome))	High proton-buffering capacity (“proton sponge”), excellent gene/drug complexation	High charge-induced cytotoxicity, nonbiodegradable	Poor (branched/linear forms)	Moderate–high, MW-dependent	Rapid release under acidic endosomal pH (5–6)	Diffusion + polymer swelling (Korsmeyer–Peppas)	^8,34^
PBAEs (pH 5.0–6.8)	Tunable p*K*_a_, biodegradable via ester cleavage, and high transfection efficiency	Limited long-term stability, batch variability	High	Low–moderate	Acid-triggered intracellular burst release	First-order/erosion-controlled	^99,100^
PDMAEMA (pH 5.5–7.0)	Sharp pH-responsiveness near physiological pH, easy chemical modification	Partial cytotoxicity at high charge density	Limited unless copolymerized	Moderate	Sustained release in acidic tumor/endosomal pH	Higuchi + Peppas	^101^
PDEAEMA (pH 5.0–6.5)	Strong pH-triggered micellar disassembly	Hydrophobic aggregation needs PEGylation	Limited	Moderate	pH-triggered fast release at pH < 6	Swelling-diffusion-controlled	^102,103^
pH-responsive basic (synthetic) polymers							
CH (pH < 6.2 (protonated); release ↑ at pH 6.8–7.4)	Biocompatible, mucoadhesive, protonated in acidic pH	Poor solubility at neutral pH, batch variability	High (enzymatic degradation)	Very low	Suppressed release in gastric pH, enhanced release at intestinal/tumor pH	Diffusion + erosion (Peppas)	^104,105^
Alginate (pH 6.8–7.4 (swelling/dissociation)	Gel-forming, mild cross-linking, colon-targeting capability	Weak mechanical strength alone	High	Very low	Minimal release in acidic pH, controlled swelling-mediated release at neutral pH	Higuchi/swelling-controlled	^8,106^
Gelatin pH 5.0–7.4 (combined pH + enzymatic)	Enzyme-responsive + pH sensitivity, good cell interaction	Thermal instability, batch inconsistency	High	low	Sustained release with enzymatic acceleration	First-order/erosion	^107,108^
Cellulose derivatives (e.g., HPMC-phthalate) (pH ≥ 6.5)	FDA-approved, excellent enteric targeting	Limited responsiveness range	Moderate-High	Very low	Minimal gastric release, sharp intestinal burst	First-order	^109^
pH-responsive block copolymers							
PEG-*b*-PDMAEMA (pH 5.5–6.8)	Self-assembling micelles, reduced toxicity via PEG shell	PEG shedding at low pH	Partial	Low–moderate	Acid-triggered micelle destabilization	Diffusion-controlled	^45,101^
PEG-*b*-PDEAEMA (pH 5.0–6.5)	Sharp tumor-pH specificity	Core aggregation, if unbalanced	Partial	Moderate	Rapid release at pH 5–6	Peppas (anomalous transport)	^102^
PEG-*b*-PBAE (pH 5.0–6.8)	Fully biodegradable, high drug loading	Synthetic complexity	High	Low	Endosomal burst + sustained release	Erosion + diffusion	^110,111^
Triblock pH-responsive copolymers (PEG–PDMAEMA-P (HEMA-FBA)) pH 5.0–6.0 (acid-labile linkage cleavage)	Multidrug/gene codelivery	Scale-up limitations	Partial	Low	Sequential pH-triggered release	First-order + Peppas	^112^

a **PAA:** Poly­(acrylic acid)**; PMAA:** Poly­(methacrylic acid)**; CMC:** Carboxymethyl Cellulose**; PEI:** Polyethylenimine**; PBAEs:** Poly­(β-amino esters)**; PDMAEMA:** Poly­(2-(dimethylamino)­ethyl methacrylate)**; PDEAEMA:** Poly­(2-(diethylamino)­ethyl methacrylate)**; CS:** Chitosan**; HPMC:** Hydroxypropyl Methylcellulose**; PEG-** *b* **-PDMAEMA:** Polyethylene glycol-*block*-poly­(2-(dimethylamino)­ethyl methacrylate)**; PEG-** *b* **-PDEAEMA:** Polyethylene glycol-*block*-poly­(2-(diethylamino)­ethyl methacrylate)**; PEG-** *b* **-PBAE:** Polyethylene glycol-*block*-poly­(β-amino ester)**; PEG–PDMAEMA-P:** Polyethylene glycol–poly­(2-(dimethylamino)­ethyl methacrylate)–polymer (P)**; HEMA-FBA:** conjugate formed from 2-hydroxyethyl methacrylate (HEMA) and 4-formylbenzoic acid (FBA).

## 3 Fabrication Approaches for pH-Responsive Polymer Systems

A variety of fabrication techniques have been developed to translate pH-responsive polymers into functional drug delivery systems and biomedical devices (Figure 2). Free-radical polymerization, controlled/living polymerization methods (such as ATRP, RAFT, and NMP), and ring-opening polymerization are commonly employed to synthesize polymers with precise molecular weights and compositions.^41–43^ These approaches enable fine control over polymer architecture, which is essential for achieving predictable pH responses. Hydrogels are often fabricated via chemical or physical cross-linking, allowing modulation of mesh size and swelling behavior as a function of pH.^44^

**Figure 2:**
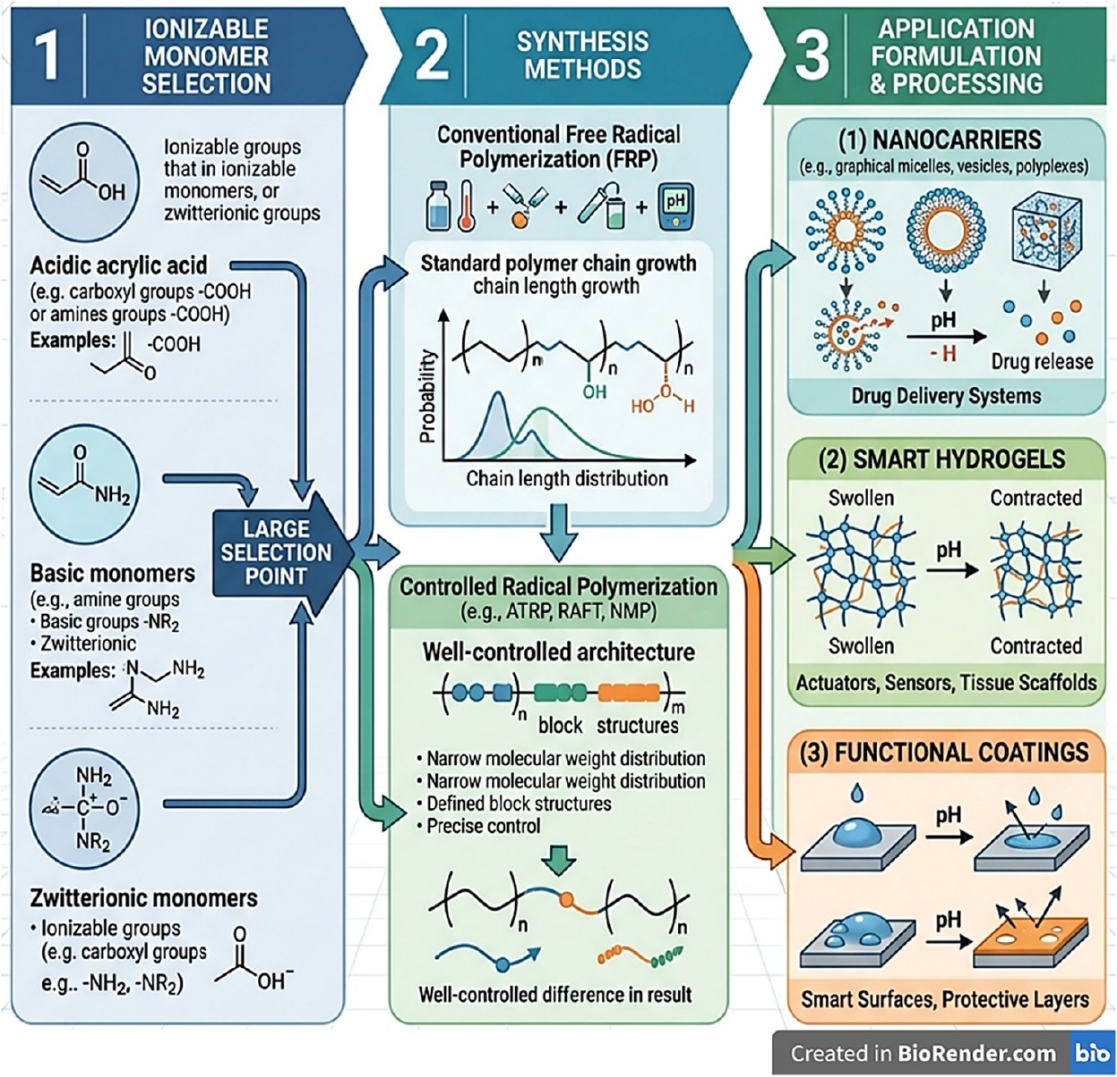
Schematic representation of the fabrication and application workflow for pH-responsive polymers. The process initiates with the selection of ionizable monomers (Stage 1), followed by synthesis via either conventional Free Radical Polymerization or Controlled Radical Polymerization (Stage 2) to achieve specific molecular architectures. The resulting materials are processed into nanocarriers, smart hydrogels, or functional coatings (Stage 3). This image has been created as a Creative Common using the BioRender software (https://www.biorender.com/).

For nano- and microscale systems, self-assembly, emulsion polymerization, ionic gelation, and layer-by-layer (LbL) assembly are widely utilized. Self-assembly of amphiphilic block copolymers yields pH-responsive micelles and polymersomes with high drug-loading efficiencies.^45^ LbL techniques using oppositely charged weak polyelectrolytes allow precise control over film thickness and pH-triggered permeability, making them useful for coatings and capsules.^46,47^ Advances in microfluidics and 3D printing have further enabled reproducible fabrication of pH-responsive scaffolds and particles with well-defined geometries, opening new avenues for personalized and site-specific therapeutic systems.^48–50^

## 4 Decoding pH-Responsive Polymers: A Comprehensive Classification Framework

pH-responsive polymers have emerged as a pivotal class of smart materials due to their ability to undergo predictable physicochemical transformations in response to environmental pH variations. These polymers have found extensive applications in drug delivery, tissue engineering, biosensing, and biomedical coatings, yet the rapidly expanding and diverse body of literature often lacks a unified framework for systematic classification. Decoding pH-Responsive Polymers: A Comprehensive Classification Framework aims to address this gap by presenting an integrated and structured overview of pH-responsive polymer systems based on their molecular architecture, response mechanisms, and fabrication strategies. The framework categorizes polymers according to architectural complexity, including linear, branched, block copolymeric, dendritic, and cross-linked network systems, and correlates these structures with functional performance. Mechanistic classification is further established based on ionization-driven swelling and deswelling, solubility transitions, conformational changes, and pH-labile bond cleavage. Additionally, the work surveys contemporary fabrication approaches, such as controlled polymerization, self-assembly, hydrogel cross-linking, and layer-by-layer assembly, that enable precise tailoring of pH responsiveness across multiple length scales. By consolidating these dimensions into a cohesive classification scheme, the following sections provide clarity for material selection and rational design of next-generation pH-responsive platforms.

### 4.1 pH-Responsive Acidic Polymers

Polymers containing weakly basic or weakly acidic functional residues are commonly employed as pH-responsive polymers. These polymers bear ionizable pendant groups that undergo reversible protonation and deprotonation in response to environmental pH changes.^51^ Weak acidic groups tend to release protons at higher pH values, whereas weak basic groups accept protons under acidic conditions. As reported in earlier studies, both ionic and nonionic transitions play a critical role in modulating polymer–water interactions, thereby allowing fine control over polymer hydrophilicity in aqueous media. Depending on the pH, polymer chains may undergo solubilization or precipitation, leading to the formation of hydrogels that exhibit reversible swelling and deswelling behavior.^22,52,53^ These pH-induced transitions are accompanied by marked changes in the hydrophilic–hydrophobic balance of polymeric particles as well as alterations in their surface properties. Based on the nature of their ionizable functionalities, pH-responsive polymers can be classified according to their specific functional groups, as discussed below.^54^

#### 4.1.1 Carboxylic Groups

Deprotonation of acidic functional groups at basic pH values leads to the formation of anionic polyelectrolytes, particularly in polymers containing carboxylic acid moieties. Polymers such as poly­(methacrylic acid) and poly­(acrylic acid), along with related pH-responsive polyacids, have been widely reported in the literature for their pronounced pH sensitivity.^34,55,56^ Both methacrylic acid and acrylic acid monomers are readily polymerized using a variety of polymerization techniques, including free-radical and controlled/living polymerization methods.^43^ In certain polymerization strategies, carboxylic acid functionalities are temporarily protected to prevent premature ionization or side reactions during polymerization; these protecting groups are subsequently removed through deprotection chemistry to restore the pH-responsive carboxyl groups.^57^ Additionally, pH-responsive dendrimers have been designed to present carboxyl-terminated groups on their periphery, enabling tunable ionization behavior, enhanced solubility control, and responsive structural transitions in aqueous environments (Figure 3a).^58,59^

**Figure 3:**
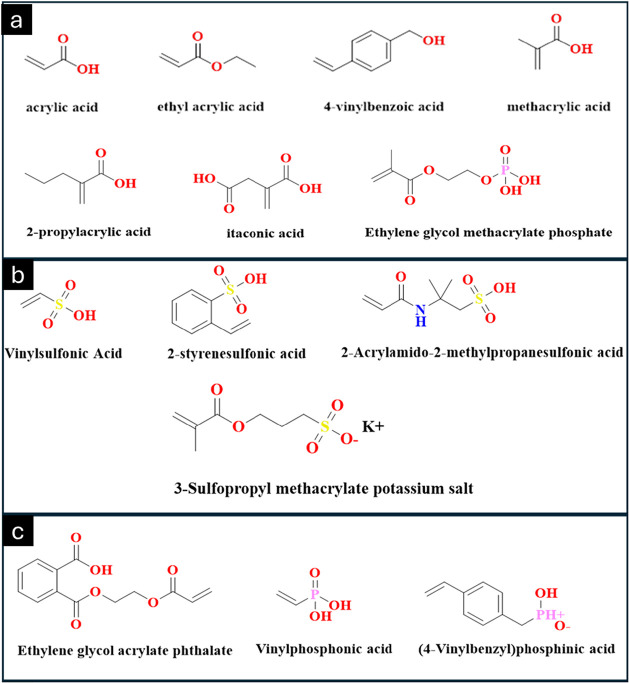
Schematic illustration of representative monomers that incorporate carboxylic functional groups, which impart pH-responsive behavior to polymer systems. The presence of ionizable −COOH groups enables protonation and deprotonation under varying pH conditions, leading to changes in solubility, swelling, and overall polymer conformation (a). Representative monomers incorporate sulfonic acid (−SO_3_H) functional groups, which confer strong acidic character and exceptional hydrophilicity to polymer systems (b). Phosphorus-containing monomers showcase representative monomers that incorporate phosphorus-based functional groups, such as phosphate, phosphonate, and phosphoric acid derivatives. These moieties impart unique properties to polymer systems, including strong ionic character, high hydrophilicity, and excellent buffering capacity (c). The structures were prepared using ChemDraw Professional 16.0.

#### 4.1.2 Sulfonic Acid

Poly­(4-styrene sulfonic acid) and poly­(2-acrylamide-2-methylpropanesulfonic acid) are both commonly used polymers present in sulfonic acid.^60^ For the preparation of hydrogel, commonly chosen polymers have a group of sulfonic acid. When pH is more than the p*K*_a_ value, the hydrogel with sulfonic acid side groups swells remarkably. These groups are more hydrophilic in their anionic form (Figure 3b).

#### 4.1.3 Phosphonic Acid

Phosphorus-containing (meth)­acrylate monomers are widely used. For the synthesis of hydrogel, these types of monomers have been commonly used for many years^60^ and swollen in basic pH conditions.^61,62^ Synthetic and natural polymers treated with several phosphonic acids are selected for the synthesis of phosphonic acid-functionalized polymers (Figure 3c).^63^

#### 4.1.4 Boronic Acids

Boronic acids function as Lewis acids, with their reactivity strongly influenced by the surrounding chemical environment. Carbon-bound BAs are susceptible to oxidative cleavage (e.g., by hydrogen peroxide), whereas oxygen-bound BAs exhibit distinct hydrolytic stabilities. In their neutral state, BAs adopt a trigonal-planar, *sp* ^2^-hybridized boron center bonded to an alkyl or aryl group and two hydroxyl groups, resulting in a six-electron valence configuration. In aqueous media, boronic acids (and BA-containing polymers) exist in equilibrium between this neutral, hydrophobic form and a hydrophilic hydroxyboronate anion formed via hydroxide coordination (Figure 4a).^64^

**Figure 4:**
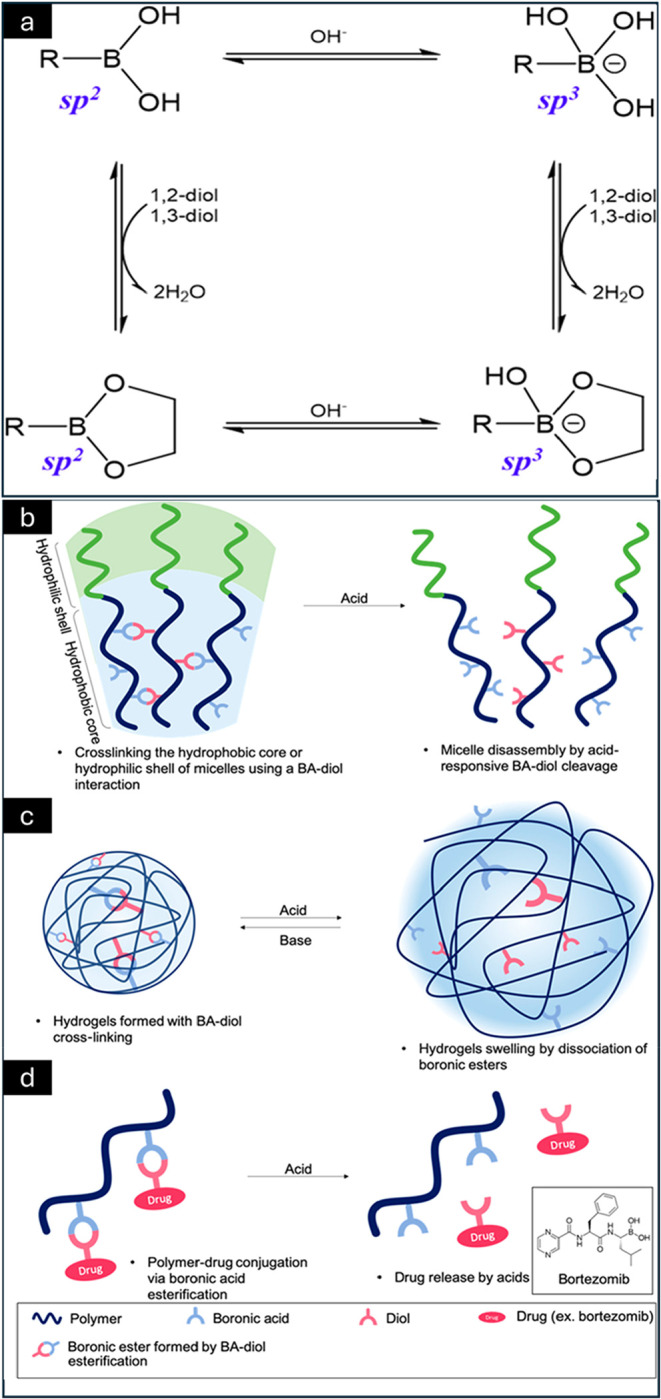
Equilibrium between boronic acids and diols. The orbital hybridization state of boron in each form is indicated in blue (a). Schematic overview of pH-responsive polymeric systems incorporating boronic acids (BAs). pH-responsive micelles formed from BA-functionalized polymers, stabilized via hydrophobic core or hydrophilic shell cross-linking through BA–diol esterification; acidic conditions trigger micelle destabilization via boronic ester cleavage (b). Chemically cross-linked BA-based hydrogels that swell or dissociate through reversible, acid-responsive BA–diol interactions (c). Polymer–drug conjugates (e.g., glycoproteins, bortezomib) employing BA linkers to enable acid-responsive drug release (d). Reprinted with permission from ref 64. Copyright 2019, American Chemical Society.

Polymers containing boronic acid functional groups have attracted significant attention due to their utility in applications such as glucose sensing,^65^ self-healing hydrogels,^66^ and stimuli-responsive materials.^67^ Physiological pH is tightly regulated under normal metabolic conditions; however, pathological states such as tumors and inflammation are associated with elevated metabolic activity and localized pH dysregulation.^68^ Additionally, endosomal compartments exhibit acidic pH values (≈5.0–6.8), and since nanoparticle uptake commonly occurs via endocytosis, acidic pH serves as an effective trigger for intracellular drug delivery.^69^ Figure 4b–d illustrates the pH-responsive dissociation mechanisms of boronic acid-containing materials, including polymeric micelle disassembly and hydrogel swelling. Polymers incorporating phenylboronic acid moieties [Ph–B­(OH)_2_] are the most extensively studied and commonly reported in the literature.^70^ Boronic acid–functionalized monomers can be polymerized using a variety of polymerization techniques, including free-radical polymerization^71^ and controlled/living polymerization^43^ methods. However, due to the susceptibility of boronic acid groups to side reactions and hydrolysis during polymerization, recent studies increasingly employ protected boronic acid monomers. Following polymerization, the boronic acid functionality is restored through postpolymerization deprotection chemistry, enabling precise control over polymer structure and pH- or glucose-responsive behavior.^72^

### 4.2 pH-Responsive Basic Polymers

The pH-responsive polymers are the weak polybases that go through deionization/ionization transitions between pH 7 and 11. Groups of amines are present inside the chain of the structure. Proton groups are released at low pH, and the release of polyelectrolytes occurs under mild conditions. Polymers based on (meth)­acrylates, vinylic monomers, and (meth)­acrylamides that incorporate weakly basic functionalities, such as piperazine, tertiary amines, pyrrolidine, imidazole, morpholine, and pyridine groups, are widely used as pH-responsive materials. Among these, polymers containing tertiary amine functionalities are particularly prominent due to their reversible protonation behavior under acidic conditions. Such ionizable groups confer pH-dependent solubility, swelling, and charge characteristics, making (meth)­acrylate-, (meth)­acrylamide-, and vinyl-based tertiary amine polymers attractive for applications in drug delivery, gene delivery, and stimuli-responsive microgels.^73,74^ Poly­[(2-dimethylaminoethyl) methacrylate], poly­[(2-diisopropylamine) ethyl methacrylate], and poly­[(2-diethylamino) ethyl methacrylate] are tertiary amine methacrylate-based polymers that are widely used in basic polymers. Poly­[(2-dimethylaminoethyl) methacrylate] (PDMAEMA) is a weakly basic polymer that exhibits pronounced pH-responsive behavior. The presence of tertiary amine groups in its side chains enables reversible protonation and deprotonation in response to environmental pH changes, resulting in tunable solubility, swelling, and charge properties in aqueous media. Poly­(2-vinylpyridine) and poly­(4-vinylpyridine) are both frequently used polymers in Poly­(vinylpyridine). Due to the deprotonation of pyridine groups, these polymers show a phase change at pH 5.^75^ The polymers having functional groups like piperazine, morpholino, imidazole, and pyrrolidine are other pH-responsive polymers. The morpholino groups are present in the Poly­[(2-*N*-morpholino) ethyl methacrylate], which responds to ionic strength. Both Armes and Butun Groups have demonstrated the synthesis and studies of the solution activity of different Poly­(2-*N*-morpholino ethyl) methacrylate depending on the polymers.^76^ It must also be observed that dendrimers may be categorized under pH-response polymers like poly­(propyleneimine), poly­(amidoamine), and poly­(ethylenimine) and grafted with different polymers, and changed with several functionalities.^77,78^ In Figure 5, basic monomers used for synthesizing pH responsive-*co*-polymers are represented.

**Figure 5:**
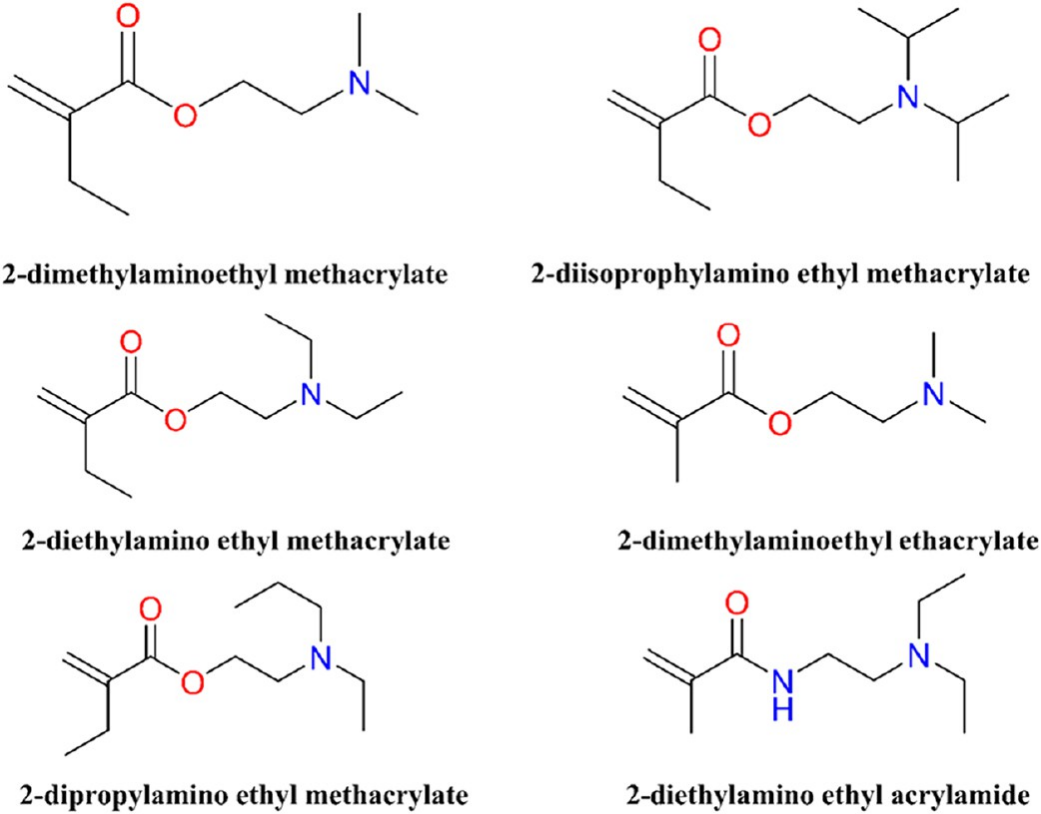
Basic monomers used for synthesizing pH responsive-*co*-polymers. This figure presents commonly employed basic monomers that serve as fundamental building blocks for the synthesis of pH-responsive copolymers. The structures were prepared using ChemDraw Professional 16.0.

### 4.3 pH-Responsive Natural Polymers

A review of the literature indicates that pH-responsive natural polymers have been extensively investigated for a wide range of biomedical applications.^79,80^ Moreover, natural biodegradable polymers are commonly used in the pharmaceutical industry because they are easily modified and have high biocompatibility via a simple method. pH-responsive natural polymers are used in the treatment of acute or chronic wound healing processes, tissue, and bone regeneration.^81^ Commonly used natural polymers include alginic acid, gelatin, dextran, chitosan, and hyaluronic acid. Owing to their inherent biocompatibility, biodegradability, and low toxicity, these polymers are widely investigated for biomedical applications. Through appropriate chemical modification, such as grafting, cross-linking, or functionalization with pH-responsive moieties, these natural polymers can be tailored into effective carriers for targeted drug-delivery systems, enabling controlled release and site-specific therapeutic action. In a few studies, grafting of pH-responsive polymers on a polysaccharide backbone has been studied. In hydrogel preparation, the use of pH-responsive natural polymers as a cross-linking agent shows good results in drug delivery applications. Chitosan is commonly used or studied as a pH-responsive natural polymer.^26,27^ In Figure 6, a schematic representation of pH-responsive natural polymers is shown.^82,83^

**Figure 6:**
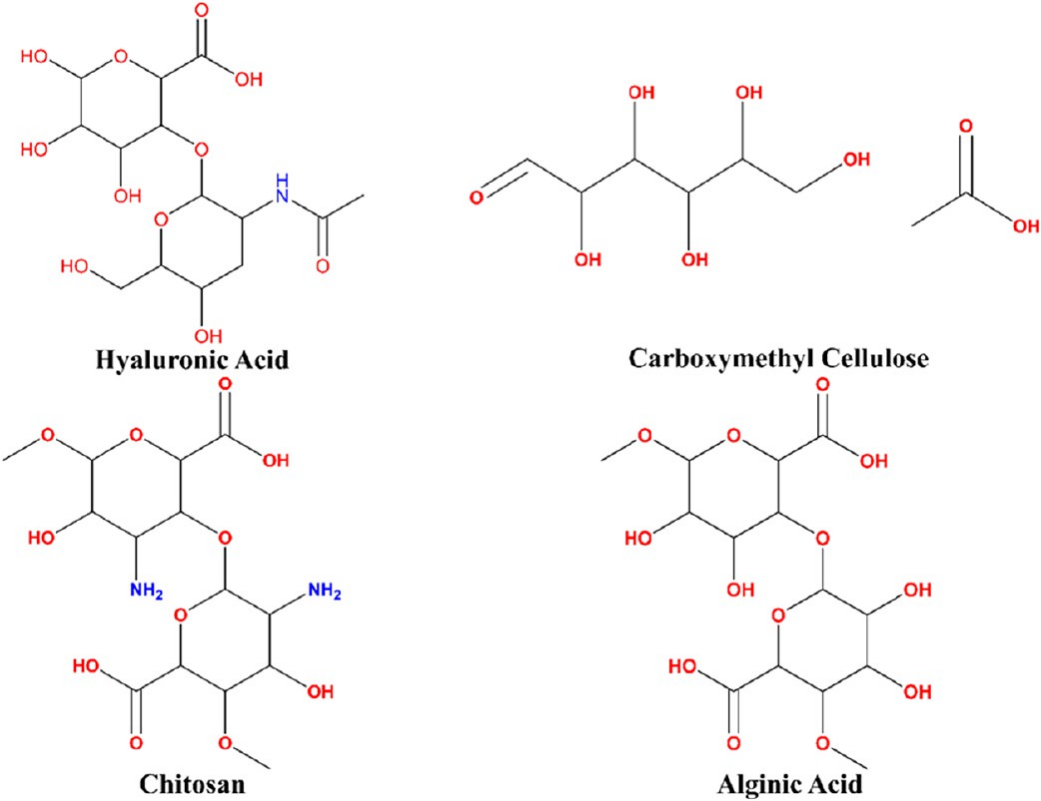
pH-Responsive Natural Polymers. This figure illustrates representative natural polymers that exhibit pH-dependent behavior, making them highly suitable for biomedical applications. The structures were prepared using ChemDraw Professional 16.0.

## 5 Synthesis of pH-Responsive Polymers

Researchers have reported several polymerization strategies for the synthesis of pH-responsive polymers, including emulsion polymerization (mini- and microemulsion),^55^ group transfer polymerization (GTP),^113^ reversible addition–fragmentation chain transfer (RAFT) polymerization,^114^ and other controlled/living radical polymerization techniques such as atom transfer radical polymerization (ATRP).^115^ These methods enable the preparation of polymers with diverse molecular architectures, including block, graft, gradient, branched, random, and polymer brush structures. Among these approaches, free-radical polymerization remains the most widely employed technique due to its simplicity, robustness, broad monomer compatibility, and suitability for large-scale production, despite offering comparatively less control over polymer architecture than controlled/living methods.^116,117^

### 5.1 Emulsion Polymerization

Vinyl-based, pH-responsive polymers can be synthesized as microgels via emulsion polymerization. This approach relies on free-radical chain polymerization to produce latex particles with fine and controlled particle sizes. The polymerization system typically consists of monomers dispersed in an aqueous medium, along with an initiator and surfactant. Colloidal stabilization of the growing polymer particles is achieved through steric or electrostatic mechanisms. In some formulations, solid particles may be introduced at the beginning or toward the end of the polymerization, particularly during the phase separation stage, to tailor emulsion characteristics and microgel structure. Despite its versatility and scalability, a major limitation of emulsion polymerization is the difficulty associated with the complete removal of residual surfactants, which may adversely affect the purity, performance, and biocompatibility of the resulting microgels. Eliminating surfactant can be achieved through flocculation or coagulation of latex via desorption or dialysis.^118^ In addition, preparation of a hybrid core–shell composite inorganic core is prepared by the emulsion polymerization method. After cross-linking of the water-swollen layer shell, with etching the metallic core could be prepared as hollow nanocages.^119^ Emulsion polymerization techniques are developed in a heterogeneous medium. There are a few criteria that need to be met for techniques to happen in a hetero phase reaction. Initially, the monomer (organic phase) needs to be completely insoluble in water (aqueous phase). The process of nucleation of monomer-swollen micelles is allowed to occur if ionic surfactant (anionic, cationic, or amphoteric) amounts are more than the critical micelle concentration, resulting in the development of micelles in the aqueous phase and formation of new polymer particles.^120^ In the literature, the mechanism of homogeneous nucleation is commonly illustrated schematically (Figure 7a and b) to aid conceptual understanding. In this mechanism, the surfactant concentration in the formulation is maintained below the critical micelle concentration (CMC); consequently, micelle formation does not occur. Under these conditions, polymer particle nucleation proceeds through the homogeneous nucleation pathway, wherein oligomeric radicals formed in the aqueous phase grow until they reach a critical chain length, become insoluble, and precipitate to form primary polymer particles. In Figure 7a, at the initial prepolymerization stage, the system comprises two main phases. The continuous aqueous phase contains monomer and initiator dissolved up to their saturation limits, together with molecularly dispersed surfactant (at or below the CMC); no polymer chains are present, and mass transfer is governed by solubility equilibria between the aqueous phase and monomer droplets. Excess monomer exists as discrete droplets stabilized by surfactant adsorption at the oil–water interface, which lowers interfacial tension and prevents coalescence. At this stage, polymerization has not yet initiated, no surface-active oligomeric species are formed, and stabilization is provided solely by low-molecular-weight surfactants, corresponding to a kinetically inert prenucleation regime dominated by emulsification rather than particle formation. In Figure 7b, upon initiator decomposition, primary radicals form in the aqueous phase and initiate polymerization with dissolved monomer, generating short, initially hydrophilic oligomeric radicals. As chain growth proceeds, increasing hydrophobicity renders these oligomers amphiphilic beyond a critical length, enabling them to act as *in situ*–generated, surface-active species. These amphiphilic oligomeric radicals rapidly adsorb at interfaces or self-assemble within the aqueous phase, triggering particle nucleation. Consequently, stabilization transitions from being dominated by free surfactant molecules to being governed by polymeric species, with oligomeric radicals serving both as reactive intermediates and colloidal stabilizers that promote the formation of stable polymer particles. The degree of polymerization for surface activity is sometimes referred to as z, and the oligomer as a z-mer.^121,122^ This mechanism underpins many modern emulsion and dispersion polymerization strategies, including surfactant-assisted and surfactant-free systems, and explains how polymer chains can assume a dual role as structural building blocks and dynamic stabilizing agents during polymer formation. As illustrated in Figure 7c, once the growing oligomeric radical reaches a critical chain length (z-mer), it rapidly enters a monomer-swollen micelle, where further propagation occurs. Figure 7d shows that nascent polymer particle nuclei subsequently compete with monomer-swollen micelles for the capture of these oligomeric radicals, thereby governing the dominant nucleation pathway and early particle growth.

**Figure 7:**
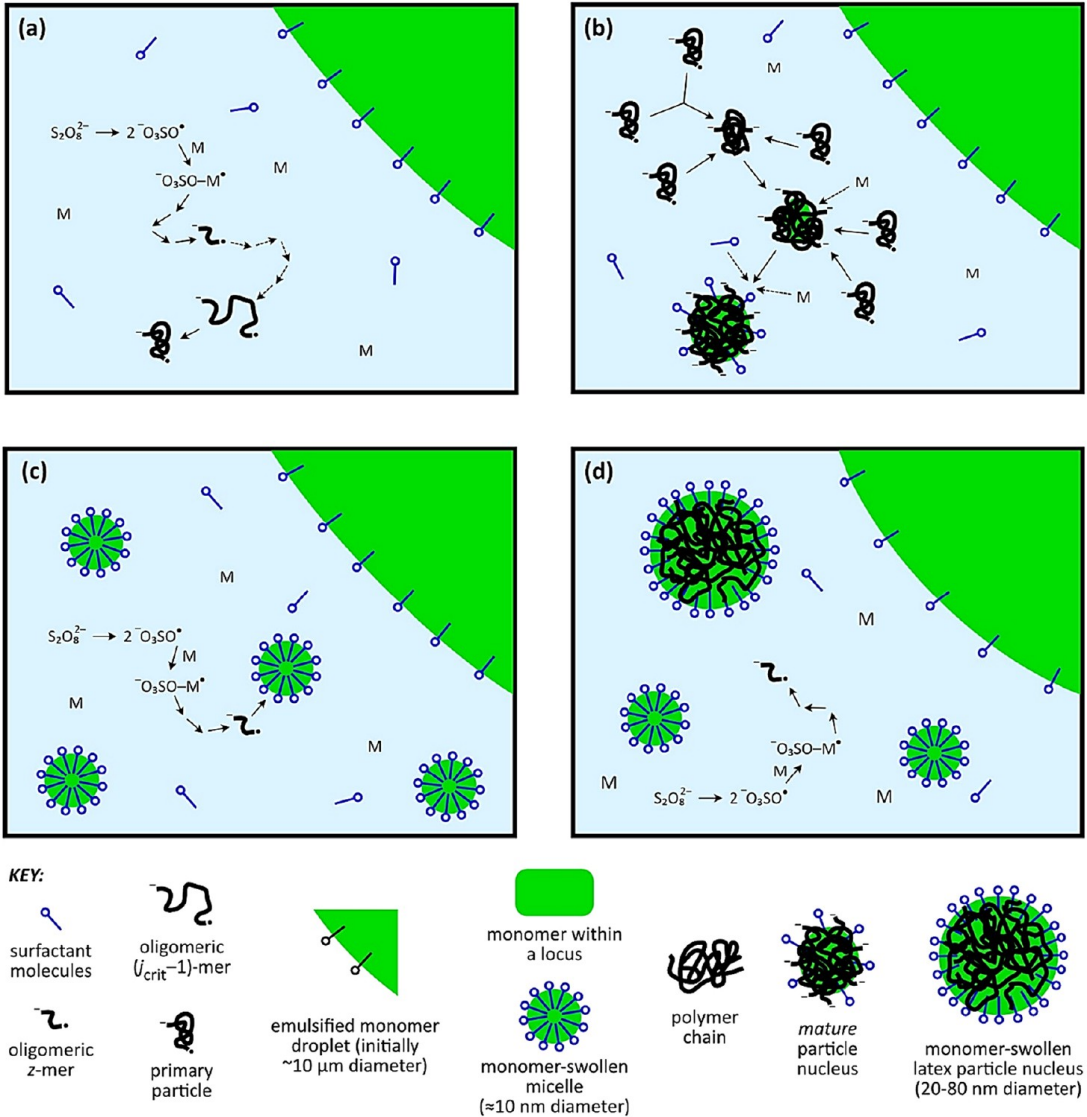
Schematic diagram showing scenarios for nucleation in emulsion polymerization. Homogeneous nucleation is represented in (a) and (b), which show the phases existing at the onset of polymerization and limited coagulation of primary particles, respectively, with surfactant at a concentration below the CMC. Micellar nucleation is represented in (c) and (d), which show the phases existing at the onset of polymerization and partway through nucleation, respectively, with surfactant at a concentration above the CMC. Note that homogeneous nucleation can also occur when the surfactant concentration is above the CMC. Dashed lines for arrows indicate multiple processes of the same kind. Adapted with permission from Open Access.^123^

### 5.2 Mini-Emulsion Polymerization

Mini-emulsion polymerization typically involves an initiator, monomer(s), water, and a surfactant as the primary components. The key distinctions between conventional emulsion polymerization and mini-/microemulsion polymerization are the use of low-molecular-weight costabilizers (such as hydrophobes) and the application of high-shear energy, commonly introduced through ultrasonication or high-pressure homogenization. In mini-emulsion systems, the interfacial tension between the monomer droplets and the aqueous phase remains greater than zero; therefore, continuous high shear is required to generate and maintain kinetically stable nanodroplets. This contrasts with microemulsion systems, which are thermodynamically stable and exhibit ultralow or effectively zero interfacial tension. As a result, mini-emulsion polymerization relies on mechanical energy to achieve a steady dispersion state, with polymerization occurring predominantly within the stabilized monomer droplets.^124^ In the study conducted by Kriwet et al., poly­(acrylic acid) nanoparticles were synthesized using a mixture of Tween 80 and Span 80 as a coemulsifier.^125^ Particle size and free radicals depended on the radical initiator in polymerization. Ammonium persulfate was used as a water-soluble initiator. The diameters of nanoparticles were 80 and 150 nm.^126^ Ugelstad, Vanderhoff, and El-Aasser first demonstrated mini-emulsion polymerization in 1973, using an intense costabilizer/surfactant system to produce small monomer droplets (0.08–0.30 μm).^127–129^ In these situations, the droplet interfacial area was huge, and surfactant was adsorbed at the droplet surfaces with surfactant concentration in the free aqueous phase under the CMC, so no micelle formation occured. Ideally, water-soluble initiators like persulfates are used in emulsion polymerizations.^130^ Figure 8 schematically illustrates the nucleation mechanism in mini-emulsion polymerization, highlighting the formation and stabilization of polymer particles originating from monomer droplets under kinetically controlled conditions and contrasted with Figure 7. Additionally, it needs to be highlighted that the mini-emulsion polymerization technique is much simpler when these two figures are compared.

**Figure 8:**
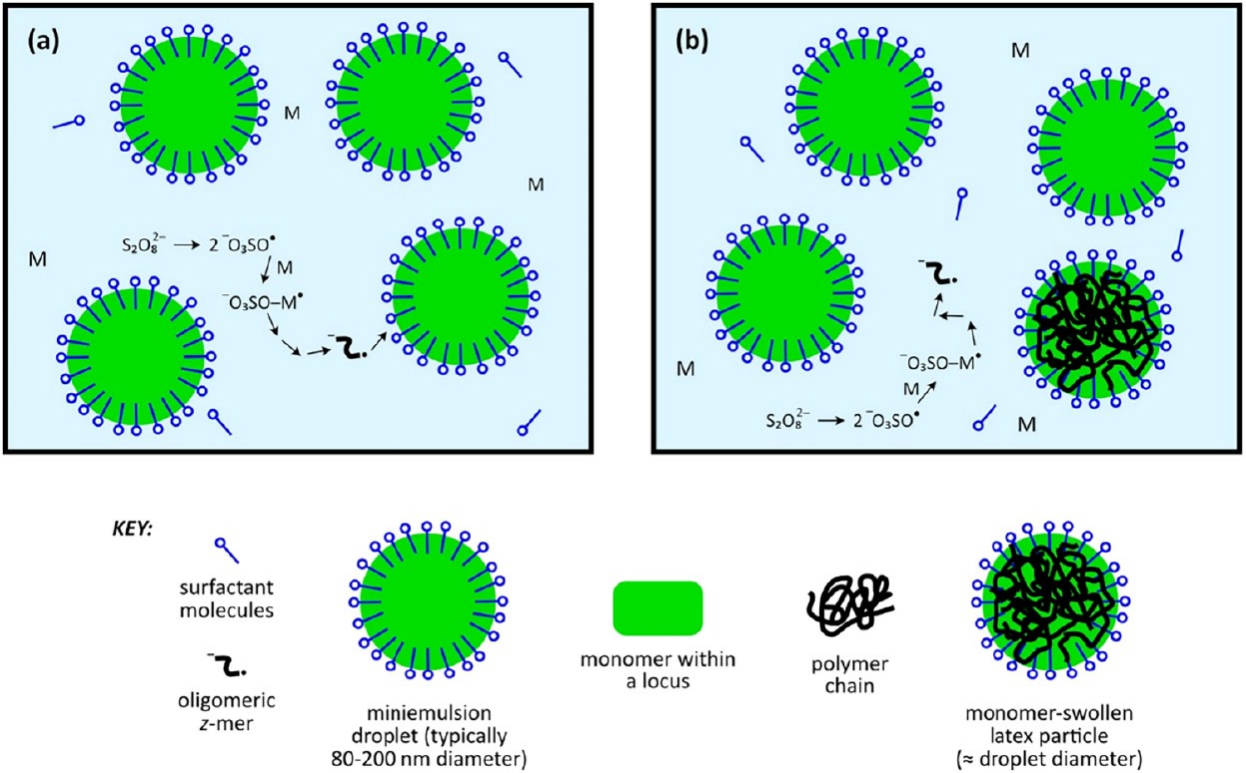
Schematic representation of the nucleation mechanism in miniemulsion polymerization illustrating the initial stage of polymerization, characterized by the absence of micelles and a low concentration of molecularly dissolved surfactant (a); and the intermediate stage of nucleation, showing the evolution of distinct phases during particle formation (b). Adapted with permission from Open Access.^123^

### 5.3 Microemulsion Polymerization

Microemulsion polymerization is a novel and efficient method for the development of nanosized polymer particles. Microemulsion and emulsion polymerization differ in the kinetics of the polymerization. The emulsion polymerization has three reaction rate intervals, but two are identified in microemulsion polymerization. Microemulsion polymerization significantly reduces both the average and the size of chains per particle.^131^ A thermodynamically stable microemulsion with swelling micelles has an initiator, which is normally water-soluble, used in the aqueous phase in microemulsion polymerization. The polymerization proceeds in a thermodynamically stable, naturally developed state and depends on large amounts of surfactant complexes, which have interfacial tensions that are near zero at the oil/water contact interface. Moreover, the particles are totally covered by surfactant because a larger amount of surfactant is utilized. Generally, the numerous applications of polymer latex developed by microemulsion polymerization; however, the common polymer formulations are diluted and need a high surfactant-to-monomer ratio, so the practical application of this technique has been limited. For polymer stability purposes, the surfactant concentrations generally need to be in high amounts. Figure 9 summarizes the most important properties of microemulsion and mini-emulsion.^132^

**Figure 9:**
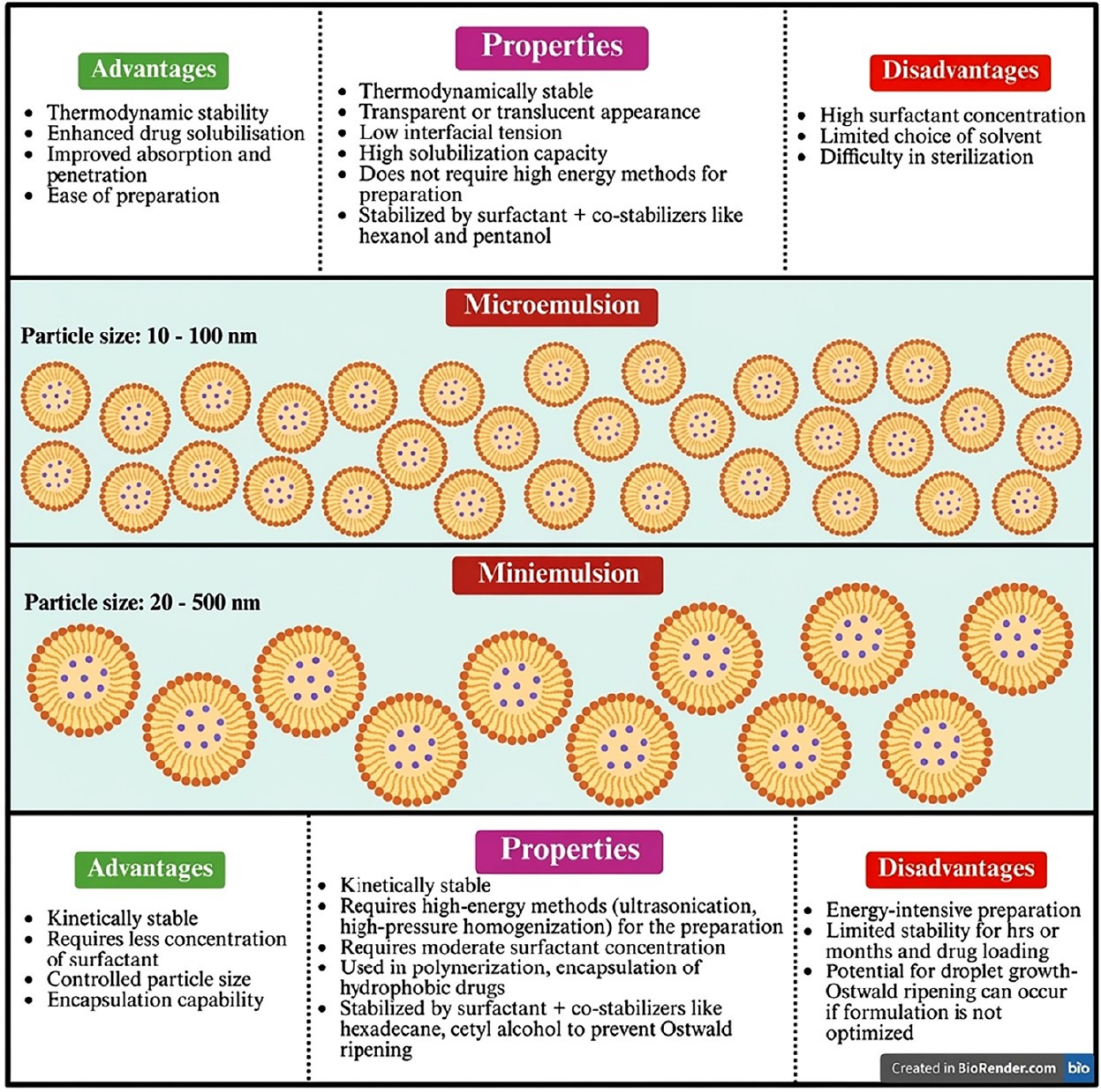
Schematic illustration depicting comparison of microemulsions and miniemulsion in terms of particle size, properties, advantages, and disadvantages. This image has been created as a Creative Common using the BioRender software (https://www.biorender.com/).

### 5.4 Group Transfer Polymerization and Atom Transfer Radical Polymerization (ATRP)

In the year 1983, Webster et al. developed group transfer polymerization (GTP), also known as the living polymerization technique.^133^ Controlled living polymerization methods are being developed for polymers that have narrow molecular weight distributions. These techniques involved radical and ionic chemistry. GTP accepts the living polymerization of (meth)­acrylates at ambient temperature. These techniques have some limitations; GTP is not acceptable for all monomers, and due to the reaction conditions. Despite requiring relatively demanding reaction conditions, pH-responsive polymers based on tertiary amine methacrylates, such as 2-(dimethylamino)­ethyl methacrylate (DMAEMA), 2-(diethylamino)­ethyl methacrylate (DEAEMA), and related monomers, have been successfully synthesized using group transfer polymerization (GTP). This technique enables precise control over polymer architecture, allowing the formation of well-defined branched, block, and star-shaped polymer structures.^134–137^ Methacrylic acid, as a functional monomer, cannot be used in GTP. In many polymerization strategies, functional monomers are temporarily masked with protecting groups that can be readily converted back to the desired functional species after polymerization. The use of living/controlled radical polymerization techniques enables polymer synthesis under relatively mild reaction conditions while providing precise control over molecular weight and a narrow molecular weight distribution. Moreover, these techniques allow the preparation of polymers with well-defined architectures, such as block, graft, star, or branched structures, along with tailored end-group functionalities. However, GTP describes the polymerization of acrylic monomers, such as methyl methacrylate (MMA), initiated by a silyl ketene acetal (SKA) in the presence of a nucleophilic or Lewis acid catalyst. The term originally reflected an associative propagation mechanism, in which the silyl group remains bound to a single growing chain and is intramolecularly transferred to the incoming monomer via a hypervalent anionic silicon intermediate (path a, Scheme 1). More recent studies, however, provide compelling experimental evidence for a dissociative mechanism, wherein ester enolate anions act as the true propagating species, undergoing rapid and reversible complexation (termination) with SKA or its polymeric analogue (path b) (Figure 10).^138^ There are a few examples of pH-responsive polymers with several architectures described with the nitroxide-mediated radical polymerization (NMP) technique, such as PDMA-ra-PVBK, PPO-*b*-P­(DMA-*stat*-2VP), PAAc-grad-PS and P2VP-*b*-PNIPAAm.^139,140^

**Figure 10:**
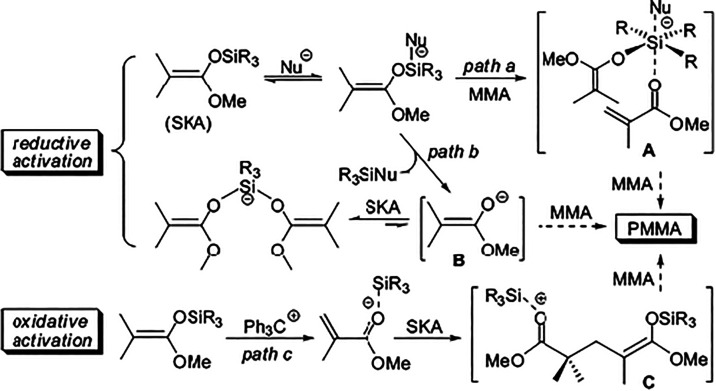
Schematic representation of the proposed mechanistic pathways for MMA polymerization initiated by silyl ketene acetals, highlighting associative intramolecular silyl transfer, dissociative propagation via ester enolate anions, and bimolecular interactions contributing to chain growth or termination. Reprinted with permission from ref 138. Copyright 2008, American Chemical Society.

In 1995, Matyjaszewski, Sawamoto, and their respective co-workers independently developed ATRP as a controlled/living radical polymerization (CRP) technique.^141^ CRP technique is acceptable for a broad range of monomers, including (meth)­acrylamides, styrenes, and (meth)­acrylates. However, it has a few drawbacks in the polymerization of acrylamide and derivatives. A wide variety of multi- and monofunctionalized ATPR initiators can be easily developed and obtained commercially. Moreover, polymers synthesized by ATRP use a macroinitiator for the initial steps, which is a primary advantage of the ATRP technique. pH-responsive polymers are easily prepared with several architectures, such as star, brushes, branched, gradient and block copolymers, and the technique is commonly used to synthesize pH-responsive polymers.^142–144^ Alkyl halides are used as initiators in ATRP, and numerous ligands and transition metals are well-immersed as catalysts. Mainly, the catalyst, dependent on N-containing ligands and copper, was used. The limitation of ATRP is the use of a greater number of catalysts. This pH-responsive polymer application is a smart material for adhesion, separation, tissue engineering and drug delivery systems.^145,146^ Figure 11 represents the synthetic route for the preparation of an ampholytic block copolymer, poly­(methacrylic acid)-*block*-poly­(2-(diethylamino)­ethyl methacrylate) (PMAA-*b*-PDEA), synthesized via atom transfer radical polymerization (ATRP).

**Figure 11:**
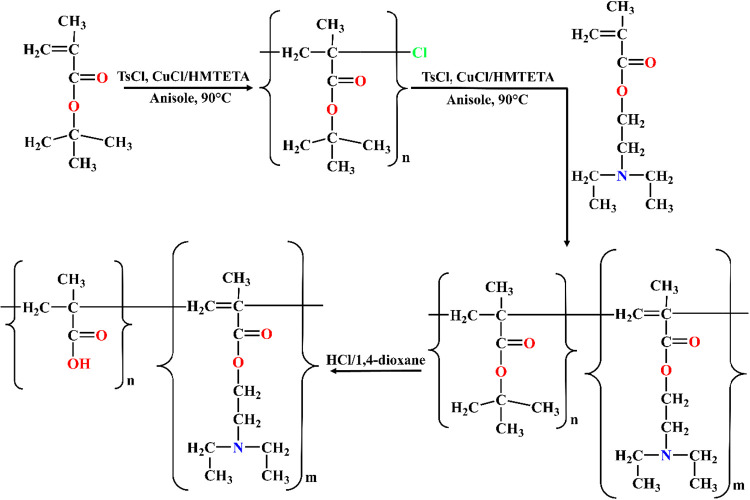
This figure illustrates the stepwise synthesis of an ampholytic block copolymer composed of poly­(methacrylic acid) (PMAA) and poly­(2-(diethylamino)­ethyl methacrylate) (PDEA) using Atom Transfer Radical Polymerization (ATRP). The structures were prepared using ChemDraw Professional 16.0.

### 5.5 Reversible Addition–Fragmentation Chain Transfer (RAFT) Polymerization

Reversible addition–fragmentation chain transfer (RAFT) polymerization is one of the continuous and commonly used techniques for synthesizing branches, blocks, brushes, and stars over the last few decades.^147^ The incorporation of a chain transfer agent (RAFT-CTA) into the polymerization medium fundamentally distinguishes reversible addition–fragmentation chain transfer (RAFT) polymerization from conventional free-radical polymerization, as it enables reversible chain growth and precise control over molecular weight and polymer architecture. RAFT polymerization technique is applicable for styrenics, (meth)­acrylamides, and (meth)­acrylates, although RAFT reagents are not suitable for secondary and primary amines.^148^ In the ATRP technique, a few polymers are gained from the RAFT technique and used as a macro-RAFT agent. Moreover, the polymers gained from RAFT and ATRP techniques have multi- or monofunctional end groups; additionally, these groups of polymers can be developed to synthesize multiple polymeric materials. For example, Armes’ group has developed nonionic PG2MA-*b*-PHPMA diblock copolymers, which show a pH-responsive nature.^149^ Figure 12 represents the mechanism of the basic equilibria process involved during RAFT polymerization.^150^

**Figure 12:**
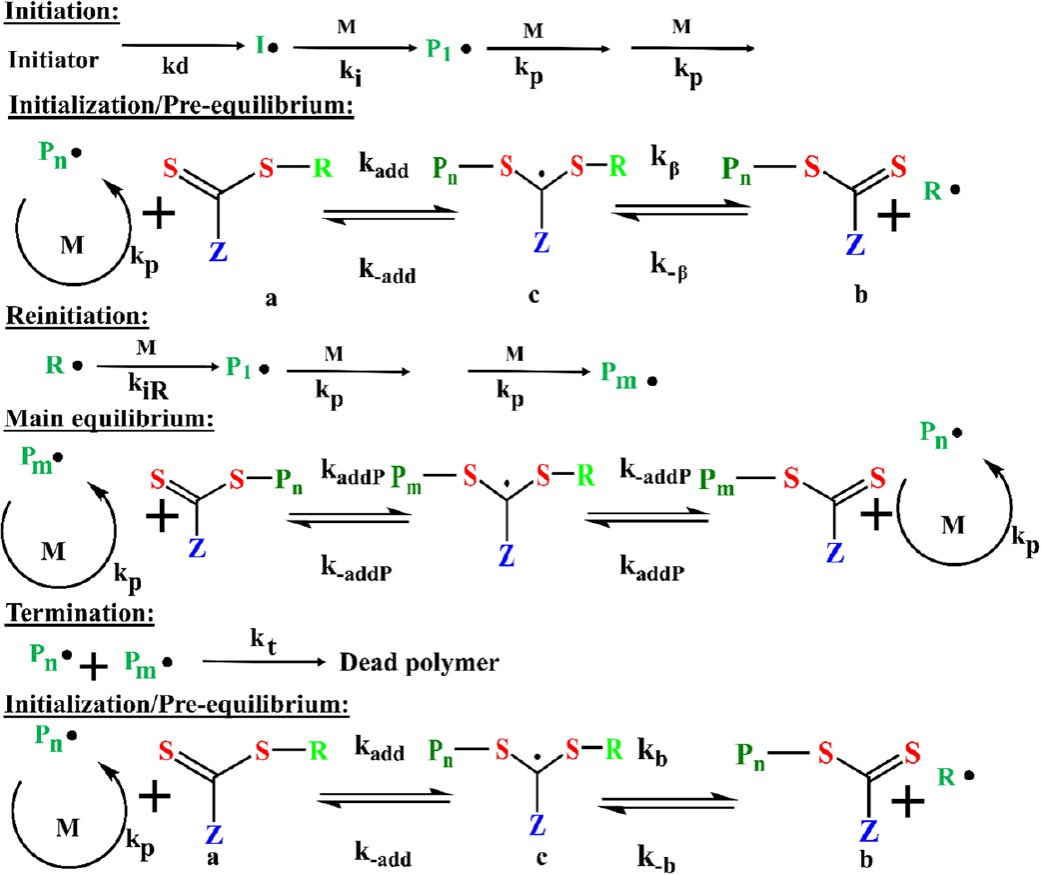
Illustration of the fundamental equilibria governing Reversible Addition–Fragmentation Chain Transfer (RAFT) polymerization, a controlled radical polymerization technique. The structures were prepared using ChemDraw Professional 16.0.

The thiocarbonylthio end group introduced during RAFT polymerization can be quantitatively converted into a free thiol via aminolysis or reduction, providing a chemically versatile handle for site-specific bioconjugation with proteins and peptides.^151,152^ This thiol chain end enables efficient conjugation through thiol–maleimide coupling, thiol–ene reactions, or reversible disulfide formation, allowing precise control over polymer–protein stoichiometry while preserving protein structure and activity.^153^ When combined with pH-responsive polymer backbones, thiol-terminated RAFT polymers become particularly attractive for intracellular protein delivery. Such polymers are typically engineered to remain relatively inert at physiological pH but undergo protonation-driven conformational changes under acidic conditions encountered in endosomes and lysosomes.^154,155^ This shift can induce membrane-active behavior, facilitating endosomal escape and cytosolic release of the conjugated protein cargo. The modularity of RAFT synthesis allows precise incorporation of ionizable or hydrophobic comonomers that tune pH sensitivity and membrane interaction.

A representative case study by Duvall et al. demonstrated the conjugation of a pro-apoptotic peptide to a RAFT-synthesized diblock copolymer via thiol–disulfide exchange at the polymer chain end.^156^ The second block, designed to be pH responsive, transitioned from membrane-inert to membrane-disruptive behavior at endosomal pH, resulting in efficient endosomal escape, cytosolic peptide release, and significantly enhanced apoptotic activity in cancer cells compared to the free peptide. This work highlighted the dual role of RAFT thiol chemistry in enabling both site-specific bioconjugation and stimulus-responsive intracellular trafficking. In another important example, Sumerlin and co-workers conjugated RAFT-derived polymers to proteins using sequential thiol–ene reactions, demonstrating that polymer responsiveness (initially thermoresponsive, but extendable to pH-responsive systems) could be transferred to the protein conjugate without compromising protein integrity.^157^ More recent developments have extended this concept to self-immolative RAFT end-group modifications, enabling reversible protein attachment and triggered release under physiologically relevant conditions, further enhancing the adaptability of polymer–protein conjugates for delivery applications.^158^

Liu and his co-workers reported a well-defined heterotelechelic metal-chelating polyacrylamide polymer (DP_n_ ≈ 50, PDI ≈ 1.2) synthesized by RAFT polymerization and bearing terminal biotin with or without doxorubicin (Dox) and multiple DTPA pendant groups, which were conjugated via streptavidin to trastuzumab Fab fragments for targeting HER2-overexpressing SK-BR-3 breast cancer cells.^151^ Surface plasmon resonance showed that polymer attachment and Dox functionalization did not compromise HER2 binding (low-nanomolar K_D). Confocal microscopy and quantitative ∧111In cell-fractionation revealed efficient HER2-specific internalization, with polymer-containing conjugates exhibiting markedly enhanced intracellular, nuclear, and chromatin localization compared with Fab controls. The polymer lacking terminal Dox achieved the highest nuclear (∼32%) and chromatin (∼24%) accumulation, while Dox incorporation slightly reduced nuclear uptake, likely due to altered intracellular trafficking. Charge effects were critical: partial neutralization of polymer carboxylates by indium saturation reduced nuclear localization, implicating polymer charge and endosomal escape as key drivers of intracellular routing. Overall, the data demonstrate that RAFT-derived metal-chelating polymers intrinsically promote cellular internalization, endosomal escape, and nuclear delivery of trastuzumab Fab fragments in HER2-positive cells, underscoring their promise as multifunctional carriers for Auger electron radioimmunotherapy, while highlighting the need to optimize polymer charge and end-group chemistry to maximize nuclear targeting efficiency. In a separate set of experiments, polyacrylamide-based metal-chelating polymers (MCPs) of similar chain length (DP_n_ ≈ 36–40, *Đ* ≈ 1.2), synthesized by RAFT polymerization and end-functionalized with biotin and multiple DTPA chelators, were complexed via streptavidin to trastuzumab Fab fragments to assess the impact of polymer charge architecture on immunoreactivity and *in vivo* biodistribution.^152^ A polyanionic MCP bearing ethylenediamine-DTPA pendant groups was directly compared with a zwitterionic MCP containing diethylenetriamine-DTPA pendant groups that become charge-neutral upon indium saturation. Surface plasmon resonance showed that both conjugates retained high HER2 binding affinity (low-nanomolar K_D), comparable to native Fab. While *in vitro* studies with HER2-positive SKOV-3 cells indicated partial nonspecific binding for both polymer conjugates, *in vivo* biodistribution and microSPECT/CT imaging revealed pronounced charge-dependent effects: the polyanionic MCP conjugate exhibited rapid blood clearance and very high liver uptake (∼53% ID/g at 24 h), whereas the zwitterionic MCP conjugate showed ∼4-fold higher blood levels, ∼4-fold lower liver uptake (∼14% ID/g), reduced kidney accumulation, and significantly enhanced tumor uptake in SKOV-3 xenografts (2.8 vs 1.9% ID/g at 48 h). Overall, these results demonstrate that zwitterionic MCPs substantially improve pharmacokinetics and tumor targeting of trastuzumab-based radioimmunoconjugates by minimizing nonspecific organ sequestration, underscoring polymer charge neutralization as a critical design principle for effective Auger electron radioimmunotherapy.

Collectively, these studies demonstrate that thiol-terminated RAFT polymers serve as highly customizable precursors for protein conjugation, while pH-responsive polymer segments actively promote cellular internalization and overcome the critical endosomal escape barrier. The combination of precise chain-end chemistry, tunable pH sensitivity, and controlled polymer architecture positions RAFT-based polymer–protein conjugates as promising candidates for advanced intracellular delivery in cancer therapy, immunotherapy, and protein-based therapeutics.

## 6 Structure–Property Relationships in pH-Responsive Polymers

pH-responsive polymers have either acidic or basic groups in their structure, which can undergo ionization with a change in pH and lead to a change in the structure of a pH-responsive polymer. The complete ionization of these acidic or basic groups is difficult due to the electrostatic forces of adjacent ionized groups. By altering the charges along the backbone of polymer or electrolyte concentrations, their physical properties, such as configuration, solubility, chain conformation, and volume of pH-responsive polymers, can be customized, resulting in electrostatic repulsions that increase the polymer’s hydrodynamic volume. Any condition, such as pH, ionic strength, and counterion type, that alters electrostatic repulsion influences the transition between a tightly coiled and an extended state.^159^ The system expands upon ionization of the polymer in a suitable solvent due to the repulsive forces between charges on the polymer chain. The dissolved chain, on the other hand, stays compact and folded if the solvent inhibits the polyelectrolyte from undergoing ionization. pH-responsive polymers alter conformation substantially in response to small changes in the pH of the biological environment.^160^ At low pH values, acidic functional groups remain predominantly in their un-ionized state, resulting in the collapse or deswelling of polyacid polymers due to reduced electrostatic repulsion. As the pH increases, deprotonation of these acidic groups occurs, leading to enhanced ionization, increased electrostatic repulsion, and consequent polymer swelling. In contrast, polybasic polymers undergo protonation under acidic conditions, becoming ionized at low pH and exhibiting pronounced swelling behavior owing to increased charge density along the polymer chains. The swelling or deswelling of the hydrogels or microgels due to a change in the pH causes changes in their surface properties.^159^

pH-responsive hydrogels are mainly polyelectrolytes with either a positive or negative charge, and they work by expanding the mesh sizes of their network due to repulsive forces produced by ionization or protonation of the constituent polymer chains.^161^ Biological activities such as biomolecule adsorption/desorption and cellular contact that take place on polymers depend on the interaction between the biological environment and pH-responsive polymers. The interfacial properties of materials can be adjusted by changing their surface physical and chemical properties to influence these interactions.^160^ One of the best options for fabricating pH-responsive nanocarriers is polymers with ionizable groups, such as amines and carboxylic acids. pH-responsive carriers can respond selectively to specific pathological stimuli associated with disease microenvironments. Alginate is a well-known natural pH-responsive polymer characterized by a high content of carboxylic acid groups. In the presence of divalent cations, such as calcium ions (Ca^2+^), alginic acid undergoes spontaneous and mild gelation through ionic cross-linking, forming a stable hydrogel network suitable for controlled and targeted drug-delivery applications.^162^ This mild gelling property is pH-dependent and can be used to encapsulate a variety of molecules inside the alginate gels. The alginates can also be modified hydrophobically. In the low pH environment of the stomach, cationic polyelectrolytes like chitosan perform well, while anionic hydrogels like PAA, PMA, and others operate well in the alkaline environment of the colon.^163,164^

In solution, copolymers self-assemble and produce aggregates of different sizes and shapes. Block copolymers show self-assembling properties that are very useful. Since the main chains and pendants of pH-responsive amphiphilic block copolymers consist of ionizable groups, their domains can be adjusted to respond to aqueous environments. Depending on their structures and pH levels, these groups can receive or donate protons in an aqueous solution to produce polyelectrolytes, weak polyacids, or weak polybases.^161^ Hydrogels are polymeric three-dimensional networks that expand in aqueous conditions but do not dissolve. Similarly, the polymeric microgels form a network-like structure due to cross-linking of the gel particles. Hydrogel and polymeric microgel absorb water and swell. When secondary forces such as ionic, hydrogen bonding, hydrophobic forces or molecular entanglements, or both hold the networks together, they are called physical or reversible gels.^165^ When they have covalently cross-linked networks, they are referred to as chemical or permanent gels.^166^ The hydrogel composed of 4β-amino podophyllotoxin and α-cyclodextrin (α-CD) exhibits a distinct gel–sol transition behavior and demonstrates pH-dependent drug release under acidic conditions.^167^

## 7 Characterization of pH-Responsive Polymers and Block Copolymers

As discussed earlier, pH-responsive polymers are a key class of stimuli-responsive materials that undergo reversible physicochemical changes in response to environmental pH variations. This behavior arises from ionizable functional groups, such as carboxylic acids, amines, imidazoles, and sulfonamides, located along the polymer backbone or side chains that reversibly undergo protonation or deprotonation within defined pH ranges. Consequently, these polymers exhibit pH-dependent changes in solubility, chain conformation, hydrodynamic size, swelling, surface charge, and intermolecular interactions. Such adaptive characteristics make pH-responsive polymers highly suitable for biomedical applications, including drug and gene delivery, biosensing, and tissue engineering, where physiological and pathological pH gradients can be selectively exploited. Incorporation of pH-responsive segments into block copolymer architectures further enhances material functionality. Amphiphilic block copolymers comprising hydrophilic and pH-sensitive or hydrophobic domains can self-assemble into well-defined nanostructures, such as micelles and vesicles, in aqueous environments. The stability and morphology of these assemblies are dictated by pH-dependent ionization of the responsive block, enabling controlled assembly and disassembly under targeted conditions. Accordingly, comprehensive physicochemical characterization is essential to elucidate structure–property relationships and ensure reproducible performance in complex biological systems.

Gel permeation chromatography (GPC) is used to determine the molecular weight and polydispersity index (PDI) of the polymer.^168^ pH-responsive polymers change their conformation with a change in the surrounding pH. These conformational changes are studied using nuclear magnetic resonance (NMR) spectroscopy and dynamic light scattering (DLS). Block copolymers that are pH-responsive blocks should be analyzed using ^1^H NMR spectroscopy.^169,170^ Multiangle light Scattering (MALS), along with a Quasi-Elastic Light Scattering (QELS) detector, is used to perform static and DLS experiments. Filtered toluene is used to calibrate the absolute values of the scattered intensities.^168^ Transmission electron microscopy (TEM) is used to study the morphology of polymers, while the X-ray scattering method is used to ascertain the spatial arrangements of all atoms in polymers. X-rays are focused on the polymer, resulting in two types of scattering. Coherent scattering (X-ray diffraction) occurs for a crystalline sample; it can be determined by wide-angle measurements and called as wide-angle X-ray scattering (WAXS), while incoherent scattering (diffuse diffraction) occurs for a sample with nonhomogeneous or semicrystalline morphology; it can be determined by small-angle measurements - small-angle X-ray scattering (SAXS). For this, samples are made by dissolving the copolymer in aqueous solutions with the required ionic and pH conditions. The real space imaging of nanometre-sized structures can be done using TEM.^168^ Fourier transform infrared (FTIR) spectroscopy can be used to study the interaction between the pH-responsive polymer and the drug, and to identify the functional groups present on the polymer. Particle size distribution is determined using DLS. Potentiometric titrations are performed to determine the pH value at which the functional group present on the polymer becomes ionised or un-ionized and changes its conformation; pH meters are used to perform potentiometric titrations.^168,171^

Various amphiphilic block copolymers were used to design a responsive polymer that changes its conformation with a change in pH. Monomers that were used to synthesize the block copolymer were 4-(1H-imidazol-1-yl) butyl-methacrylate hydrochloride (ImBuMA), 6-(1H-imidazol-1-yl) hexyl-methacrylate hydrochloride (ImHeMA). The individual blocks are made up of acrylate polymers that have weak basic side chains and permanent hydrophilic chains with neutral functionality. The conversion of monomer to polymer was identified by ^1^H NMR. Variations in molar mass, comonomer content, and block ratios or compositions give rise to distinct pH-responsive behaviors, which are often manifested through reversible self-assembly into micelles and/or polymersomes. These pH-induced transitions can be precisely tuned to elicit controlled responses within physiologically relevant pH ranges, particularly between pH 5 and 7. At pH higher than 5.8, there is a conversion of protonated imidazole units into more hydrophobic free-base imidazole moieties, which results in a self-assembled structure. This idea was supported by DLS of NMR samples, which revealed that increasing the pH of the polymer solution from 3 to 5.6 caused unimeric polymer chains to aggregate. This resulted in the formation of particles over 1 μm in size at pH 6.5–6.8. Under a more basic environment, the size shrank dramatically, reaching a constant size of around 70 nm at pH ≥ 8.0. Turbidimetric analysis was also carried out, and the pH at which the polymer aggregated, as shown by turbidimetric analysis, was matched with the results of DLS and ^1^H NMR. The critical aggregation concentration (CAC) of the copolymer was estimated using a spectrofluorometer. TEM analysis revealed that polymersomes having a diameter in the range of 100–200 nm can be produced in the pH condition where the weakly charged side-chains are deprotonated.^170^

Collectively, comprehensive characterization of pH-responsive polymers and block copolymers is essential for establishing robust structure–function relationships and guiding rational material design. By integrating spectroscopic, chromatographic, colloidal, and morphological characterization techniques, researchers can achieve a holistic understanding of pH-dependent behavior. This multidimensional insight enables the systematic optimization of these smart materials for advanced biomedical applications, including targeted drug delivery, diagnostics, and responsive therapeutics (Figure 13).

**Figure 13:**
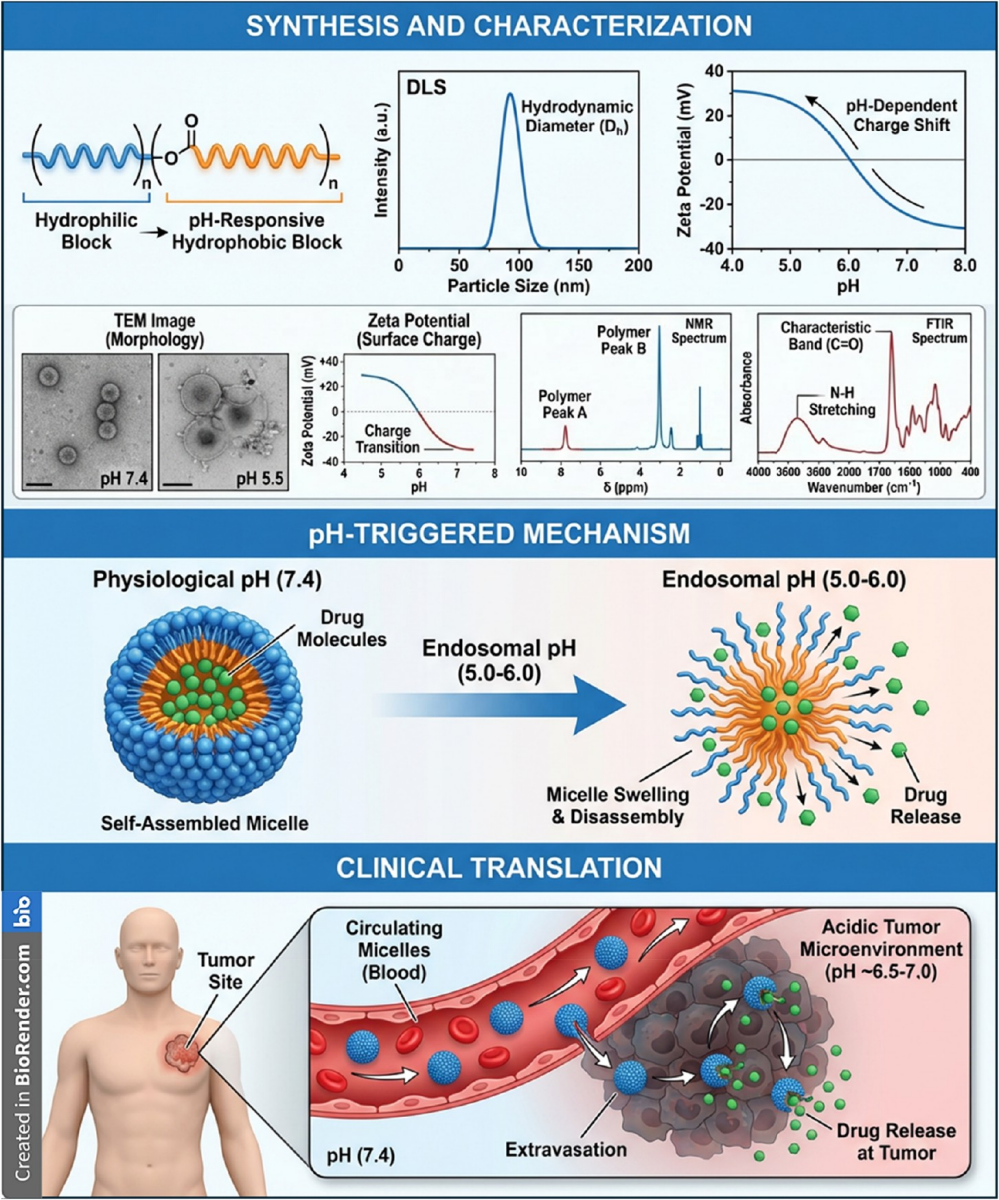
Design, characterization, and pH-triggered drug release of pH-responsive polymeric micelles. *Top:* Schematic of the amphiphilic block copolymer and physicochemical characterization, including hydrodynamic size (DLS), pH-dependent surface charge (zeta potential), morphology (TEM), and spectroscopic analyses (NMR and FTIR). *Middle:* pH-responsive mechanism showing stable self-assembled micelles at physiological pH (7.4) and micelle swelling/disassembly under acidic endosomal pH (5.0–6.0), leading to drug release. *Bottom:* Proposed *in vivo* pathway illustrating circulation stability, tumor extravasation, and selective drug release in the acidic tumor microenvironment. This image has been created as a Creative Common using the BioRender software (https://www.biorender.com/).

## 8 pH-Responsive Polymers: From Molecular Design to Clinical Applications

pH-responsive polymers have emerged as a cornerstone in the development of smart biomaterials, offering precise control over physicochemical properties in response to environmental pH changes. These adaptive systems exploit ionizable functional groups that undergo protonation or deprotonation, enabling reversible transitions in solubility, swelling, and conformation. Such behavior is particularly advantageous in biomedical contexts where pH gradients exist, including tumor microenvironments, inflamed tissues, and intracellular compartments. Recent advances have expanded their applications beyond conventional drug delivery to include gene therapy, tissue engineering, biosensing, and implantable devices, among others (Figure 14). For instance, pH-sensitive hydrogels enable localized drug release in acidic wound sites, while polymeric micelles facilitate selective delivery of anticancer agents to acidic tumor regions.^172^ Furthermore, hybrid systems integrating inorganic nanoparticles or bioactive molecules with pH-responsive polymers have demonstrated synergistic performance in diagnostics and therapeutics.^173^ Accordingly, the subsequent sections explore the diverse biomedical applications of adaptive polymers, with a particular emphasis on their role in advanced drug delivery systems.

**Figure 14:**
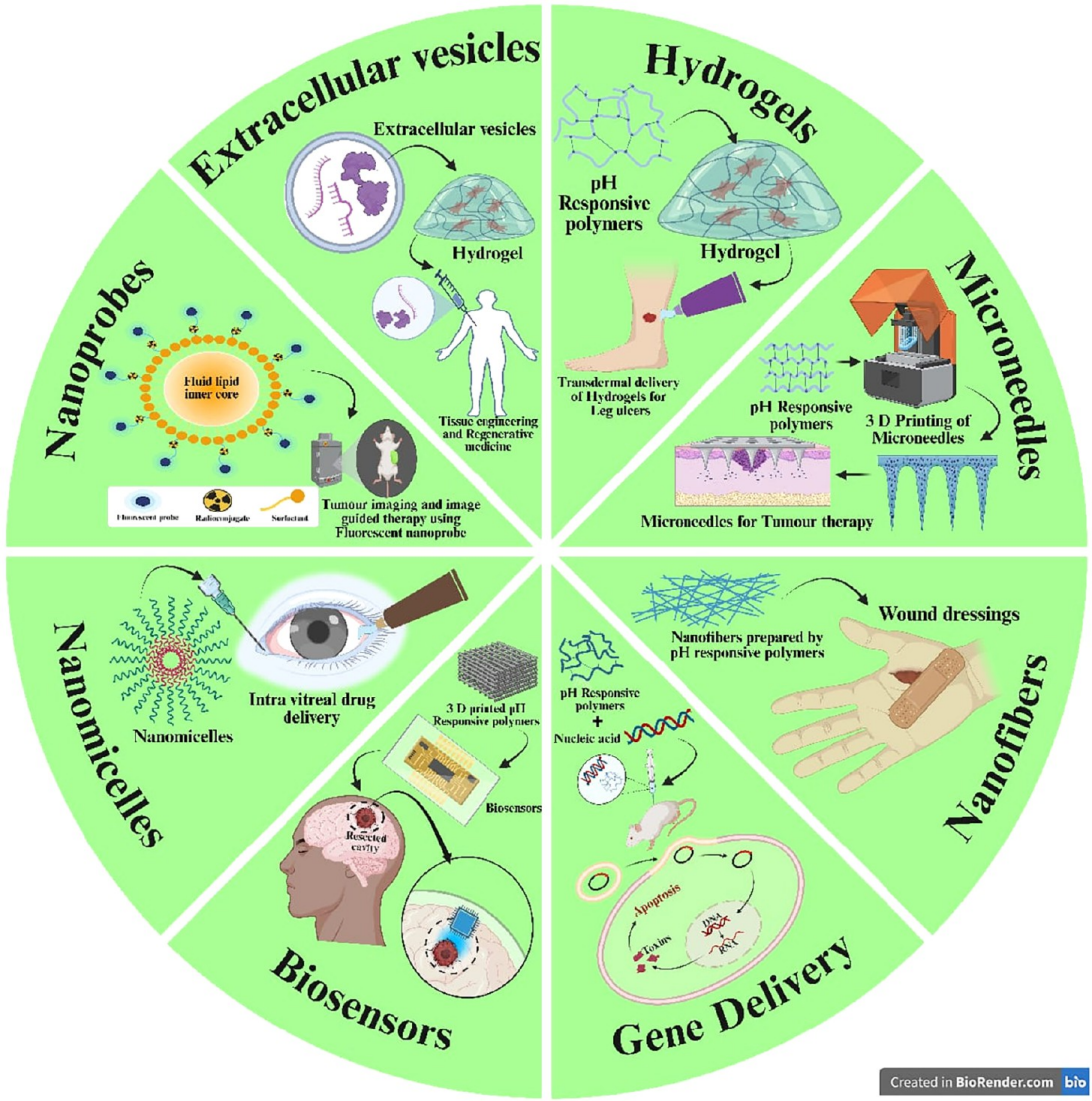
Schematic representation of advanced drug delivery platforms and biomedical applications for pH-responsive polymers, including extracellular vesicles, hydrogels, microneedles, nanofibers, gene delivery systems, biosensors, nanomicelles, and nanoprobes. This image has been created as a Creative Common using the BioRender software (https://www.biorender.com/).

### 8.1 Hydrogel

A hydrogel is a three-dimensional, cross-linked polymeric network. The different characteristics of hydrogels, such as biocompatibility, hydrophilicity, and external stimuli-sensitive nature, make them suitable for use in diverse fields. The smart and intelligent behavior of hydrogels has made them popular in biomedical applications.^174^ According to literature, hydrogel has some key advantages, including the presence of polymeric chains with different functional groups, allowing incorporation of responsiveness toward different stimuli such as heat, light, pH, and chemical agents. They are biocompatible, biodegradable, and porous, and they support a longer duration of action. However, they have certain limitations; rapid burst drug occurs during the swelling, and slow response to sensory stimuli. Various studies report the fabrication of hydrogel by various methods, such as physical and chemical cross-linking, grafting of polymer, and radiation cross-linking.^175,176^ Oral administration of drugs is frequently limited by first-pass metabolism, whereby a significant fraction of the administered dose is metabolized in the gastrointestinal tract and liver before reaching systemic circulation, resulting in reduced bioavailability at the target site. pH-sensitive hydrogels have been developed to address this limitation by protecting drugs from premature degradation and enabling controlled, site-specific release along the gastrointestinal tract. These hydrogels can be formulated in various delivery systems, including microspheres, nanoparticles, and emulsion-based carriers, which have demonstrated superior therapeutic efficacy, improved drug stability, and enhanced patient compliance compared to conventional dosage forms. Various examples of formulations based on different pH-sensitive polymers are summarized in Table 2.

**Table 2:** Formulations Based on Different pH-Sensitive Polymers

Drug Delivery System	Polymer	Model Drug	pH Sensitivity	Release Pattern	ref
Hydrogel	Chitosan-gelatin cross-linked with genipin	Metformin	pH 1.2	Immediate release	^211^
Hydrogel	Hydroxyethyl acryl chitosan and sodium alginate	Paracetamol	pH 7.4	Controlled drug release in the intestine	^212^
Nanofiber Hydrogel	Silk	Doxorubicin	pH 4.5	Sustained release	^213^
Hydrogel	Salecan grafted Poly(2-acrylamido-2-methyl-1-propanesulfonic acid) (PAMPS)	Insulin	pH 7.4	Controlled release	^214^
Polymer Network Hydrogel Bead	Polyacrylamide-*g*-Gum acacia (PAM-*g*-GA)	Gabapentin	pH 6.8	Swelling of hydrogel beads and diffusion	^215^
Electrospun Nanofibers	Polyvinyl alcohol and poly(acrylic acid) loaded with pH-sensitive dye bromothymol blue	Ciprofloxacin	pH 7 and 8.5	Immediate release	^216^
Polymeric Micelles	*N*-naphthyl-*N*, *O*-succinyl chitosan (NSCS) and *N*-octyl-*N*, *O*-succinyl chitosan	Curcumin	pH 6.8 and 7.4	Sustained release	^217^
Polymeric Micelles	Methyloxy-poly(ethylene glycol)-blockpoly[dopamine-2-(dibutylamino) ethylamine-l-glutamate]	For magnetic resonance imaging	pH 5.5	-	^218^

Hydrogels are less stable due to their swelling properties. This feature makes them fragile and restricts their use. Incorporation of clay, use of metal oxide and metal nanoparticles into hydrogel is one of the ways to make hydrogel more stable, which increases its mechanical strength. These are known as “nanocomposite hydrogels”. Nanocomposite hydrogels, also called as hybrid hydrogels, are defined as hydrated polymeric networks, either physically or covalently cross-linked with each other and/or with nanoparticles or nanostructures.^177^ The hydrogel formed with poly­(vinyl alcohol) (PVA) was found to be efficient with enhanced antimicrobial activity of Ciprofloxacin. Simultaneous formation of silver nanoparticles (NPs) and cross-linking of PVA using citric acid is an interesting facet of this research.^178^ A dual role of citric acid was observed; as a cross-linker and stabilizer. It provided pH-dependent swelling and drug release due to the free carboxylic groups on it. It was observed that at pH 7.4, the nanocomposite hydrogel had higher swelling and release of Ciprofloxacin than at pH 2.5. (Figure 15). Along with that, silver NPs were incorporated into the hydrogel. The incorporation of silver NPs caused an increase in antibacterial activity. Moreover, when Ag NPs were incorporated into the drug-loaded hydrogel, the release of ciprofloxacin from the hydrogel was even more prolonged. Ag NPs–loaded PVA hydrogels exhibited clear zones of inhibition against *Staphylococcus aureus* and *Escherichia coli*, confirming their antibacterial efficacy. Notably, the diameter of the inhibition zone for both bacterial strains increased with higher citric acid content, indicating a synergistic effect on antibacterial performance. Furthermore, incorporation of Ag NPs, ciprofloxacin, and citric acid into the PVA matrix resulted in significantly enhanced antibacterial activity against *S. aureus* and *E. coli* compared to pristine PVA hydrogels. Overall, the PVA-based hydrogel system incorporating citric acid and silver NPs represents a versatile platform that can be extended to other therapeutic agents, with the potential to significantly enhance their antibacterial efficacy.

**Figure 15:**
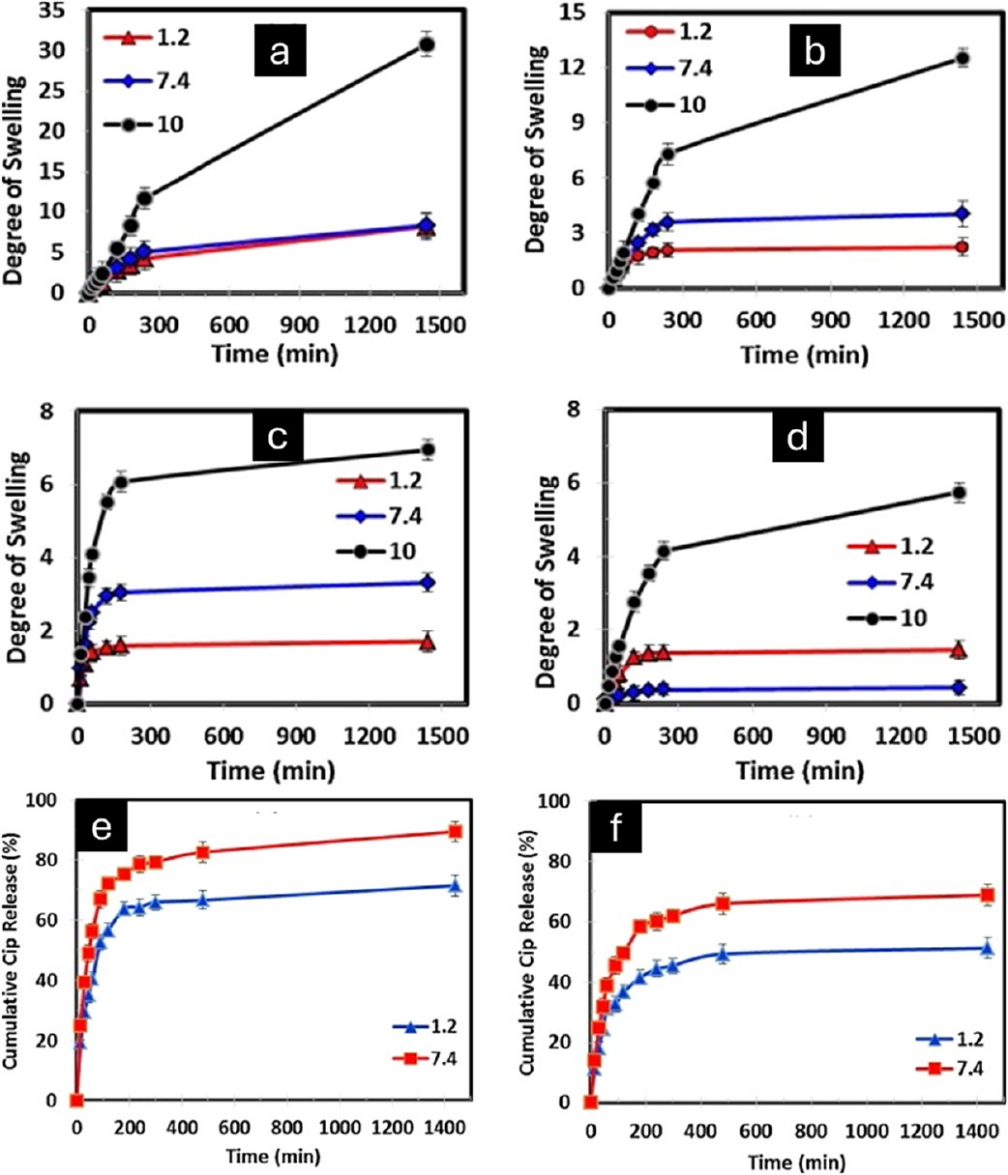
pH-dependent swelling behavior of PVA-C10 (a), PVA-C20 (b), PVA-C30 (c), and PVA-C30-Ag (d) nanocomposite hydrogels as a function of time. Cumulative release of ciprofloxacin from PVA-C30 (e) and PVA-C30-Ag (f) hydrogels as a function of time in buffer solutions at pH 1.2 and 7.4. For both formulations, the differences in drug release between pH 1.2 and pH 7.4 are statistically significant after 45 min (*P* < 0.05). Data are presented as mean ± standard deviation (*n* = 3). Reprinted with permission from ref 179. Copyright 2020, Elsevier.

Another study where an Ag NP-based hydrogel system of carboxymethyl chitosan-PVA was used to study the antibacterial effect of Naproxen.^180^ In a hydrogel prepared from carboxymethyl chitosan-PVA, Ag-NPs were incorporated. pH-dependent swelling behavior of the polymeric ratio was observed; at pH 2.1, the swelling ratio was higher when the ratio of carboxymethyl chitosan-PVA was 3:1, whereas at pH 7.4, the swelling ratio was higher than at pH 2.1 at a 1:3 weight ratio of carboxymethyl chitosan-PVA. Also, at higher concentrations of silver nanoparticles, swelling was found to be increased. The effect of naproxen loading and release was affected by the number of Ag-NPs. A higher amount of Ag-NPs caused less loading of naproxen. Also, the *in vitro* release of hydrogels at pH 2.1 and 7.4 was observed to be reduced with an increased number of Ag-NPs. Antibacterial activity of the hydrogel was higher as both the polymers and Ag-NPs have their antibacterial activity. This is another study where the use of natural polymers leads to the development of a sustainable drug delivery system with increased efficacy. Iron oxide NPs were incorporated into a hydrogel made up of poly­(acrylic acid) grafted onto chitosan for the encapsulation of doxorubicin (DOX).^181^ In this developed system, DOX release was observed to be increased up to 68.8% at pH 5.4 from 25.6% at pH 7.4. The interaction between carbonyl groups of DOX reacts with amine groups of chitosan, forming an imine bond, and this bond gets hydrolyzed more easily at acidic pH than alkaline pH, leading to drug release from the polymeric network. Thus, the pH responsiveness of the polymer increased the drug release in the nanocomposite hydrogel and facilitated controlled drug delivery.

Chitosan is one of the widely used biodegradable polymers that is used to develop novel drug delivery systems. A hydrogel developed from chitosan and polyvinylpyrrolidone using aminopropyletriethoxysilane (APTES) as a cross-linker to achieve pH-controlled release of Cefixime.^182^ Cefixime is associated with low bioavailability; hence, loading into a hydrogel using chitosan was found to be effective. In simulated gastric fluid, slow release of Cefixime was observed. About 81.6% of Cefixime was released in 12 h at pH 1.2. The prepared hydrogel showed pH-dependent swelling behavior. It was observed that lower pH is associated with maximum swelling. Also, a direct correlation between the amount of cross-linker APTES and the stability of the hydrogel was found. Similarly, as the amount of cross-linker in hydrogel increased, antibacterial activity also increased, expressed as the diameter of a zone of inhibition, it increased up to 2 cm where a higher amount of cross-linker APTES was present, from 1.4 cm where APTES amount was lowest.

Insulin is one of the macromolecules that undergo first-pass metabolism, and hence its oral bioavailability is low. Therefore, in diabetic patients, it is injected daily. Somehow, it is a painful treatment. To compensate for this, different formulations of insulin for its oral delivery are made by different groups of researchers. It has been observed that polymers have a major role in it, and research on the oral delivery of insulin is still in process. It is one of the hot topics in the current era.^183–187^ A combination of alginate and gum tragacanth was evaluated for insulin release from the hydrogel.^188^ Hydrogels were prepared by immersing alginate and tragacanth in gelatin solution with chitosan and without chitosan. Insulin release was monitored in simulated gastric (pH 1.2) and intestinal (pH 6.8) fluid at 0, 3, 6, and 24 h. It was observed that at gastric pH, insulin did not release from the gel after an initial small amount. Hence, the release pattern was continued only in simulated intestinal fluid. It was demonstrated that the presence of ionizable groups on both cationic and anionic polymers, i.e., alginate and gum tragacanth, produced pH-responsive behavior in the hydrogel. In an alkaline environment, enhanced electrostatic repulsion between ionized side chains promoted polymer expansion, thereby facilitating the controlled release of insulin from the hydrogel matrix. Incorporation of chitosan into the hydrogel significantly influenced insulin release behavior. While the chitosan-containing and chitosan-free hydrogels exhibited similar release profiles during the initial phase, notable differences emerged at later stages, indicating the role of chitosan in modulating long-term release kinetics. Overall, a combination of pH-responsive polymers has been found to have controlled release of insulin. Alginate made the hydrogel smoother and less elastic, irrespective of chitosan.

### 8.2 Nanofiber

Nanofiber drug delivery system has certain advantages such as more surface area, high porosity, ease of fiber deposition onto other substrates, availability in different structures, ease of fiber functionalization, and mass production is commercially feasible.^189^ Along with these advantages, there are some disadvantages, such as residues of organic solvent can remain in the final product, and an initial burst effect can occur in case of a stimulus-responsive feature.^190^ Different methods, such as template synthesis, drawing, phase separation, self-assembly, and electrospinning, are used to fabricate nanofibers.^190,191^ Combining the pH-responsive polymer and nanofibers helps to achieve the drug release at the specific site. The easiest way to develop pH-responsive nanofibers is by electrospinning a solution containing polymeric electrolytes or their combination. Nanofibers made up of combining poly­(acrylic acid) and poly­(allylamine hydrochloride) showed significant reversible swelling/deswelling due to ionization of PAA in acidic conditions.^192,193^ Similarly, a combination of chitosan and PAA formed a polymeric network that swelled at pH 3. In this, protonation of amine groups of chitosan and hydroxyl groups of PAA was a key factor that induced a pH sensitivity.^194^

Novel electrospun nanofibers of gelatin and another hydrophobic polymer (poly­(lactide-*co*-ε-caprolactone) PLCL) were developed (Figure 16a–d), and the effect of sodium bicarbonate as an inducer of pH-sensitive features into these polymers was studied. Ciprofloxacin was used as a model drug. A combination of gelatin nanofibers and PLCL nanofibers was used.^195^ Drug release was accompanied by pH variation. Gelatin and PLCL fibers without sodium bicarbonate did not show pH-dependent release in phosphate buffer of pH 7.4 and acetic acid pH 5. Both polymers with sodium bicarbonate showed pH-dependent release, but drug release was predominant in gelatin. In the case of PLCL, after burst release, not much ciprofloxacin was released after 45 h. While in gelatin nanofiber, Ciprofloxacin release was 79.8% in acidic pH, which was comparatively more than 58.5% in basic pH after 29 h (Figure 16e). This experiment showed that the use of gelatin in the case of controlled drug delivery can be modified by incorporating pH sensitivity using sodium bicarbonate. Researchers reported that the incorporation of sodium bicarbonate leads to *in situ* CO_2_ generation, resulting in the formation of porous polymeric fibers that facilitate rapid drug release. Among the evaluated systems, gelatin-based fibers exhibited higher antibacterial activity and superior biocompatibility compared to poly­(l-lactide-*co*-caprolactone) (PLCL) fibers. Consequently, pH-responsive gelatin–sodium bicarbonate nanofibers were identified as promising platforms for sustained drug delivery and may be readily extended to the delivery of other therapeutic agents.

**Figure 16:**
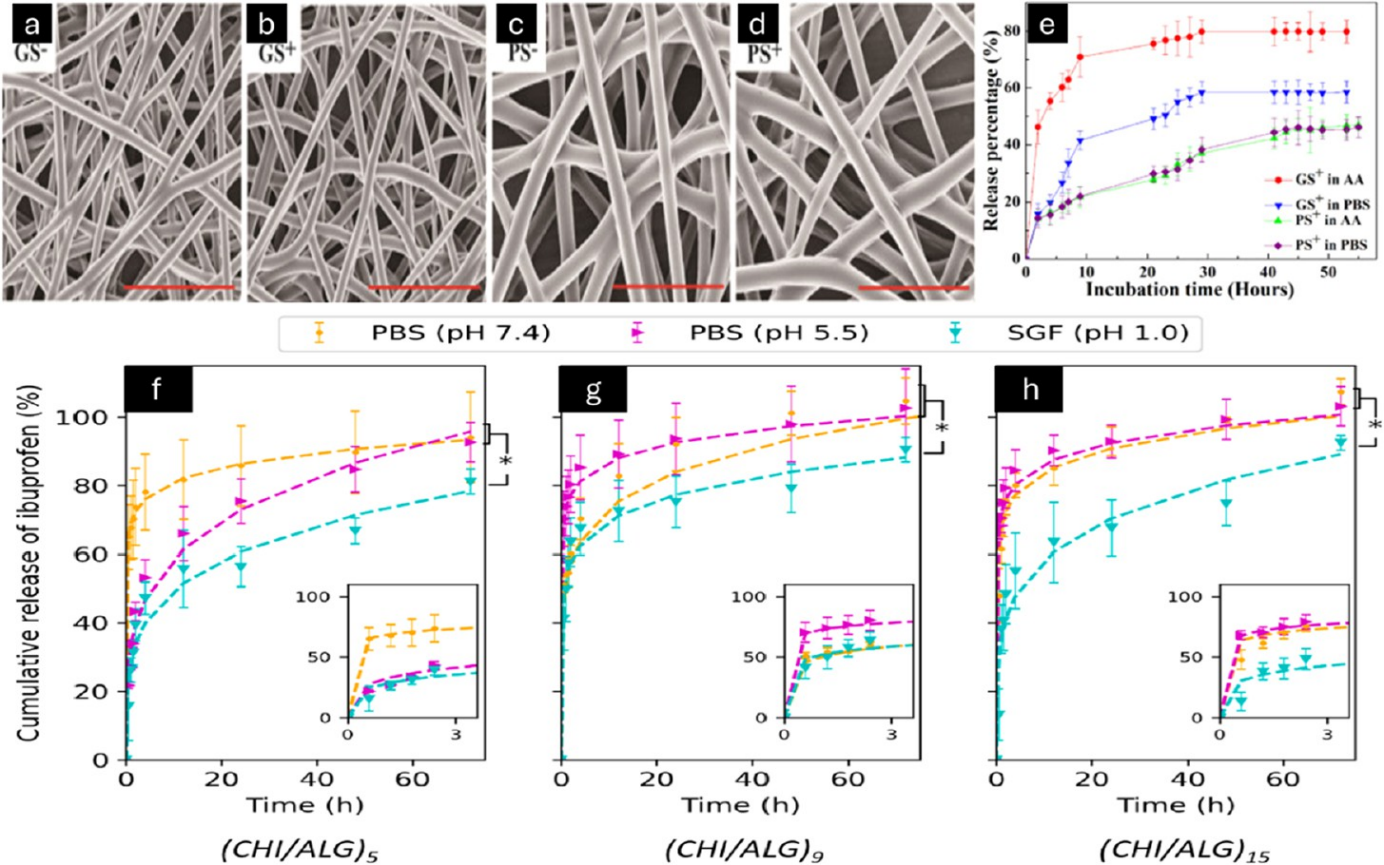
SEM micrographs of the fabricated fibers. SEM images are shown for GS^–^ (a), GS^+^ (b), PS^–^ (c), and PS^+^ (d) fibers after cross-linking. *In vitro* release profiles of ciprofloxacin from GS^+^ and PS^+^ electrospun fibers evaluated in phosphate-buffered saline (PBS, pH 7.4) and acetate buffer (AA, pH 5.0) (e). Reprinted with permission from ref 198. Copyright 2017, Elsevier. Drug-release profiles in phosphate-buffered saline (PBS, pH 7.4), pH-adjusted PBS (pH 5.5), and simulated gastric fluid (SGF, pH 1.0) from (CHI/ALG)\_5_ (f), (CHI/ALG)\_9_ (g), and (CHI/ALG)\_15_ (h) multilayer assemblies. Release kinetics were analyzed using the Ritger–Peppas (power-law) model. Statistical significance is indicated by * for comparisons between PBS (pH 7.4 and pH 5.5) and SGF (pH 1.0). Reprinted with permission from ref 104. Copyright 2021, MDPI.

Any injury to the skin causes inflammation, sometimes sepsis at that site, and dressings are applied to heal the wound formed. In case of chronic injuries, diabetic foot or burn conditions get worse. Hence, this is another area where a site-specific drug delivery system will work like magic. A variety of wound dressings, including patches and films composed of NP-based antimicrobial agents, have been developed to promote infection control and healing. These systems exemplify the effective use of smart polymeric materials in biomedical applications. In one such approach, antibacterial drugs were incorporated into polymeric nanofibers using a simple electrospinning technique. Wound healing is typically accompanied by pH fluctuations; during the stagnation phase of healing, the local pH generally remains in the range of 7–8, a condition that has been strategically exploited in this study. At this pH range, nitrofurazone is released from electrospun nanofibers composed of a polymer matrix containing 15% Eudragit S100, prepared using an optimized solvent mixture of ethanol and N, N-dimethylformamide. The nanofibrous architecture was observed to disintegrate at pH ≥ 7, resulting in rapid drug release within approximately 15 min. Eudragit S100 forms a protective coating around the nanofibers under acidic conditions but readily dissolves under alkaline pH, thereby enabling pH-triggered drug release at the wound site.^196^ Thus, this study contributed to the development of a simple and potential wound dressing made up of a pH-responsive polymer.

Layer-by-layer (LbL) electrospinning offers a versatile and reproducible strategy for surface modification and polymer grafting onto nanofibrous substrates.^197^ In this approach, negatively charged poly­(lactic-*co*-glycolic acid) (PLGA) electrospun fibers were first fabricated, and a radiofrequency plasma gradient was applied to introduce and enhance negative surface charges on the PLGA fibers. Subsequently, a pH-responsive polyelectrolyte complex composed of chitosan and alginate was assembled onto the PLGA nanofibers using LbL deposition to enable controlled ibuprofen release. The polymers were deposited as alternating layers, forming a conformal coating on the nanofiber surface. Drug–polymer interactions within the multilayer system were primarily governed by the polycationic nature of chitosan, which modulated ibuprofen retention and release. As a result, the multilayer-coated nanofibers functioned as an ibuprofen reservoir, exhibiting slower drug release under acidic conditions (pH 1) compared to neutral pH (Figure 16f–h). Overall, this system demonstrated significant potential as a controlled drug-delivery platform capable of bypassing extensive gastrointestinal metabolism.

### 8.3 Polymeric Micelles

Polymeric micelles are nanoscale self-assembled structures formed from amphiphilic block copolymers, consisting of a hydrophobic core and a hydrophilic shell. Their unique architecture enables efficient encapsulation of hydrophobic drugs while providing colloidal stability in aqueous environments. Among various smart nanocarriers, pH-responsive polymeric micelles have attracted considerable attention because they can exploit pH gradients present in physiological and pathological environments, such as the gastrointestinal tract, tumor extracellular matrix, and intracellular endolysosomal compartments.^199,200^ pH responsiveness is typically achieved by incorporating ionizable functional groups (e.g., tertiary amines, imidazole, or carboxylic acids) into one or more polymer blocks. Protonation or deprotonation of these groups within specific pH ranges induces micelle swelling, destabilization, or complete disassembly, resulting in controlled drug release at the target site.^201,202^

The pH-responsive behavior of polymeric micelles primarily originates from the incorporation of ionizable and acid-labile components within their polymer architecture. This includes the protonation of weakly basic polymer blocks, such as poly­(histidine), poly­(β-amino esters), and poly­(2-dimethylaminoethyl methacrylate) (PDMAEMA), which increases their hydrophilicity under acidic conditions and induces micelle swelling or destabilization. Conversely, deprotonation of weakly acidic blocks at higher pH values generates electrostatic repulsion along the polymer chains, leading to micelle expansion. In addition, the presence of pH-cleavable linkers, such as hydrazone, cis-aconityl, and ketal bonds, enables acid-triggered bond cleavage and micelle disassembly. Collectively, these mechanisms ensure micellar stability at physiological pH (∼7.4) while allowing rapid and site-specific drug release under mildly acidic tumor conditions (pH 6.5–6.8) or within endosomal and lysosomal compartments (pH 4.5–5.5).^203,204^

One of the most widely studied systems is PEG-*b*-poly­(histidine) micelles for tumor-targeted delivery of DOX. Nguyen et al. demonstrated that these micelles remain stable at pH 7.4 but undergo rapid disassembly in acidic tumor environments, resulting in enhanced intracellular drug release and improved cytotoxicity against MCF-7 breast cancer cells.^205^ Similarly, PEG-*b*-poly­(histidine) micelles with high encapsulation efficiency (>90%) for DOX, exhibited superior antitumor efficacy and reduced systemic toxicity in xenograft mouse models compared with free drug.^206^ Poly­(β-amino ester) (PBAE)–containing block copolymers are another important class of pH-responsive materials. Luo et al. developed dual pH/redox-responsive mixed micelles composed of mPEG-*b*-PBAE derivatives for controlled DOX release. The micelles showed rapid drug release in acidic and reducing tumor-like conditions and significantly enhanced cytotoxicity toward HepG2 cells.^207^ More recently, Wang et al. reported mPEG–PBAE micelles loaded with temsirolimus for renal cell carcinoma therapy. The micelles exhibited pH-triggered hydrophobic-to-hydrophilic transitions, leading to improved tumor inhibition and reduced adverse effects *in vivo*.^208^ pH-responsive polymeric micelles have also been investigated for oral drug delivery to overcome first-pass metabolism and harsh gastric conditions. Micelles based on ionizable methacrylate or amino-functional polymers can remain intact under acidic gastric conditions and release their payload in intestinal or colonic pH environments.^209,210^

Cabral and co-workers reported pH-responsive polymeric micelles with cross-linked cores for mRNA delivery. These micelles protected mRNA at physiological pH and rapidly released it under endosomal pH conditions (pH 5.5–4.5), significantly enhancing protein expression both *in vitro* and *in vivo*.^204^ pH-responsive micelles incorporating aggregation-induced emission (AIE) probes have been developed for combined imaging and therapy. Dai et al. demonstrated PEG–PEI–PCL-based micelles showing pH-activated fluorescence and controlled drug release, enabling simultaneous cancer cell imaging and chemotherapy.^55^

Despite promising preclinical results, clinical translation of pH-responsive polymeric micelles faces challenges such as batch-to-batch reproducibility, long-term stability, and scale-up. Nevertheless, several polymeric micelle formulations have entered clinical evaluation, demonstrating the translational potential of this platform.^199,202^ pH-responsive polymeric micelles represent a versatile and powerful nanocarrier platform for site-specific drug and gene delivery. Through rational polymer design and careful physicochemical characterization, these systems can achieve precise spatiotemporal control over therapeutic release. Continued efforts toward scalable synthesis, in-depth toxicity evaluation, and clinical validation are expected to further advance their role in next-generation biomedical applications.

### 8.4 Extracellular Vesicles (EVs)

The delivery of peptides across the human eye and skin for disease treatment is challenging due to the presence of the outermost barrier, stratum corneum and cornea, which are mainly composed of tight junction lipids with a thickness of 10–30 μm and 540–560 μm, respectively.^219,220^ Various strategies, including active and passive approaches, have been implemented over the years for enhanced delivery of drug molecules.^221^ Figure 17 shows a general schematic for the delivery of stimuli-responsive polymeric EVs platform for ocular or systemic/dermal applications of drug(s). EVs, with their intrinsic characteristics including low immunogenicity, innate stability, and ability to cross the biological barriers, have made them potential carriers, favoring the delivery of small and large molecular drugs. These nanovesicles have a size ranging from 30 to 150 nm. EVs containing proteins, lipids, and nucleic acids have shown wide biological functions in the treatment of eye diseases, including diabetic retinopathy, age-related macular degeneration, and autoimmune uveitis, among others.^222^ Also, EVs have been shown to deliver various therapeutic molecules, including curcumin, paclitaxel, doxorubicin, siRNAs, miRNAs, and hyaluronidase, among others, for the amelioration of various skin ailments, including bacterial, fungal, and parasitic infections, which can be both local and systemic.^223–225^

**Figure 17:**
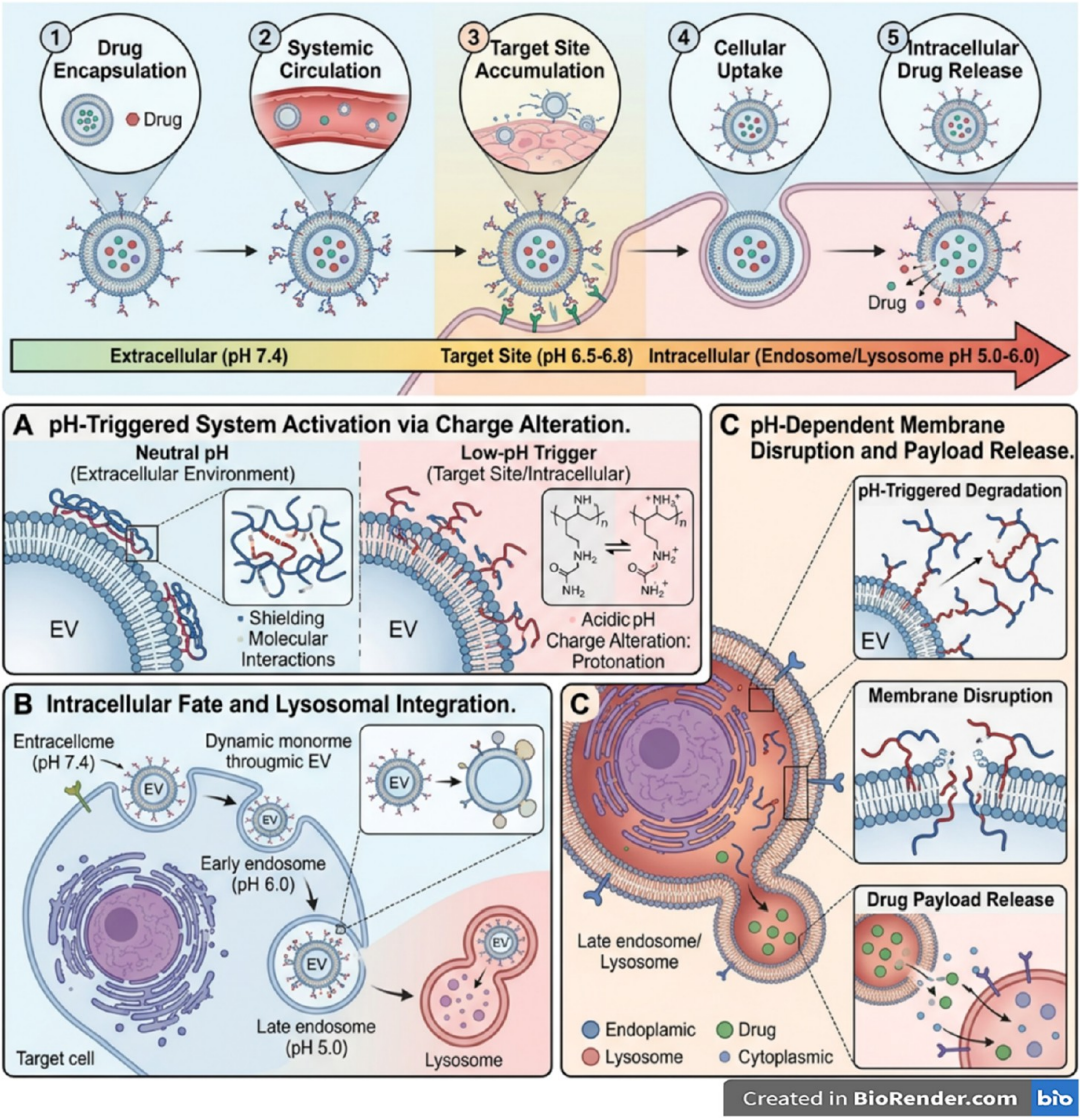
Schematic illustration of pH-responsive polymeric vesicles/micelles enabling site-specific drug delivery and intracellular release. The upper panel depicts the sequential process of (1) drug encapsulation, (2) systemic circulation at physiological pH (7.4), (3) preferential accumulation at acidic target sites (pH 6.5–6.8), (4) cellular uptake via endocytosis, and (5) intracellular drug release within endolysosomal compartments (pH 5.0–6.0). The lower panels detail the underlying mechanisms: pH-triggered system activation through charge alteration, where polymer assemblies remain compact under neutral conditions but undergo protonation-induced charge changes in acidic environments (a); intracellular trafficking of the nanocarrier through early and late endosomes to lysosomes (b); and pH-dependent membrane disruption and polymer degradation, leading to vesicle destabilization and efficient payload release into the cytoplasm (c). This figure highlights how spatially defined pH gradients can be exploited to achieve controlled, stimuli-responsive drug delivery in biomedical applications. This image has been created as a Creative Common using the BioRender software (https://www.biorender.com/).

EVs are small membrane vesicles that are released by specific cells in tissues or cells in response to internal or external stimuli. EVs possess the capability to be targeted toward tissues, preserving their inherent biological functionalities. Phospholipid bilayers can serve as a vehicle for incorporating hydrophobic drugs via hydrophobic interactions, whereas hydrophilic drugs can be enclosed within the central region of artificial EVs. Luan et al. reported the fabrication of a chitosan scaffold that was thermally stable and exhibited pH-responsive behavior, adsorbing EVs in mildly acidic conditions and releasing them at neutral/alkaline pH.^226^ EVs isolated using the scaffold (T-CS-E) were comparable to those obtained by ultracentrifugation in terms of size, morphology, and marker expression. *In vitro* findings suggested T-CS-E promoted endothelial cell proliferation, migration, and angiogenesis. Anti-inflammatory effects were observed by reducing pro-inflammatory cytokines. *In vivo* studies in a mouse wound model showed accelerated wound closure compared to controls, enhanced granulation tissue formation, collagen deposition, and vascularization, and reduced inflammatory cell infiltration. Taken together, the findings concluded that the thermally stable, pH-responsive chitosan scaffold is an effective platform for EVs isolation and controlled release. This system significantly improves wound healing by combining structural support with bioactive exosome delivery, offering a promising strategy for regenerative medicine and chronic wound treatment.

Another study by Zhang et al., a multifunctional hydrogel incorporating multiple dynamic cross-links that respond to both glucose and pH changes, enabling controlled release of adipose-derived stem cell EVs in diabetic wound environments.^227^ Physicochemical characterization confirmed the hydrogel’s injectability, self-healing, and stability under physiological conditions. The hydrogel exhibited strong dual responsiveness, with swelling ratios increasing significantly at acidic pH (∼5.5) and in the presence of high glucose (∼25 mM), while maintaining mechanical integrity after repeated deformation cycles. EV release was markedly accelerated in diabetic wound-like conditions, reaching about 75–80% within 48 h, compared to less than 40% under normal conditions. *In vitro* assays demonstrated that EV release was significantly accelerated under high-glucose and acidic conditions, promoting fibroblast proliferation, angiogenesis, and M2 macrophage polarization while pro-inflammatory cytokines (IL-1β, TNF-α) decreased by nearly 50%. Mechanistic analysis revealed downregulation of the Notch/NF-κB/NLRP3 signaling pathway, reducing inflammation and enhancing tissue regeneration. *In vivo*, diabetic mouse wounds treated with the hydrogel closed by roughly 90% at day 14, compared to ∼60% in controls, with significantly higher collagen deposition and neovascular density (*p* < 0.001). These findings confirmed that the hydrogel’s glucose/pH-triggered EV release effectively modulates inflammation and promotes tissue regeneration, offering a promising therapeutic approach for chronic diabetic wounds. In a review by Liu et al., discussions are well encouraged on the constructed pH-responsive artificial EVs via a convenient way for targeting delivery toward tumors.^228^

In another study, pH-sensitive i-motif-modified EVs were fabricated to deliver DOX efficiently to the cancer cells.^229^ The study engineered i-motif DNA-coated EVs as a pH-sensitive carrier for anticancer drugs and confirmed successful surface modification without altering EVs’ size (∼100–150 nm) or morphology. Drug loading efficiency was approximately 65–70%, and release profiles showed minimal drug leakage at physiological pH (7.4) but a rapid release of over 80% within 24 h under acidic conditions (pH 6.0), simulating the tumor microenvironment. *In vitro* cytotoxicity assays using cancer cell lines revealed that i-motif-coated EVs-loaded with DOX reduced cell viability to ∼30%, compared to ∼55% for unmodified EVs and ∼60% for free drug, indicating superior targeted delivery. The discussion highlighted that the i-motif structure undergoes conformational changes in acidic environments, enabling controlled drug release while maintaining biocompatibility. The authors concluded that i-motif-functionalized EVs offer a promising smart delivery platform for cancer therapy, combining the advantages of natural exosomes with pH-triggered release for enhanced therapeutic efficacy. Certain thermoresponsive polymers, such as poly­(*N*-isopropylacrylamide) (PNIPAm) and poly­(*N*-vinyl caprolactam) (PNVCL), exhibit lower critical solution temperature (LCST) values that are below physiological temperature.^230,231^ To this end, PNVCL thermoresponsive polymer was used to encapsulate EVs and its role as a therapeutic agent for blood clot lysis was studied. The results revealed the fabricated thermoresponsive EV gel formulation with immense potential in thromboembolic diseases.^232^ The antitumor treatment was studied using EVs, attached with pH-responsive 3-(diethylamino) propylene (DEAP) block and CD44 receptor-interacting with specific ligand, hyaluronic acid. In general, these investigations emphasize the novelty of utilizing EVs equipped with a specific ligand targeting tumor receptors, as well as a pH-responsive substance DEAP, to deliver anticancer drugs effectively.^233^ A pH-sensitive pullulan-based EV was prepared to target DOX nanoparticles into the liver cancer cells.^234^ A pH-responsive fibroin-based EVs hydrogel was prepared, encapsulating miRNA (miR-675), which was implanted in Ischemic hind limbs that showed increased retention and stability of EVs.^235,236^

EVs possess multiple intrinsic advantages, such as biocompatibility, nanoscale size, and natural ability to cross biological barriers, that make them highly promising candidates for ocular drug delivery. However, current research in this area remains limited, with only a few studies reported to date. The advantages include (a) isolation of these entities from the human body enhances safety precautions; (b) inheriting the characteristics of the originating body and manifesting a diverse range of qualities, including but not limited to anti-inflammatory and chemotactic attributes.^237,238^ Keratoconus and corneal injury are the two most common corneal diseases that have been highly focused on. A new method was proposed for combining EVs with thermosensitive chitosan hydrogels for healing corneal injury.^239^ This was administered at room temperature and transformed into a gel at body temperature so that the drug adhered to the lesion site for a longer period. The study demonstrated that the healing process was accelerated, leading to reduced corneal scarring through enhanced regeneration of both the corneal epithelium and stroma.

Taken together, pH-adaptive polymers integrated with EV delivery systems represent a transformative approach for targeted and sustained therapeutic release in both skin and ocular applications. By leveraging the natural responsiveness of these polymers to local pH variations, such as acidic wound environments or ocular surface conditions, these platforms enable controlled EV release, improved retention, and enhanced bioactivity. Experimental evidence across multiple studies demonstrates accelerated tissue regeneration, reduced inflammation, and improved structural repair, highlighting their potential to overcome limitations of conventional delivery methods. This synergy between smart polymer design and EV-based therapy offers a promising pathway for next-generation regenerative treatments, particularly for chronic wounds and ocular surface disorders.

### 8.5 Microneedles (MNs)

The transdermal route is one of the routes to administer the drug into the body, where the localized effect is given, or drugs are injected into the bloodstream. However, injecting with a hypodermic needle is somehow painful and tedious. To combat this, the MN array is one alternative method that is gaining popularity in recent times. MNs are cone-shaped structures that exist in the size range of 250–2000 mm in height.^240^ MNs have several pros, such as reduced pain, inflammation, and injury, rapid penetration in the bloodstream, comparatively feasible in controlled delivery of small molecules, macromolecules, DNA, RNA into the skin, easy to store with reduced cost and waste.^241^ Whereas, these are associated with a few cons such as their administration requires proper technique, slow drug release in case of solid MNs, in metallic MNs, traces of metal can remain beneath the skin and cause irritation, swelling, etc.^241^ Use of a pH-sensitive polymer is one of the ways to make a stimulus-responsive MN. These polymers make the MN sensitive to pH. Basically, these polymers contain hydrophilic and ionic functional groups, which allow for structural changes in the polymer network by degradation, swelling and rupture. These changes bring about the drug release at the site of action, controlled drug release. In the acidic pH of the epidermis, the pH-responsive MNs release the drug at a controlled rate. For example, hollow MN made up of biodegradable poly­(d, l-lactic-*co*-glycolic acid) (PLGA) containing a sodium bicarbonate system is widely used as a drug carrier system. In this, the acidic pH of skin, i.e., 4.5–5.5, causes carbon dioxide generation, and this carbon dioxide subsequently makes a porous PLGA layer and thus releases the drug.^242^ The conventional method of preparation of MNs is based on photolithography, which is difficult, expensive, and requires infrastructure.^243^ Hence, researchers developed an easy and comparatively cheap method of microfabrication of MNs using pH-sensitive polymers. Figure 18 represents an MN for drug delivery for various biomedical applications.

**Figure 18:**
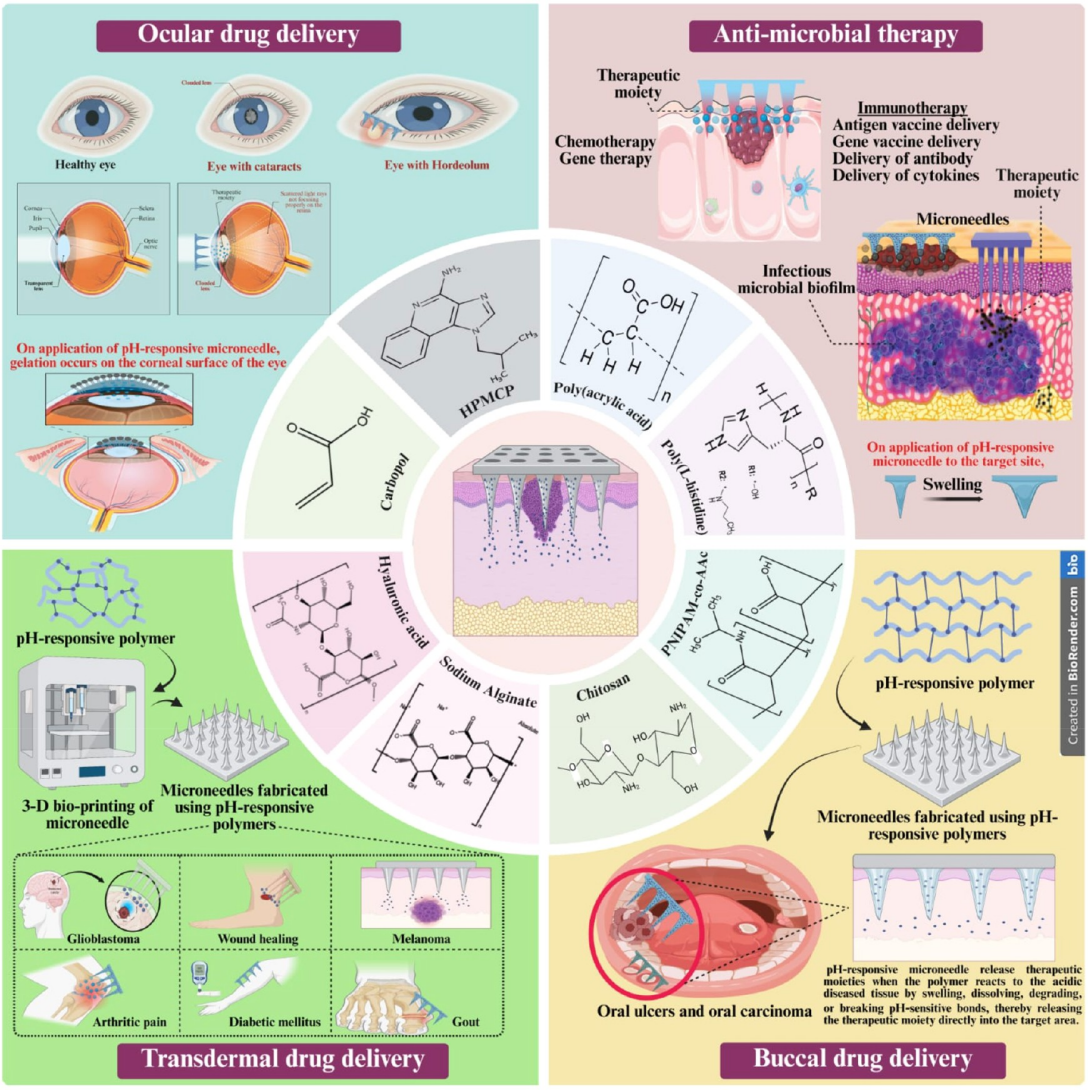
pH-responsive microneedle for drug delivery for various biomedical applications. This image has been created as a Creative Common using the BioRender software (https://www.biorender.com/).

An MN patch of diclofenac sodium using different proportions of chitosan and PVA was formed. It was seen that a composite with a 1:6 ratio of chitosan and PVA had the highest tensile modulus of 8.667 MPa. Increased PVA concentration leads to higher tensile strength. From the developed MN patch, about 20.17% release of the diclofenac in phosphate buffer of pH 7.4 was achieved after 30 h. It showed swelling-dependent and controlled release of diclofenac.^244^ As discussed previously, insulin is one of the most challenging to administer orally. To overcome this limitation, a group of scientists developed transcutaneous MNs made up of glucose and pH-responsive polymers. An efficient delivery system composed of polymeric vesicles made up of water-soluble pillar arene (WP5), having pH sensitivity and paraquat-ended poly­(phenylboronic acid) (PPBA-G) with glucose sensitivity (Figure 19).^245^ These dual-responsive polymeric vesicles-loaded with insulin and glucose oxidase exhibited insulin release more than at 100 mg/dL at 400 mg/dL of glucose. A decrease in the pH from 7.5 to 5 caused more insulin release. The transdermal MN array patch showed controlled insulin release over 12 h as compared to insulin injection. This painless and promising system could have potential applications in diabetes treatment. This is another example where polymers showed their versatility and are applied for the development of a transdermal patch of insulin.^246^ Similarly, an MN patch of pH-sensitive NPs encapsulated with insulin and pH-insensitive NPs with glucose oxidase and catalase was formulated for transcutaneous insulin delivery. These NPs were fabricated using pH-sensitive amphiphilic block copolymer, methoxy poly­(ethylene glycol) (MPEG)-*b*-poly­(2-(hexamethyleneimino) ethyl methacrylate), i.e., mPEG-*b*, which was synthesized by reversible addition–fragmentation chain transfer (RAFT) polymerization. Prepared MNs were found to release insulin under mildly acidic conditions in a hyperglycemia-induced mouse model. Thus, this opens up the application of glucose-responsive MN patches in the delivery systems associated with glucose sensitivity.^247^

**Figure 19:**
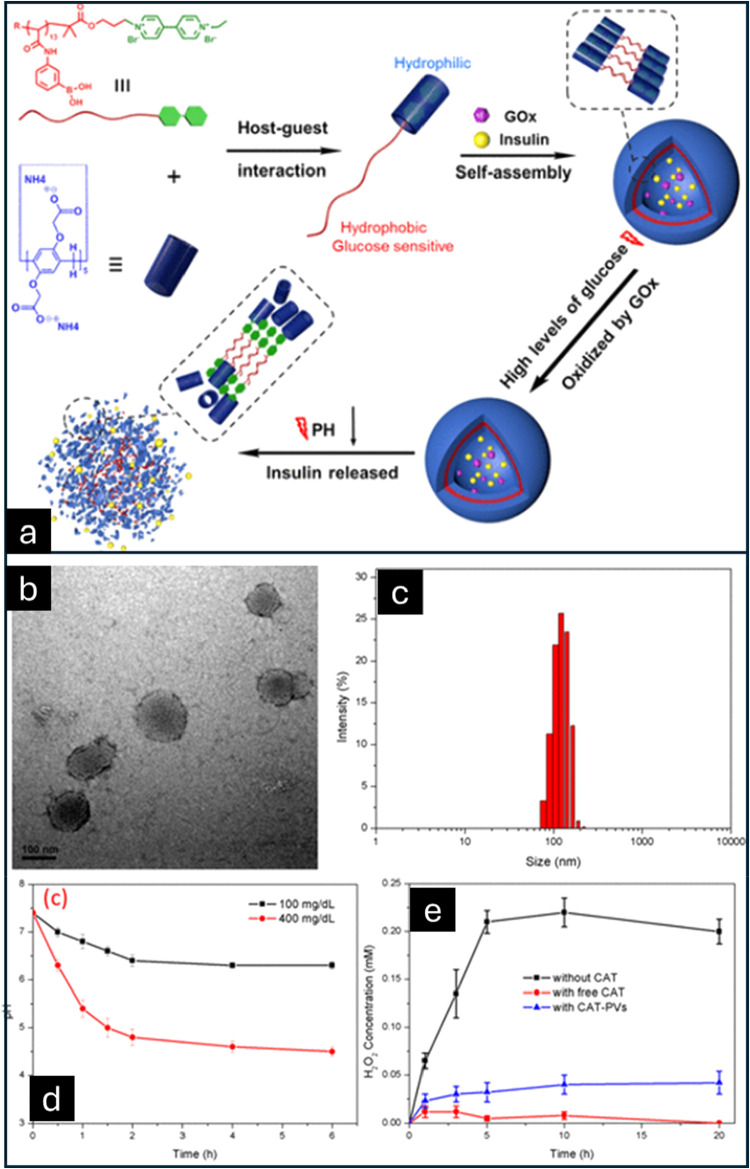
Schematic representation of a glucose-responsive polymeric vesicle (PV) system for regulated insulin delivery. Amphiphilic building blocks formed via host–guest interactions between hydrophilic hosts and hydrophobic, glucose-sensitive guests self-assemble into vesicles encapsulating insulin and glucose oxidase (GOx). The vesicles remain stable under normoglycemic conditions, while elevated glucose levels are oxidized by GOx, generating acidic byproducts that lower the local pH, destabilize the vesicle structure, and trigger controlled insulin release (a). Characterization of drug-loaded polymeric vesicles (PVs), (Ins + GOx)-PVs. Representative TEM image of (Ins + GOx)-PVs (b); corresponding particle size distribution (c); pH variation of PBS at two glucose concentrations in the presence of (Ins + GOx)-PVs (GOx: 0.05 mg mL^–1^) (d); and H_2_O_2_ generation in PBS (pH 7.4) containing 400 mg dL^–1^ glucose, catalyzed by GOx in the absence or presence of catalase (GOx: 0.05 mg mL^–1^; CAT: 0.0125 mg mL^–1^) (e). Reprinted with permission from ref 245. Copyright 2020, American Chemical Society.

### 8.6 Clinically Viable Gene Delivery Systems

Clinically, gene delivery systems are the most important application for the successful delivery of siRNA, DNA, RNA, plasmid, and small genes.^248^ Several target-specific delivery approaches are discussed below. In the case of the delivery of biomolecules, a polymer is complexed with nucleic acid. During transport to the target site and intracellular trafficking, the complex gets destabilized due to polymer response, successively allowing nucleic acid. Figure 20 shows the mechanism of gene delivery in various biomedical applications.

**Figure 20:**
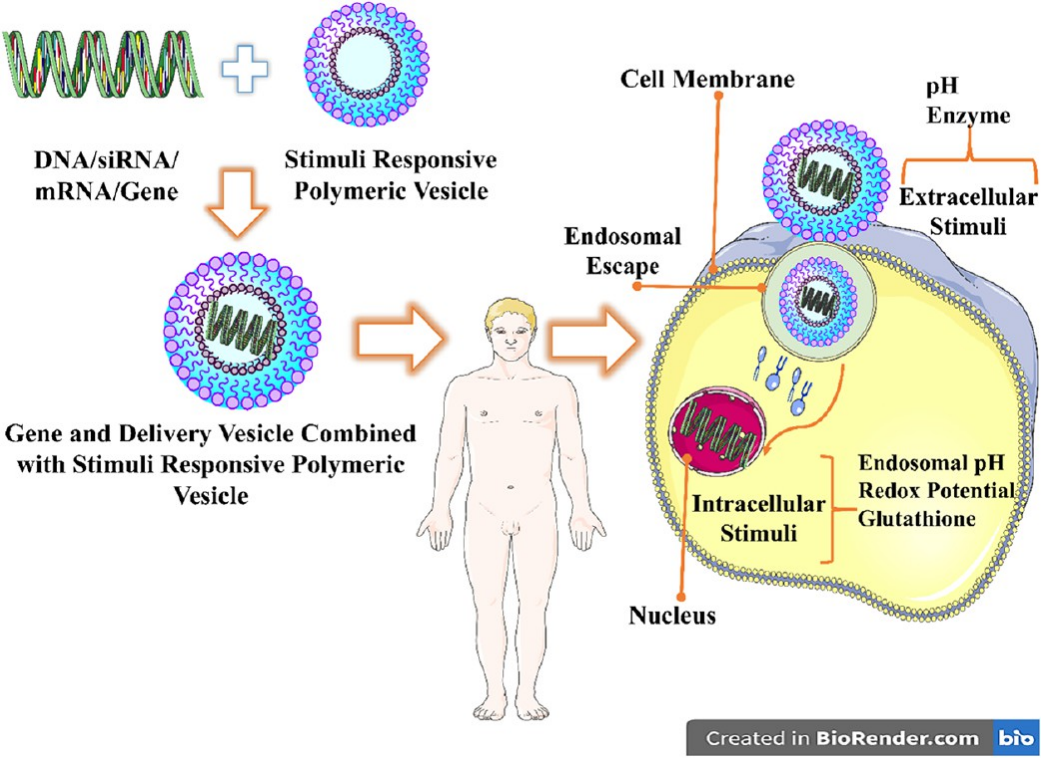
Gene delivery for Biomedical applications. This image has been created as a Creative Common using the BioRender software (https://www.biorender.com/).

Administration of proteins in the body is achieved by coating them in polymeric layers. These nanocarrier systems of polymers facilitate protein and peptides at different sites, i.e., the gastrointestinal tract, colon, etc. Microbeads encapsulated with bovine serum albumin (BSA) using alginate and aminated chitosan were developed.^249^ The utilization of oppositely charged polymers and the formation of a smart pH-responsive polyelectrolyte complex were characteristic features of this study. Alginate microbeads with a higher amount of aminated chitosan coating were observed to have maximum swelling at a lower pH of 1.2, whereas at pH 6.8 and 7.4, swellings were found to be reduced. *In vitro* release of BSA in the gastrointestinal tract was observed to be different in simulated gastric, intestinal, and colon fluid. In pH 1.2, BSA release was lower than that of the release at higher pH values. Antibacterial activity and biodegradability were comparatively higher with an increased concentration of aminated chitosan (Figure 21a). Thus, the combined use of alginate and aminated chitosan can be employed for the target-specific release of proteins/peptides in the gastrointestinal tract. The use of both natural and comparatively low-cost polymers can create an easy, economic and versatile drug delivery system.

**Figure 21:**
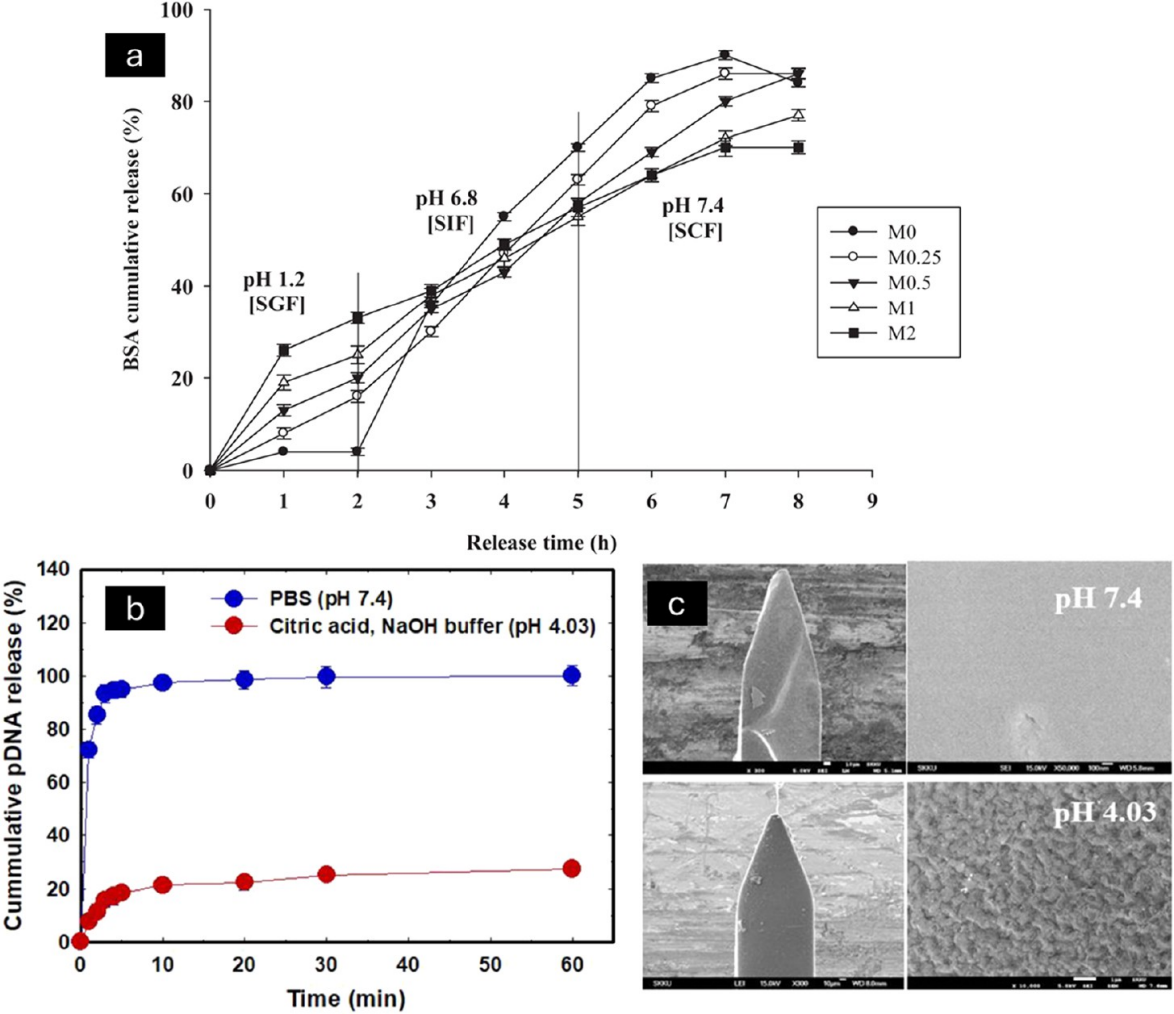
Influence of gastrointestinal tract conditions on the *in vitro* release behavior of bovine serum albumin (BSA) (a). Reprinted with permission from ref 249. Copyright 2016, Elsevier. Release profile of polyplexes. *In vitro* release behavior of polyplexes from microneedle (MN) arrays under various pH conditions (b). Representative SEM images of MN arrays after immersion in different pH environments (c). Reprinted with permission from ref 253. Copyright 2018, Elsevier.

Safe and painless administration of a DNA vaccine is a tedious process. Different ways are employed for the efficient DNA vaccine delivery, such as intramuscular or intradermal electroporation, biojector, gen gun, and microinjection, among others.^250^ These are associated with shortcomings such as painful administration, discomfort, and require skilled professionals. To combat this, various carrier systems are used; among them, polyelectrolyte multilayer assemblies are widely used nowadays.^251^ A combination of MN and polyelectrolyte multilayer produces an efficient system for penetration into the skin.^252^ A triblock pH-sensitive charge reversal copolymer made up of oligo (sulfamethazine)-*b*-poly­(ethylene glycol)-*b*-poly­(β-aminoester urethane) (OSM-*b*-PEG-*b*-PAEU) developed by researchers as a release layer in a polyelectrolyte multilayer assembly on MNs for the DNA vaccines.^253^ The copolymer works on the principle of the charge reversal mechanism. Due to the presence of cationic tertiary amine and anionic sulphonamide groups at pH 4.03, the polymer exhibited a positive charge, and at pH 7.4 it switches to a negative charge. Consequently, the PMA structure on MNs rapidly disassembled, releasing the polyplexes. In contrast, polyplex release was significantly retarded under acidic conditions (pH 4.03) (Figure 21b). SEM analysis of MNs incubated in PBS (pH 7.4) and citric acid/NaOH buffer (pH 4.03) confirmed complete PMA disassembly under physiological conditions (Figure 21c). After being injected into the body, it becomes negatively charged, which disassembles the different layers of polyplex coating containing amphiphilic polyethylenimine-deoxycholic acid polymer (DA3) and DNA vaccines encoded with Alzheimer’s antigen on an MN. Through disassembling, DNA gets released into the cells. Here, MNs successfully delivered DNA into human embryonic kidney 293T cells and RAW 264.7 macrophage cells. After injecting the developed Aβ DNA vaccine in mice, and quantification by ELISA, Aβ expression was found to be higher. Thus, MNs coated with DNA vaccines efficiently reacted to antigen-producing components in the skin layer, i.e., dermis and epidermis and produced immunity against Alzheimer’s disease. Thus, the effective use of pH-responsive polymer opened another way to DNA vaccine delivery.

Cell-engineered based pH-responsive DNA triple nano switches for cellular communication with a sensitive responsive pH range were developed by the group of researchers. Here, researchers observed that these DNA nano switches responded to physiological pH changes from pH 6.5 to 7.5 and converted to a double-chain structure from a three-chain structure. This conversion enabled the connection of another cell set with complementary single-stranded DNA.^254^ This approach is effective for developing cell-based therapeutics against oncogenesis.

Delivery of small interfering RNA (siRNA) for silencing of genes at the target site has significant importance in cancer and other diseases.^255^ These siRNAs are effectively delivered through nanoparticles as carrier systems. They facilitate the permeability and retention of therapeutic agents at the target/tumor site. Dual responsive cationic unimolecular nanoparticles were developed by the multiarm star H40-poly­(aspartic acid-(2-aminoethyl disulfide)-(4-imidazolecarboxylic acid))-poly­(ethylene glycol i.e., (H40–P­(Asp-AED-ICA)-PEG). The main objective was to facilitate siRNA delivery to a tumor site. It led to endosomal/lysosomal escape and decomplexation of siRNA inside the target cell. This characteristic feature was introduced by conjugating the imidazole group into the polymer. The polymer showed dual sensitive characteristics, pH and redox, which facilitated the controlled release of siRNA in breast cancer cell lines (MDA-MB-468) where EGFR was overexpressed. In a buffer solution of pH 5.4, the release of siRNA from nanoparticles was observed to be higher than at neutral pH buffer, and the addition of 10 mM GSH increased the release to 81.4%. This observation showed that the polymer exhibited dual sensitization. Also, these nanoparticles showed excellent GFP down-regulating and induced less cytotoxicity than a commercial agent.^256^

Co-delivery of chemotherapeutic agents and genetic material has gained considerable attention as a strategy to enhance therapeutic efficacy and overcome drug resistance. In this context, smart drug-delivery systems based on polymeric nanoparticles have been developed to coencapsulate paclitaxel and small interfering RNA (siRNA), enabling synergistic anticancer effects through combined chemotherapy and gene silencing. The polymeric system was made up of polyethylenimine-*block*-polylactic acid (PEI–PLA) and poly­(ethylene glycol)-*block*-poly­(l-aspartic acid sodium salt) (PEG- PAsp).^257^ Polyethylenimine (PEI) facilitated efficient endosomal escape, enabling the delivery of siRNA into the cytoplasm, where it silenced the vascular endothelial growth factor (VEGF) gene. Meanwhile, the PEG–poly­(aspartic acid) (PEG–PAsp) coating rendered the dual-loaded NPs nearly charge-neutral, improving their colloidal stability and biocompatibility. These neutral NPs made a stable drug delivery system and protected siRNA from degradation by nucleases and further enabled them to inhibit the target proteins and enhance the action of the coloaded drug. Here, the mutually stimulating effect of chemotherapeutic agents and siRNA induced inhibitory action on 4T1 breast cancer cells proliferation and orthotopic breast cancer animal models. A pH-sensitive DNA nanohydrogel to facilitate mRNA delivery into cells was developed.^258^ X-shaped DNA scaffolds and DNA linkers with pH-responsive i-motif sequences released mRNA at lysosomal pH of 4.5–5 upon dehybridization. Thus, a nano hydrogel in which DNA scaffolds were encapsulated. Also, it was observed to have high stability and biocompatibility compared to commercial liposomes. Thus, the nanogel system was found to be competitive for *in vivo* mRNA delivery.

## 9 The Rise of pH-Responsive Revolution in Biomedical Applications

Over the past two decades, pH-responsive materials have emerged as a transformative class of “smart” systems in biomedical science, driven by their ability to sense and respond to subtle pH variations in physiological and pathological environments. Distinct pH gradients, such as those found along the gastrointestinal tract, within endosomes and lysosomes, and in diseased tissues including tumors, inflamed sites, and infected wounds, have provided a powerful biological trigger for designing responsive platforms with high spatial and temporal precision. By integrating ionizable functional groups or pH-labile linkages into polymers, nanoparticles, hydrogels, and vesicular assemblies, researchers have enabled controlled changes in solubility, swelling, self-assembly, or degradation in response to pH fluctuations. This pH-responsive revolution has reshaped approaches to drug delivery, diagnostics, tissue engineering, and bioimaging by enabling site-specific release, enhanced therapeutic efficacy, and reduced systemic toxicity. From orally administered systems that protect drugs in acidic gastric environments to nanocarriers that selectively release payloads in the acidic tumor microenvironment or intracellular compartments, pH-responsive platforms exemplify precision medicine in action. As advances in polymer chemistry, nanotechnology, and bioengineering continue to converge, pH-responsive systems are increasingly positioned at the forefront of next-generation biomedical technologies, offering adaptable, minimally invasive, and highly efficient solutions to longstanding clinical challenges.

### 9.1 pH-Responsive Platforms for Cancer Immunotherapy

Even though the complex microenvironment of the tumor is challenging in cancer therapy, it offers a scope for targeted drug delivery. Various pH-responsive polymers are efficient carriers in targeted therapies and cancer diagnostics. In this, drug release at the tumor is mainly catalyzed by the acidic tumor environment (pH < 5).^259^ Ionizable functional groups are incorporated in polymers, and this is used to form a nanocarrier drug delivery system. These ionizable moieties become unstable in the acidic microenvironment of the tumor, disturbing the hydrophilic–lipophilic layer, leading to drug release at the tumor site and causing therapeutic effect. Various anticancer agents are associated with different drawbacks, like oxaliplatin, which is rapidly deactivated from the bloodstream, has low bioavailability, lacks tumor selectivity, and these were surmounted by designing a pH-sensitive drug delivery system.^260^ An illustration of pH-responsive polymer-based tumor cell targeting and microenvironment is shown in Figure 22.^261^

**Figure 22:**
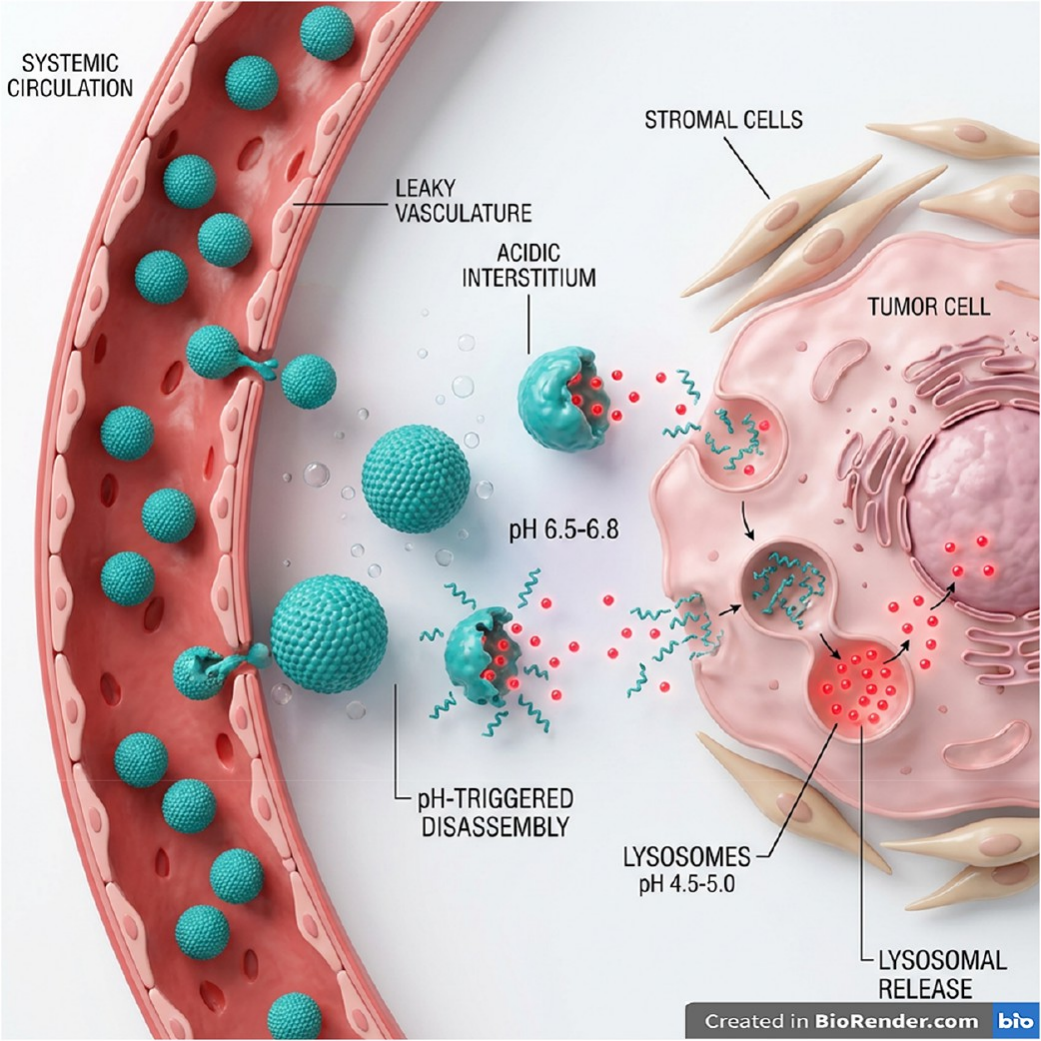
Schematic illustration of pH-responsive nanoparticle-mediated drug delivery to tumor tissue. Systemically administered nanoparticles extravasate through the leaky tumor vasculature via the EPR effect and encounter the mildly acidic tumor interstitium (pH 6.5–6.8), inducing partial destabilization and drug release. Following cellular uptake, trafficking to lysosomes (pH 4.5–5.0) triggers complete nanocarrier disassembly and rapid intracellular drug release. Stromal components are depicted to represent the heterogeneous tumor microenvironment. This image has been created as a Creative Common using the BioRender software (https://www.biorender.com/).

Different ways have been employed to develop pH-controlled drug release, such as the use of acid-sensitive chemical bonds to conjugate drugs to a nanocarrier. To enable efficient intracellular drug delivery, a wide range of pH-responsive polymers have been exploited to harness the pH gradients encountered along the endocytic pathway. These systems incorporate ionizable functional groups, such as imidazole and poly­(β-amino ester) moieties, as well as pH-labile chemical linkages, including acetal, hydrazone, vinyl ester, and ortho-ester bonds. In addition, pH-sensitive cell-penetrating peptides and cationic polymers exhibiting pH-dependent protonation have been employed to promote endosomal destabilization and escape. Collectively, these design strategies facilitate controlled intracellular release by responding to the progressive acidification from early endosomes to lysosomes.^262^ Polymers containing acid-labile chemical bonds remain stable at physiological pH. These bonds are present within the polymeric structure, either in the backbone with block copolymers junctions or in the side chains. The acidic environment of endosomes, lysosomes, or tumor tissue results in destruction and destabilization of acid-labile bonds, thus accelerating the drug release. In another way, pH-sensitive drug release is based upon acid-labile bonds, which are used for acid-labile linkers to conjugate drugs covalently to carrier molecules or to the surface of nanostructures, producing prodrugs which are inactive until the linker is hydrolyzed.^263,264^

In cancer diagnosis or surgery, it is important to identify tumors from adjacent normal cells or tissues. The pH-responsive polymers were used to identify and build up tumor diagnostic systems. The pH-responsive polymeric micelles were used as contrast agents for magnetic resonance imaging for targeting and capturing cancerous tissue images.^265^ It is based on the Fe_3_O_4_-loaded diblock copolymer polyethylene glycol- poly amino ester (PEG–PAE), which maintains a micellar state without precipitation. At pH ≤ 6.8, polymeric micelles readily dissociate due to ionization of the tertiary amine moieties in the poly­(β-amino ester) segments, leading to micellar destabilization and subsequent release and aggregation of encapsulated Fe_3_O_4_ NPs. The preferential accumulation of these NPs in acidic microenvironments enhances local signal intensity, thereby improving the accuracy and sensitivity of signal measurement. In this system, PEG–PAE polymeric micelles were coloaded with the fluorescent dye tetramethyl rhodamine isothiocyanate (TRITC). The resulting pH-responsive polymeric fluorescent nanoprobes exhibited nonlinear amplification of tumor-associated signals, enabling enhanced imaging contrast in acidic tumor regions.^266^ They help identify tumor tissue of histological type, driver mutation, as well as the detection of acute treatment responses faster than conventional imaging approaches.^267^ Multidrug resistance and poor tumor response are the obstacles associated with chemotherapy. pH-responsive polymer targets acidic extracellular microenvironment and solid tumor intracellular organelles. Polymeric nanocarriers possibly avoid the use of surfactants and promote the accumulation of the drug at the tumor sites by enhanced permeability and retention effects (EPR).^268^

DOX is an effective treatment in many cancer types, but has some restrictions because of its poor pharmacokinetics. To overcome such limitations associated with chemotherapy and Dox’s toxicity, various alternative approaches have been adopted by researchers. Among them, targeted drug delivery has shown impressive results.^269^ Polyelectrolyte composition of chitosan and o-carboxymethyl chitosan as a pH-responsive carrier was developed for the oral delivery of DOX.^270^ Researchers reported enhanced absorption of DOX throughout the small intestine, particularly in the jejunum and ileum. The tumor-targeting capability and therapeutic efficacy of DOX were significantly improved using pH-responsive polymeric micelle systems as well as drug–polymer conjugates incorporating acid-labile linkages, which enable selective drug release in acidic tumor microenvironments. Figure 23 shows the *ex vivo* confocal images of DOX, DOX + CsA, DOX: CS-NPs and DOX: CS/FITC-CMCS-NPs absorption in excised rat ileum.

**Figure 23:**
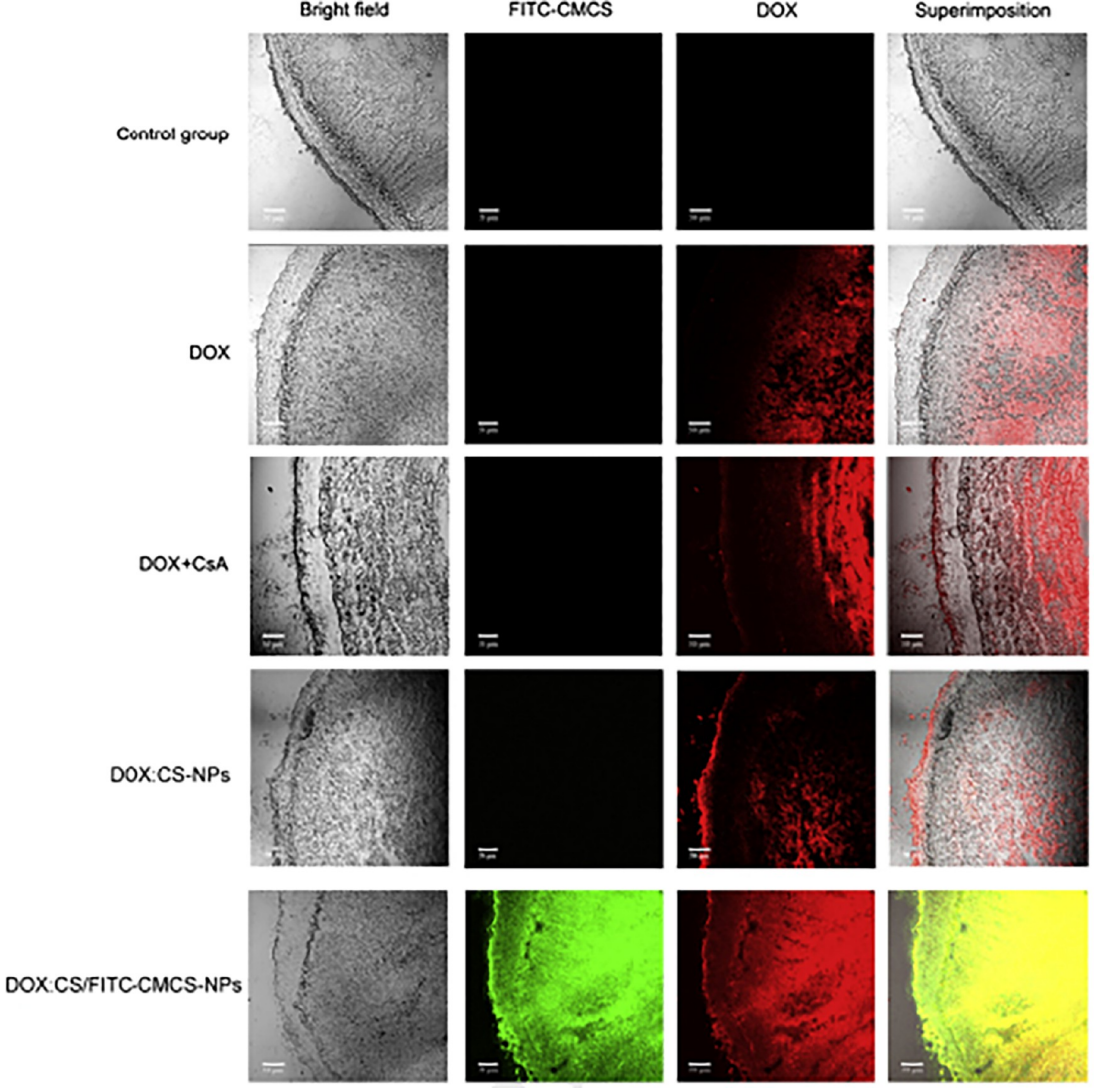
*Ex vivo* confocal images of DOX, DOX+ CsA, DOX: CS-NPs and DOX: CS/FITC-CMCS-NPs absorption in excised rat ileum. Reprinted with permission from ref 270. Copyright 2013, Elsevier.

Conjugation of pH-responsive polymers with magnetic NPs is an innovative approach in cancer treatment.^271^ Chitosan-coated magnetic NPs targeted DOX to the tumor site under a magnetic field.^271^ With enhanced targeting, higher anticancer activity was observed. Researchers synthesized supermagnetic (Mn, Zn) Fe_2_O_4_ NPs encapsulated with PLGA-coated chitosan. The DOX-releasing efficiency of the developed NPs was pH dependent. At higher concentration, i.e., 250 g/mL, DOX PLGA showed more efficient anticancer activity than neat DOX. Other anticancer agents like camptothecin, paclitaxel, gambogenic acid, and tamoxifen are also referred to, and examples are compiled with polymer systems and polymeric carriers in Table 3.

**Table 3:** pH-Responsive Polymer Formulations Developed as Anticancer Drug Carriers

Anticancer agent	Polymeric system	Polymeric carrier structure	ref
Camptothecin (C0)	Poly(β-amino ester) methyl ether poly(ethylene glycol) (MEPG-PAE)	Polymeric micelles	^266^
Paclitaxel (PTX)	NK105 (Polyaspartic acid and PEG)	Polymeric micelles	^272^
	Polyethylene glycol-poly(*N*-(acryloyloxy) succinimideco-butyl methacrylate mPEG-*g*-p(NAS-*co*-BMA)		^273^
Doxorubicin (DOX)	Poly(l-histidine)	Polymeric micelles	^274^
	Chitosan (Ch)	Chitosan-coated magnetic nanoparticles	^271^
	Chitosan/carboxymethyl chitosan (CS-CMCS)	Nanoparticles	^270^
Gambogenic acid (GNA)	Poly(acrylic acid)-*b*-polycaprolactone (PAAc-*b*-PCL)	Polymeric micelles	^275^
Tamoxifen	Chitosan	Nanoparticles	^276^
	Poly[2-(diisopropylamino) ethyl methacrylate]-Poly [2-(methacryloyloxy)ethyl phosphorylcholine] (FAMPC-DPA)	Polymeric micelles	^277^

### 9.2 pH-Sensitive Nanoprobe-Based Fluorescent Imaging and Image-Guided Surgery

Surgical intervention remains one of the primary treatment modalities for cancer management. During tumor resection, accurate identification of tumor boundaries and metastatic spread is critical to ensure complete removal. Incomplete excision significantly increases the risk of tumor recurrence. Moreover, inadvertent damage to surrounding healthy tissues during surgery may result in organ dysfunction or long-term complications, underscoring the need for precise intraoperative tumor delineation. Hence, intraoperative technologies that help surgeons visualize tumor margins during operations can improve the long-term survival of cancer patients. The cheap and practical technology is always necessary to differentiate tumor from normal tissues attached to the tumor, which helps to reduce severe damage to patients.^275,278,279^ The pH-responsive NPs dependent approach for imaging a broad range of tumors uses nonlinear amplification of tumor acidosis signals.^267^ The neutralized ionizable amines and amphiphilic block copolymers remained in micellar and self-quenched forms, thereby fostering resonance energy transfer. This made access to the acidic tumor microenvironment, or was internalized into endocytic organelles in tumor endothelial cells. The system demonstrated broad tumor specificity with an extraordinary tumor-to-blood ratio in different tumor models. By following imaging, it continued to study in image-guided surgery with various clinically compatible fluorescent cameras.^274^ Real-time tumor acidosis–guided detection and surgical resection enabled precise tumor delineation, leading to significantly improved long-term survival in tumor-bearing mice.^280^ The basic principle of Fluorescence-guided surgery is shown in Figure 24.^281^

**Figure 24:**
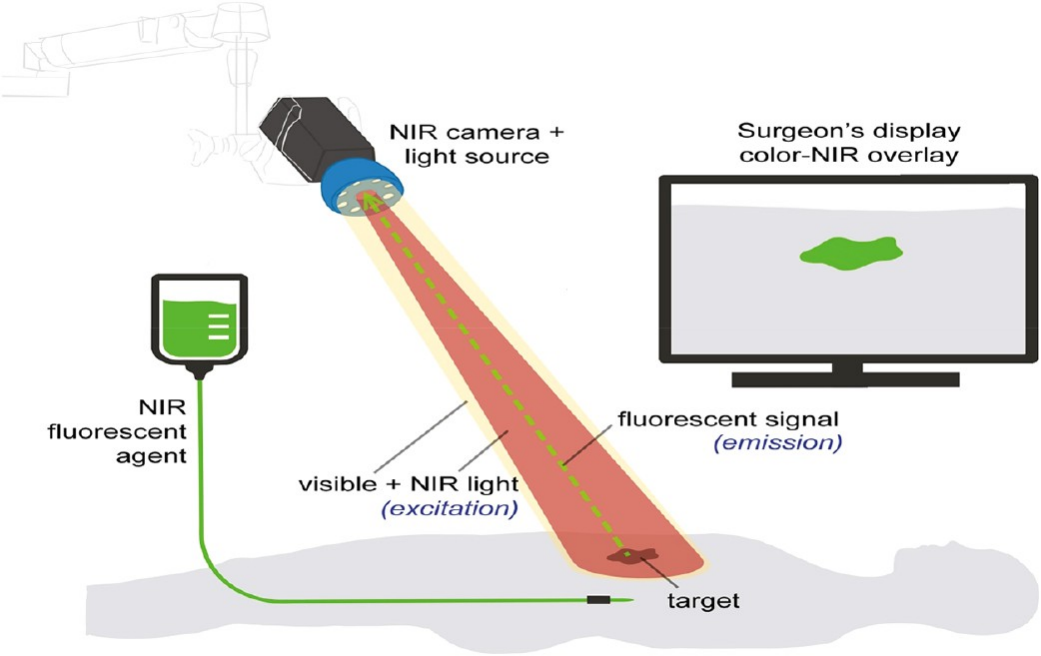
Basic principle of fluorescence-guided surgery. Reprinted with permission from ref 281. Copyright 2022, Elsevier.

### 9.3 pH-Responsive Nanoprobe-Based Magnetic Resonance Imaging

In addition to their role in drug delivery, pH-responsive polymers have been extensively investigated for applications in disease diagnosis. In cancer diagnostics, magnetic resonance imaging (MRI) is widely employed, typically relying on contrast agents to enhance lesion visibility. However, conventional MRI contrast agents often lack tumor specificity, leading to nonspecific distribution, reduced imaging efficiency, and the requirement for relatively high systemic doses. To address these limitations, pH-responsive amphiphilic block copolymers have been developed as targeted delivery platforms for MRI contrast agents. By exploiting the acidic tumor microenvironment, these smart polymeric systems enable selective accumulation and activation of contrast agents at tumor sites, thereby significantly enhancing MRI signal intensity and diagnostic accuracy within tumor regions. A cancer-recognizable MRI contrast agent has been developed by using pH-responsive polymeric micelles. The polymeric micelles were developed where p (l-His) blocks were the pH-responsive component. The imidazole groups of p (l-His) blocks were protonated, which caused a broken micellar structure in an acidic tumoral environment.^282^ Paramagnetic gadolinium (Gd 3+) chelates were MRI contrast agents that enhanced signal intensity after exposure to water molecules as micelles broke. The protonated imidazole groups of p­(l-His) block after extravasation, forming positively charged agents and facilitating the accumulation of agents through interaction of positively charged agents and the cellular membrane. *In vivo* studies demonstrated that cancer-recognizable, pH-responsive contrast agents produced significant MRI signal enhancement in the CT26 murine tumor model in Balb/c mice. In contrast, tumors treated with nontargeted (insensitive) contrast agents exhibited negligible changes in MRI signal intensity over time, highlighting the superior tumor specificity and imaging efficacy of the responsive contrast system. Cancer recognizable contrast agents enabled the detection of a small tumor (3 mm^3^) *in vivo* within a few minutes. Fe_3_O_4_ NPs have been widely encapsulated within pH-responsive polymeric micelles and employed as MRI contrast agents. *In vivo* evaluation in murine models demonstrated a progressive increase in MRI signal intensity over a 24 h period for the pH-responsive contrast system. In contrast, pH-insensitive Fe_3_O_4_-based contrast agents showed no significant change in signal intensity over time, highlighting the enhanced tumor-specific imaging capability of the pH-responsive micellar platform.^265^ Schematic illustration for the formation, release and imaging process is shown in Figure 25.^283^

**Figure 25:**
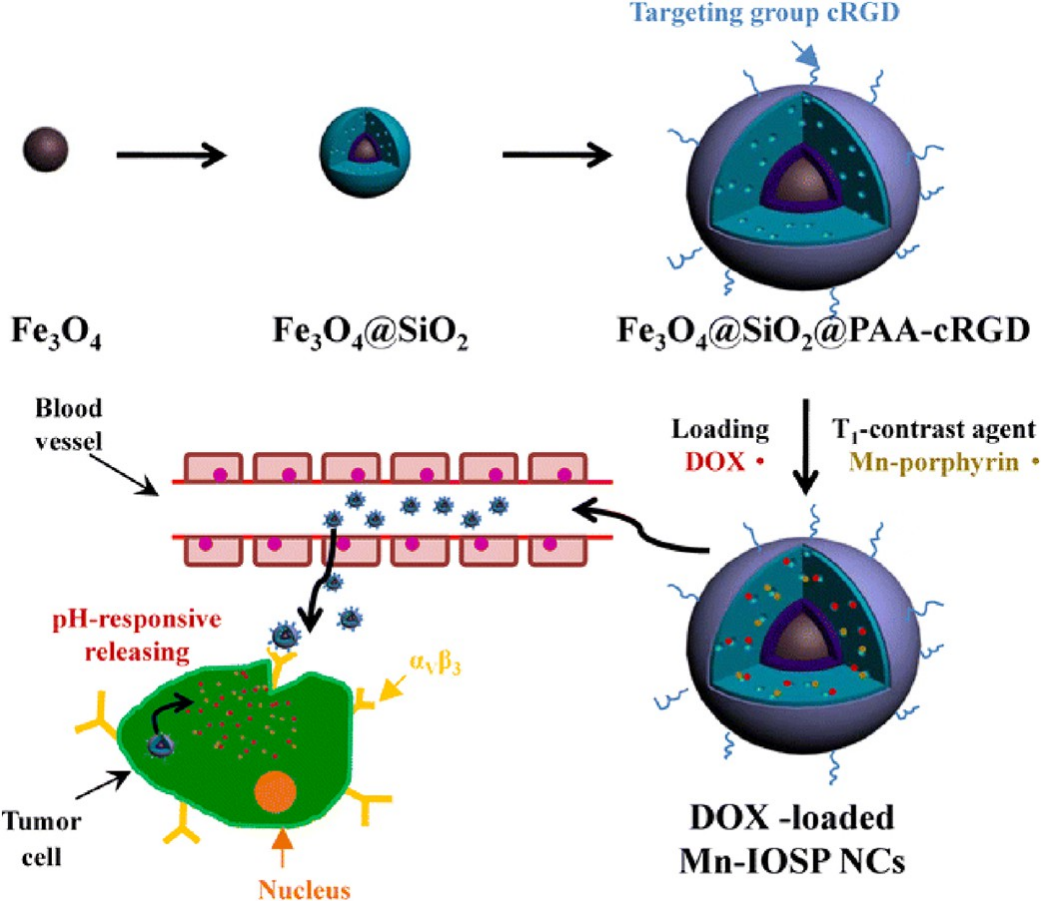
Schematic illustration for the formation, release and imaging process of DOX-loaded Magnetic porphyrin &Fe_3_O_4_@SiO_2_@PAA-cRGD (Mn-IOSP). Reprinted with permission from ref 283. Copyright 2018, Springer Nature.

Although the detection of intracellular dysfunction is difficult, these smart materials have made it possible. One of such examples is the development of a fluorescent nanoprobe. Researchers have developed such a nanoprobe for sensing intracellular pH and imaging. Lysosome is a degeneration cell organelle, containing multiple hydrolases, rich in an acidic environment (pH 4.5–5.5), which is significant for protein degradation. Abnormalities in lysosomes result in many diseases. Hence, monitoring lysosomal pH in such diseases is the main pathological investigation. For this, recently researchers have developed methoxy-based covalent organic framework (COFs), a porous and 2D or 3D material, called as TAPB-DMTP-COF.^284^ It was synthesized from 1,3,5-Tris­(4-aminophenyl) benzene (TAPB) and Dimethoxyterephthaladehyde (DMTP). The imine groups made the COFs pH-responsive and generated pH-dependent fluorescence at the cellular level. Under acidic pH, protonation of imine groups exhibited green fluorescence, and the developed probe demonstrated pH fluctuations from 4 to 7.4 using confocal fluorescence imaging.

### 9.4 pH-Responsive Platforms for Biosensors

The popular application of pH-responsive polymers is the development of insulin delivery systems for diabetes treatment. An appropriate amount of insulin needs to be delivered at the exact time as needed. After the meal, when the glucose level is high, oxidation of glucose to glucuronic acid, catalyzed by glucose oxidase, can lower the pH to around 5.8. This enzyme is widely used in glucose sensing; it is possible to use different kinds of pH-responsive polymers for modulated insulin delivery.^285^ To this end, the glucose-responsive polymer gives a self-regulating insulin system in response to glucose concentration in the blood and controls the concentration of insulin within a normal range. It is based upon a pH-responsive polymer with entrapped GluOx, insulin and catalase.^286^ A polyacid such as gluconic acid was useful as a pH-responsive polymer-based glucose release system.^287^ In this system, oxidized gluconic acid lowered pH, and protonated acidic groups of polyacids led to the shrinkage of hydrogels and the release of entrapped insulin through porous molecular valves for microfluidics. A glucose-sensitive hydrogel containing derivatives of *p*-aminobenzene sulphonamide, synthesized by copolymerization with polymerizable sulfadimethoxine monomer, *N,N*-dimethylacrylamide (DMAAM) and sucrose particles, was used as a porogen.^287^

Roan et al. examined mass-changing pH-responsive hydrogels, which increased sensitivity by increasing the fraction of acrylic acid in the poly­(acrylic acid-*co*-isooctyl acrylate) copolymer.^288^ A hydrogel film was made up of UV photosensitive resin. It resulted in a change in color of the hydrogel, which could be detected. Saccharide moieties of glycopolymers produced a strong interaction with lectins, plant proteins that have a high affinity for specific sugar residues.^289^ A glucose-responsive micellar structure made up of poly­(ethylene oxide)-*block*-poly­(2-glucosyl-oxyethyl acrylate) disrupted micelle and released entrapped insulin in case of high blood glucose.^290^

The application of a pH-sensitive hydrogel for a sensor can be largely extended by adding an intermediate step where an analyte is used to change the pH. For example, a hydrogel-based CO_2_ sensor where CO_2_ gas forms carbonic acid in water, resulting in a pH change and indirectly in the volume of pH-sensitive hydrogels.^291^ pH-sensitive methacrylic copolymers and the production thereof of this invention involves novel multifunctional methacrylic copolymers that show cationic pH-sensitive property and good water solubility under low pH. This copolymer is prepared by anionic polymerization of a tertiary amine methacrylate with a poly­(ethylene glycol) having a methacrylate group. As they are pH-sensitive, they can control the release of the materials that are associated with them based on the pH of the surrounding environment. The copolymers have both hydrophilic and hydrophobic segments, are noncytotoxic, and so can be used in living tissues. This novel copolymer is used in many fields as it exhibits special physical properties. They are associated with various materials like proteins and other pharmaceuticals for the separation of protein and drug delivery; they can also be associated with genetic material to provide a genetic transformation vector and used as gene vectors.^292^ The invention of pH-responsive biodegradable polylactic-acid polymeric micelles and their applications relates to pH-responsive biodegradable polylactic acid (PLA) derivatives capable of self-assembling into polymeric micelles in aqueous environments at pH values of 4 or higher. The PLA derivatives employed possess molecular weights in the range of 500–2000 Da, enabling efficient micellization and biodegradability. Suitable polymers include d,l-polylactic acid, copolymers of d,l-lactic acid with mandelic acid, glycolic acid, caprolactone, or 1,4-dioxane-2-one. These polymers are synthesized via catalyst-free polycondensation of 2-hydroxycarboxylic acid derivatives at elevated temperatures under reduced pressure. The resulting micellar systems are designed for biomedical applications, particularly in pH-triggered drug delivery, where controlled self-assembly and disassembly enable targeted release under specific physiological conditions. The polymeric micelles have sizes of 10 to 21 nm and can entrap a poorly water-soluble drug inside them, and so the poorly water-soluble drugs get solubilized.^293^

pH-responsive mucoadhesive polymer–encapsulated microorganisms represent a targeted delivery strategy in which living microbes are entrapped within a pH-sensitive, mucoadhesive polymer matrix. This platform is designed for enteric delivery in animal hosts, enabling protection of microorganisms in the upper gastrointestinal tract and their site-specific release and retention at desired intestinal regions. The polymeric encapsulated microorganism has three alternating bilayers that are formed of a layer of an enteric pH-responsive methacrylate polymer and a layer of mucoadhesive polymer. This polymethacrylate polymer is on the surface of the microorganisms, and the mucoadhesive polymer is on the outer surface of the encapsulated microorganisms, which binds to the mucosal surfaces of the gastrointestinal tract of a host. The polymethacrylate polymer is stable at an acidic pH, and it degrades at physiological pH so that the microorganisms are released from the polymer bilayers once it reaches the small intestine. The encapsulation provides a prolonged survival, extended retention, and a pH-responsive release of the microorganisms or other components that are encapsulated. This formulation is used for diagnostic, therapeutic, and prophylactic purposes and can alter a host’s microbial composition associated with a condition or other disease state.^294^

## 10 Patent Landscape of pH-Responsive Polymers for Biomedical Applications

The patent landscape for pH-responsive polymers in biomedical applications has expanded significantly over the past two decades, reflecting their growing importance in advanced drug delivery, diagnostics, and tissue engineering. These polymers exhibit reversible physicochemical changes, such as swelling, solubility alteration, or bond cleavage, in response to pH variations commonly found between healthy and pathological tissues (e.g., tumors, inflamed sites, or the gastrointestinal tract). As a result, they enable site-specific and controlled release of therapeutic agents, improving drug efficacy while minimizing systemic side effects.

Patent activity in this domain is dominated by innovations related to polymeric compositions, synthesis methods, functional modifications, and specific biomedical uses, including oral, injectable, and implantable delivery systems (Table 4). Early patents primarily focused on basic pH-sensitive polymer backbones such as poly­(acrylic acid), poly­(methacrylic acid), and chitosan derivatives. More recent filings emphasize multifunctional systems, including pH-responsive hydrogels, polymeric nanoparticles, and dual- or multistimuli-responsive platforms combined with redox or enzyme sensitivity. Overall, the evolving patent scenario underscores both the technological maturity and the competitive intensity of pH-responsive polymers as enabling materials for next-generation biomedical applications.

**Table 4:** Patent Scenario of pH-Responsive Polymer for Biomedical Applications (https://patents.google.com)

Title of Patent	Invention	API/Polymer	Application	Patent Number	Inventors
Compositions having HASE rheology modifiers	pH-responsive composition and method to modify rheological properties of aqueous emulsions	Hydrophobically modified alkali-soluble emulsion (HASE) polymer	Modification of rheological properties of aqueous emulsions	EP1885679B1	H.S.Yang, D.Pakenham, H.Adam, P.Hennaux, B.Chung, S.Frantz
Use of pH-responsive polymer	Use of a pH-responsive polymer to isolate target compounds from a liquid by adsorbing the target compounds via hydrophobic interaction	Any pH-responsive polymer	Selective adsorption by hydrophobic interaction chromatography using pH pH-responsive polymer	US20060189795A1	J.V.Alstine, C.Larsson, R.Palmgren, A.Rudstedt
pH-responsive biodegradable polylactic acid derivatives forming polymeric micelles and uses thereof for poorly water-soluble drug delivery	Polylactic acid derivatives capable of forming micelles in an aqueous solution of pH 4 or above, and having one terminal carboxyl group, and a polymeric micelle composition comprising the polylactic acid derivatives	Polylactic acid derivatives polymer have molecular weight of 500 to 2000 Da	For the solubilization of poorly water-soluble drugs	EP1448710B1	M.Seo, B.Kim, I.Choi, M.Shim
pH-responsive polymer carrier compositions for cytosolic protein delivery	pH-responsive polymer-based protein delivery carriers and compositions.	Random, block or graft copolymer comprising a C2–C8 alkyl group and a carboxylic acid group ionized at pH 7.4 and protonated at pH 5.5–6.0	Delivery of a protein or peptide antigen can induce a cytotoxic T-lymphocyte response	US20100150952A1	P. S.Stayton, S.Foster, A.S.Hoffman
pH-responsive mucoadhesive polymeric encapsulated microorganisms	Encapsulation of microorganism using pH-responsive mucoadhesive polymer to achieve prolonged survival, extended retention and targeted release within the gastrointestinal tract	Polysaccharides, Eg, alginate, cellulose derivatives.	Site-specific delivery to the gastrointestinal tract to treat conditions like Crohn’s disease, ulcerative colitis	US10548844B2	A.C.Anselmo, R.S.Langer, A.Jaklenec
pH-responsive film for intravaginal delivery of a beneficial agent	Use of pH-responsive films, preferably those with an interpenetrating network, for the intravaginal administration of prophylactic and therapeutic agents	A biocompatible, hydrophilic polymer that is positively charged at a first pH and in electronically neutral form at a higher pH, and an alkylene oxide polymer or copolymer	Treatment of vaginal infections, vaginal cleansing, and vaginal lubrication	US20060018951A1	M.Maniar, S.Parandoosh
Pharmaceutical compositions comprising pH-sensitive block copolymers and a hydrophobic drug	Amphiphilic block copolymers, which form supramolecular assemblies or micelles in the nanometric size under favorable conditions, are useful for oral or parenteral delivery of hydrophobic or cationic pharmaceutical agents	diblock copolymer of poly(ethylene oxide) and poly(butyl (alkyl)acrylate-*co*-(alkyl)acrylic acid)	Entrapment and delivery of a hydrophobic drug	EP1638539B1	V.Sant, J.Leroux
pH-sensitive methacrylic copolymers and the production thereof	Novel multifunctional copolymers composed of tertiary amine methacrylates and poly(ethylene glycol) containing methacrylates that exhibit cationic pH-sensitive property and good water solubility under acidic conditions.	pH-sensitive under acidic conditions and water-soluble copolymer	Gene vectors, pharmaceuticals, and protein separation reagents	US6998456B1	S.K.Mallapragada, B.C.Anderson, P.D.Bloom, V.V.Sheares Ashby
Library of pH-responsive polymers and nanoprobes thereof	Polymers contain hydrophobic and hydrophilic segments that are sensitive to pH. The polymers form a micelle, which is sensitive to pH, resulting in a change in fluorescence based on the pH	pH-responsive polymer	Imaging of the cellular or extracellular environment or delivering a drug	US11013818B2	J.Gao, G.Huang, T.Zhao, X.MA, Y.Wang, Yang Li, Baran D.Sumer
pH-responsive self-healing hydrogels formed by boronate-catechol complexation	pH-responsive self-healing hydrogels comprising covalently cross-linked copolymers formed by boronic acid-catechol complexation and methods of synthesizing and using the hydrogels	Boronic acid- catechol cross-linked polymers	Biomedical applications like surgical implants, surgical adhesives and drug delivery systems	US9572910B2	P.B.Messersmith, Lihong He, Dominic E., Fullenkamp

## 11 Challenges, Future Perspectives, and Conclusion

pH-responsive polymeric materials have emerged as versatile platforms for addressing critical challenges in controlled drug delivery, immunotherapy, biosensing, and biomimetic nanomedicine. Table 5 highlights and contextualizes the translational potential of representative pH-responsive polymers, namely chitosan, poly­(methacrylic acid) (PMAA), poly­(β-amino esters) (PBAEs), poly­(2-diisopropylaminoethyl methacrylate) (PDPA), and conducting polymers such as polyaniline, across diverse material formats including injectable hydrogels, electrospun nanofibers, nanoassemblies, extracellular vesicle–mimetic nanocarriers, microneedle systems, and nanofibrous biosensor films. Leveraging the pH gradients characteristic of physiological, pathological, and intracellular environments, these polymer systems demonstrate temporally and spatially controlled release of therapeutic cargoes, enhanced cellular uptake, endosomal escape, and stimulus-regulated signal transduction. PBAE-based injectable hydrogels and PDPA nanoassemblies show strong promise for cancer therapy and immunotherapy through localized cytokine and antigen delivery with validated immune activation in tumor-bearing murine models, while PMAA and chitosan nanofibers enable tumor-acidic pH-triggered drug release and physiologically relevant biosensing, respectively. Conducting polymer nanofibrous films further approach deployment-ready biosensor platforms owing to their reversible protonation-dependent electrical responses. Mapping these systems to Technology Readiness Levels (TRLs 4–6) underscores both their experimental maturity and the remaining translational gaps related to scalability, regulatory compliance, and long-term *in vivo* performance. Collectively, the discussed materials exemplify how rational polymer design coupled with appropriate structural engineering can bridge fundamental stimuli-responsive behavior with clinically relevant biomedical applications, positioning pH-responsive polymers as key enablers in next-generation therapeutic and diagnostic technologies.

**Table 5:** Technology Readiness Levels of pH-Responsive Polymer Systems across Advanced Biomedical Applications: A Case-Study Perspective^a^

pH-responsive polymer category	Materials	Formulation Type	Application area	Highest demonstrated TRL	TRL Justification (Evidence)	ref
Weak polybases	CS	Injectable hydrogel	Gene delivery	TRL 4	*In vitro* and *ex vivo* transfection with mechanistic validation of pH-responsive protonation	^297^
		Electrospun Nanofibers	Biosensors	TRL 5	Functional prototype sensor tested under physiological pH conditions	^298^
Weak polyacids	PAA/PMAA	Swelling hydrogels	Hydrogel drug delivery	TRL 5–6	Reproducible swelling-controlled release demonstrated in animal GI models	^299^
	PMAA	Nanofibers	Cancer immunotherapy	TRL 4–5	Tumor-pH triggered drug release in *in vitro* and murine tumor models	^300^
Synthetic polybases	PBAE	Polyplex NPs	Gene delivery	TRL 4	Reliable cell-line and primary-cell transfection with pH-buffering validated	^301^
	PBAE	Injectable hydrogel	Cancer therapy	TRL 6	Local cytokine delivery validated in tumor-bearing mice with immune activation	^302^
Sharp pH-transition polymers	PDPA	Nanocarriers	EVs	TRL 4	Endosomal pH-triggered disassembly demonstrated *in vitro* and *in vivo* biodistribution	^303^
	PDPA	Nanoassemblies	Cancer immunotherapy	TRL 5	Antigen release and DC activation validated in syngeneic mouse tumor models	^304^
pH-responsive copolymers	PLGA–PEG–PA	DMNs	Vaccines/Immunotherapy	TRL 6	Skin-delivered system validated *in vivo* with antigen-specific immune response	^305^
Natural polymer hybrids	Chitosan–alginate	Hydrogel-EV system	EVs	TRL 4–5	pH-responsive EV release proven in inflammatory animal models	^306^
Conducting polymers	Polyaniline	Nanofibrous film	Biosensors	TRL 6	Near-deployable biosensor prototypes validated under physiological conditions	^307^

a **CS:** Chitosan**; PAA:** Poly­(acrylic acid)**; PMAA:** Poly­(methacrylic acid); **PBAE: Poly­(β-amino esters)**; **PDPA:** Poly­(2-diisopropylaminoethyl methacrylate); **PLGA–PEG–PA:** Poly­(lactic-*co*-glycolic acid)–polyethylene glycol–poly­(acrylic acid); **NPs:** Nanoparticles; **EVs:** Extracellular vesicles; **DMNs:** Dissolving Microneedles.

Despite compelling advances, pH-responsive adaptive polymers face several translational hurdles that complicate their clinical adoption. First, the heterogeneity and dynamism of physiological pH, spanning microenvironments such as inflamed tissues, tumors, gastrointestinal segments, chronic wounds, and intracellular vesicles, can blur design thresholds and undermine predictable responses. Polymers tuned to narrow pH windows often show context-dependent behavior, with variability driven by ionic strength, buffering capacity, local enzymatic activity, and protein corona formation. Moreover, dynamic *in vivo* processes, including protein corona formation, nonspecific adsorption, opsonization, and clearance by the mononuclear phagocyte system, can significantly alter polymer surface properties, responsiveness, and pharmacokinetics, resulting in variable biodistribution and reduced reproducibility across biological models.^295,296^ Immune recognition and long-term bioaccumulation further raise concerns regarding safety and chronic toxicity, particularly for high-molecular-weight or nondegradable systems. In drug delivery, burst release, incomplete cargo liberation, and diffusion-controlled leakage can occur if network mesh size changes are misaligned with payload dimensions or if polymer–drug affinity is inadequately engineered.

From a translational standpoint, manufacturing imposes its own constraints: batch-to-batch variability in molecular weight distribution, cross-linking density, and microstructure can shift transition pH and swelling ratios, complicating scale-up under GMP. Long-term implants and wound systems must also contend with biofouling, fibrosis, and immune modulation, where subtle changes in charge distribution or hydrophobicity alter host responses. Practical concerns, including sterilization compatibility (e.g., γ-irradiation, steam, EtO), storage stability, and integration with devices, often reveal performance drifts not captured in benchtop studies. Finally, the regulatory pathway for smart materials is complex: composite, multistimuli, or hybrid systems (e.g., polymer–nanoparticle–biologic combinations) require rigorous, multidomain characterization (toxicology, degradation products, immunogenicity, and function under clinically relevant pH cycles), which lengthens timelines and increases cost.

The next decade will likely see pH-responsive polymers evolve from single-trigger constructs into logic-gated, multistimuli platforms that integrate pH with redox, enzyme, CO_2_, hypoxia, or temperature cues for higher specificity in diseased microenvironments. Sequence-defined macromolecules and precision polyelectrolytes, enabled by controlled polymerization, click chemistry, and computational design, will allow fine-tuning of p*K*_a_, charge density, and cooperative transitions to achieve ultrasharp, hysteresis-minimized responses. Hybrid systems that combine pH-sensitive domains with biomimetic motifs (zwitterionic antifouling shells, adhesion peptides, or glycomimetic interfaces) can address immune compatibility while maintaining adaptive function. Advanced systems are increasingly being developed for protein and nucleic acid delivery, where pH-responsive polymer–biomacromolecule conjugates improve stability, circulation time, and intracellular trafficking by promoting endosomal escape under acidic conditions. Smart injectable depots and *in situ*-forming hydrogels, which undergo sol–gel transitions or network rearrangements in response to physiological pH changes, have shown strong potential for minimally invasive administration and long-term, localized release of proteins, peptides, and cells in regenerative medicine and cancer therapy. In tissue engineering, adaptive scaffolds incorporating pH-responsive motifs allow dynamic modulation of mechanics, degradation, and biofactor release, enabling closer mimicry of evolving pathological or healing microenvironments.^308–310^

A particularly promising direction is the development of theranostic pH-responsive polymers, where drug release is coupled with real-time imaging or sensing. Polymer–drug conjugates incorporating acid-labile linkers and fluorescent or photoactive backbones have demonstrated pH-triggered drug release with simultaneous optical reporting, facilitating treatment monitoring and personalized dose optimization.^311^ Furthermore, the integration of pH responsiveness with other endogenous cues, such as enzymatic activity or redox gradients, is driving the emergence of multistimuli adaptive materials capable of hierarchical or logic-gated responses. These systems offer enhanced selectivity and robustness in heterogeneous disease microenvironments, particularly in tumors and inflammatory tissues.^312^ In parallel, pH-responsive polymer–protein and polymer–drug conjugates are rapidly advancing as clinically relevant platforms in oncology and immunotherapy.^313,314^ Innovations in linker chemistry, sequence-controlled polymers, and controlled radical polymerization (e.g., RAFT) enable precise control over conjugation density, responsiveness, and degradation, thereby expanding therapeutic windows while minimizing systemic toxicity.^315–317^ Collectively, these approaches highlight a shift from simple stimulus-responsive carriers toward multifunctional, adaptive polymer systems that combine chemical precision, biological responsiveness, and diagnostic capability, key attributes for next-generation biomedical materials.

In therapeutics, codelivery architectures (e.g., micelles, nanogels, and injectable hydrogels) will enable spatiotemporally programmed release of synergistic payloads, small molecules, biologics, and gene therapies, with endosomal pH exploitation for intracellular delivery. 4D printing and microfluidic manufacturing will improve device integration and reproducibility, while organ-on-chip and adaptive *in vitro*-*in vivo* correlation (IVIVC) models will better capture pH oscillations and gradients, accelerating translation. Embedding sensing elements, including ratiometric fluorophores, conductive fillers, or photoacoustic probes, will foster closed-loop theranostics, where polymer response informs real-time clinical decisions. From a development standpoint, AI-guided materials discovery, high-throughput screening, and physics-informed models of swelling, diffusion, and drug–polymer interactions will shorten iteration cycles. Clinically, success will hinge on application-specific standardization (e.g., wound care vs GI delivery vs tumor therapy), GMP-aligned quality attributes (transition pH tolerance, fatigue resistance, sterilization resilience), and regulatory science frameworks for smart materials. Importantly, aligning polymer design with precision medicine, patient-specific pH phenotypes and disease-stage biomarkers will unlock genuinely personalized devices and formulations.

Future breakthroughs in pH-responsive adaptive polymers will be strongly propelled by advances in precision polymer chemistry, which are enabling unprecedented control over macromolecular primary structure, architecture, and responsiveness. Among these, sequence-controlled and sequence-defined polymer synthesis has emerged as a transformative tool, allowing explicit regulation of monomer order, charge distribution, and functional group placement along polymer chains. Such control enables predictable structure–function relationships, more biomimetic behavior, and fine-tuning of pH sensitivity, cellular interactions, and intracellular trafficking, capabilities that were previously inaccessible with conventional random or block copolymers.^318,319^ Recent studies demonstrate that sequence precision can directly influence protein binding, membrane interactions, and degradation profiles, positioning sequence-defined polymers as next-generation platforms for responsive drug and biomolecule delivery.^320^

Another rapidly advancing approach is dynamic covalent chemistry (DCC), which provides adaptive polymers with reversible, stimuli-responsive covalent linkages that reorganize under thermodynamic control.^321^ Incorporation of acid-labile or exchangeable bonds (e.g., imine, hydrazone, boronate ester, or disulfide linkages) enables polymers to undergo reversible structural rearrangement and self-regulation in response to pH changes, closely mimicking biological adaptability. DCC-based systems, including biodynamers and single-chain polymer nanoparticles, have shown enhanced intracellular delivery, endosomal escape, and controlled payload release in acidic microenvironments, highlighting their promise for pH-triggered therapeutics.^312,322^

In parallel, controlled radical polymerization techniques, particularly RAFT polymerization, continue to underpin the rational design of pH-responsive polymers by offering precise control over molecular weight, architecture, and functionality. RAFT enables the synthesis of block, gradient, and multiblock copolymers with narrowly distributed sizes and well-defined terminal groups, facilitating postpolymerization modifications such as protein conjugation and ligand attachment. Importantly, the thiol chain-end functionality generated via RAFT serves as a versatile handle for site-specific bioconjugation, allowing integration of proteins, peptides, and antibodies into responsive polymer systems with preserved bioactivity.^8,313^

Complementing these synthetic advances, bio-orthogonal conjugation chemistries, including strain-promoted azide–alkyne cycloaddition and thiol–maleimide coupling, have become essential for creating multifunctional pH-responsive polymer–drug and polymer–protein conjugates with minimal off-target reactivity. When combined with responsive linkers, these tools allow decoupling of polymer synthesis from biological functionalization, improving reproducibility and translational robustness.^308,323^ Finally, data-driven polymer design and machine-learning-assisted material discovery are beginning to influence the field by enabling the prediction of pH responsiveness, self-assembly behavior, and biological performance from molecular descriptors. Integration of high-throughput synthesis with computational modeling provides a powerful pathway toward rational, application-specific optimization, accelerating the identification of adaptive polymer systems suitable for clinical translation.^318,320^ ′Collectively, these emerging chemistry tools are shifting the field from empirically designed pH-responsive materials toward precision-engineered, adaptive polymer systems with predictable performance, multifunctionality, and improved translational potential, key requirements for next-generation biomedical materials.

In summary, pH-responsive adaptive polymers have instigated a fundamental shift in biomedical materials science, advancing the field from largely passive carriers toward dynamic, context-responsive systems capable of interpreting and acting upon local biochemical cues. By transducing subtle pH gradients into regulated changes in conformation, permeability, adhesion, and mechanical properties, these materials enable site-selective drug release, microenvironment-specific stabilization of bioactives, and adaptive interfaces for tissue repair, diagnostics, and implantable devices. Despite these advances, translation beyond proof-of-concept remains contingent upon deliberate engineering of stimulus selectivity, systemic stability, manufacturability, and long-term safety, supported by validation in physiologically relevant and predictive models. The field now resides at a pivotal convergence point, where precision polymer chemistry, integrative bioengineering, and data-driven design tools can be jointly leveraged to deliver reproducible, scalable, and regulatory-compliant solutions. As interdisciplinary collaborations mature, linking materials scientists, pharmacologists, clinicians, and regulatory stakeholders, pH-responsive polymers are well-positioned to evolve from experimental constructs into clinically deployable components of precision therapeutics and regenerative medicine. Sustaining this trajectory will require rigorous characterization standards, alignment with clearly defined clinical needs, and continued creativity in multiscale system integration.

## Data Availability

No primary research results, software, or code have been included, and no new data were generated or analyzed as part of this review.
